# Supplementary material for: Evolutionary dynamics of genome size and content during the adaptive radiation of Heliconiini butterflies
Source: Nat Commun. 2023 Sep 12;14:5620. doi: 10.1038/s41467-023-41412-5 (PMC10497600; doi:10.1038/s41467-023-41412-5)
Supplement: Supplementary file 1 — Supplementary Info [file 41467_2023_41412_MOESM1_ESM.pdf]

Supplementary Materials for

Evolutionary dynamics of genome size and content during the adaptive radiation of Heliconiini butterflies

Francesco Cicconardi, Edoardo Milanetti, Érika C. Pinheiro de Castro, Anyi Mazo-Vargas, Steven M. Van Belleghem, Angelo Alberto Ruggieri, Pasi Rastas, Joseph Hanly, Elizabeth Evans, Chris D Jiggins, W Owen McMillan, Riccardo Papa, Daniele di Marino, Arnaud Martin, Stephen H Montgomery

Corresponding authors: Francesco Cicconardi, francicco@gmail.co5m; Stephen H. Montgomery, s.montgomery@bristol.ac.uk

This file includes:

Supplementary Notes

Supplementary Note 1.Tribe Wide, High-Quality Genomic Resources for Heliconiini

Supplementary Note 2. Improved Resolution of Phylogenetic relationships and Signatures of Introgression

Supplementary Note 3. Genomic landscape of topology, introgression, and ILS

Supplementary Note 4. Evolution of Genome Size and Content

Supplementary Note 5. Expansion and Contraction of Gene Content

Supplementary Note 6. Selection Across Heliconiinae Genomes and the Heliconiini Radiation

Supplementary Note 7. Tribe Wide Genomics Highlight Candidate Genes for Derived Traits

Supplementary References

Supplementary Notes

Supplementary Note 1. Tribe Wide, High-Quality Genomic Resources for Heliconiini

Our sequencing and assembly strategies resulted in an extensive and high-quality genomic resource of 63 Nymphalid butterflies, of which, 58 belong to the eight Heliconiini genera (Fig. 1; supplementary data 1), representing ~75% of the recognised Heliconiini species [1]. These include 44 *Heliconius* including representatives of all the seven subclades, six species of *Eueides*, and one species for each of the genera *Philaethria*, *Dryadula*, *Dryas*, *Podothricha*, and *Dione*. We also obtained three genomes of *Agraulis*, nominally a single-species genus, which show high levels of divergence consistent with multiple cryptic species in the genus [2], and a close outgroup to Heliconiini, *Speyeria mormonia* (sub-family Heliconiinae, tribe Argynnini). Nine assemblies were generated using third generation sequencing read technology (either PacBio Circular Long Reads, PacBioHiFi, Oxford Nanopore Technology reads, or 10X Genomics Linked Reads), 29 were produced using available low-coverage short-read Illumina data (SRAs), assembled using a reference-based approach, and 10 previously available contig-level assemblies were curated to remove contaminants, merge haplocontigs, and then scaffolded using synteny maps with the most contiguous closely related species (Fig. 1c; supplementary data 1). Of the 63 genomes, 35 are chromosome- or sub-chromosome-level assemblies (final N50 > 5 Mbp; considering scaffolds longer than 10 kb), a further 21 are scaffold-level genome assemblies (N50 > 100 kbp), and only seven are sub-scaffold-level assemblies (supplementary data 1). The average scaffold N50 is ~6 Mbp. GC content was consistent across taxa and all genomes were highly AT rich (average GC% = 33%) (supplementary Fig. 7). Overall, no sign of a W chromosome was evident from our analyses, with the expectation of the previously described *Dy. Iulia* W[3]. Genome assembly completeness, in terms of expected gene content, was evaluated by Benchmarking Universal Single-Copy Ortholog (BUSCO) analysis[4], and showed high completeness scores (complete average 93%, complete single copy 92%; Fig. 1, supplementary Fig. 8; supplementary data 1), with 52 genomes having a score >90%, and one, *Dr. phaetusa*, being 100% complete with 99% BUSCO genes in single copy, and almost all with very low level of duplications (average 0.9%; supplementary data 1). Within Heliconiini, transposable element (TE) annotation showed values in line with previous literature[5,6]. The total TE content ranged from 15% (43.7 Mb) in *A. v. maculosa* to 36.8% (162.4 Mb) in *H. telesiphe*. TE content is highly correlated with genome size (Fig. 1a; Pearson's ρ = 0.87), and explains more variation in genome size than variation in coding sequence (CDS) (Fig. 1b; Pearson's ρ = 0.52) or intronic sequence do (supplementary Fig. 18a; Pearson's ρ = 0.66). Within different genomic features the retroelements seem to be the major contributor to genome size (Random Forest, ntree=1M; supplementary Fig. 9), followed by intron size (supplementary Fig. 18a) and total interspersed repeats (supplementary Fig. 11).

To provide a foundation for downstream analyses, we not only annotated protein content in all 63 genomes, but used a combinatorial source of annotation to maximise each methodological approach, with

a final cross-species annotation to remove possible species-biases. The annotation pipeline (supplementary Fig. 2) was based on a combination of homologous protein mapping, gene prediction, *ab initio, de novo*, and transcript evidence, using new transcriptomic data (*Dr. phaetusa, Dy. iulia,* and *Di. juno*) and leveraging extensive RNA data already available for *Heliconius*. These data were used to re-annotate (for *H. erato demophoon* and *H. melpomene*, with 87 and 128 short read archive (SRA) datasets, respectively) or annotate available genomes (for *H. cydno* and *H. sara*, with 20 and 6 SRA datasets, respectively). The annotation pipeline includes the Comparative Annotation Toolkit (CAT)[7], which combines a variety of parameterizations of AUGUSTUS, and uses TRANSMAP to project reference annotations throughout a whole-genome alignment[8] to synthesize all annotation methods (supplementary Fig. 3). The resulting annotations have an average of 20,559 protein-coding genes per species (standard deviation of 4,052; supplementary data 1) and the distribution of mRNA, exon, and CDS length showed high consistency across taxa. Intron distributions showed higher variability across species, in line with previous data[5] (supplementary Fig. 12-15). Locus completeness, calculated by aligning amino-acid sequences to UNIPROTKB (see Methods), also showed high consistency across taxa, except for four species of *Eueides* species which are more skewed towards a lower degree of alignment due to lower contiguity (supplementary Fig. 16). During orthology searches, chimeric loci were also corrected, this affected a minor proportion of genes (average of 1.14±0.7% of chimeric loci per 100 loci) and tended to occur in regions of high gene density (Pearson's ρ = 0.52). Compared to two previous annotations (*H. erato demophoon* v.1 and *H. melpomene* v.2.5) our re-annotations not only generated more consistency between species, but also significantly fewer short alignments between homologous sequences (supplementary Fig. 17).

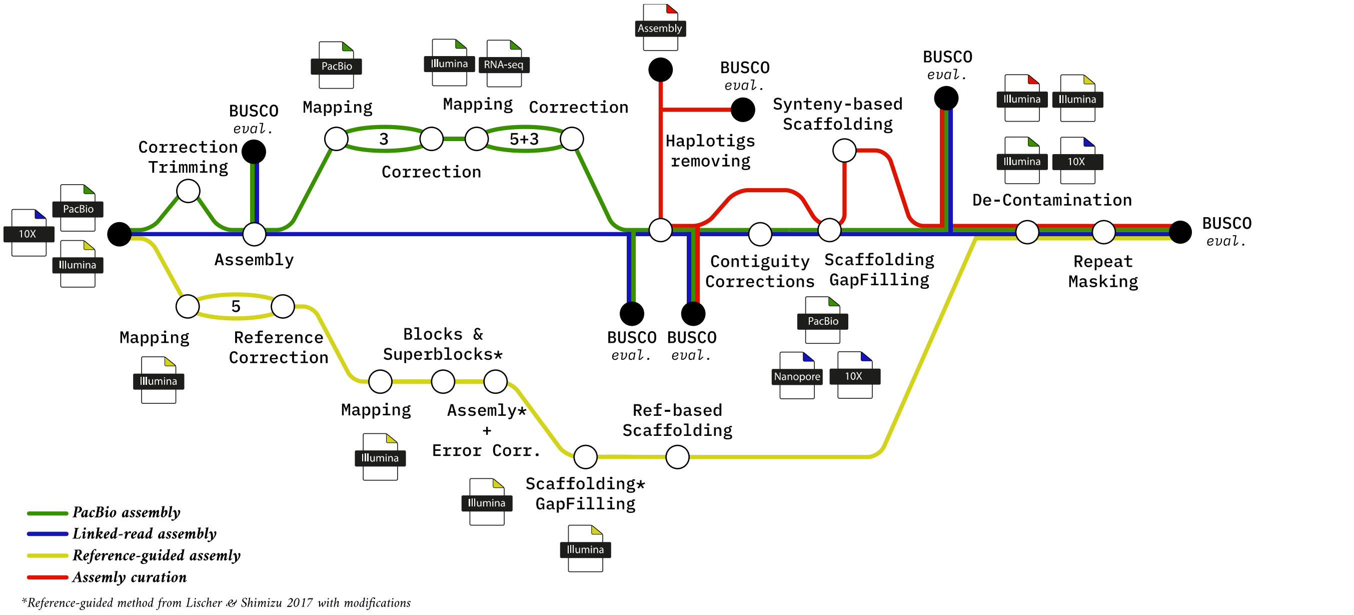

Supplementary Fig. 1 | Assembly pipelines. *Schematic diagrams showing the different approaches (colour) according to the type of data and task.*

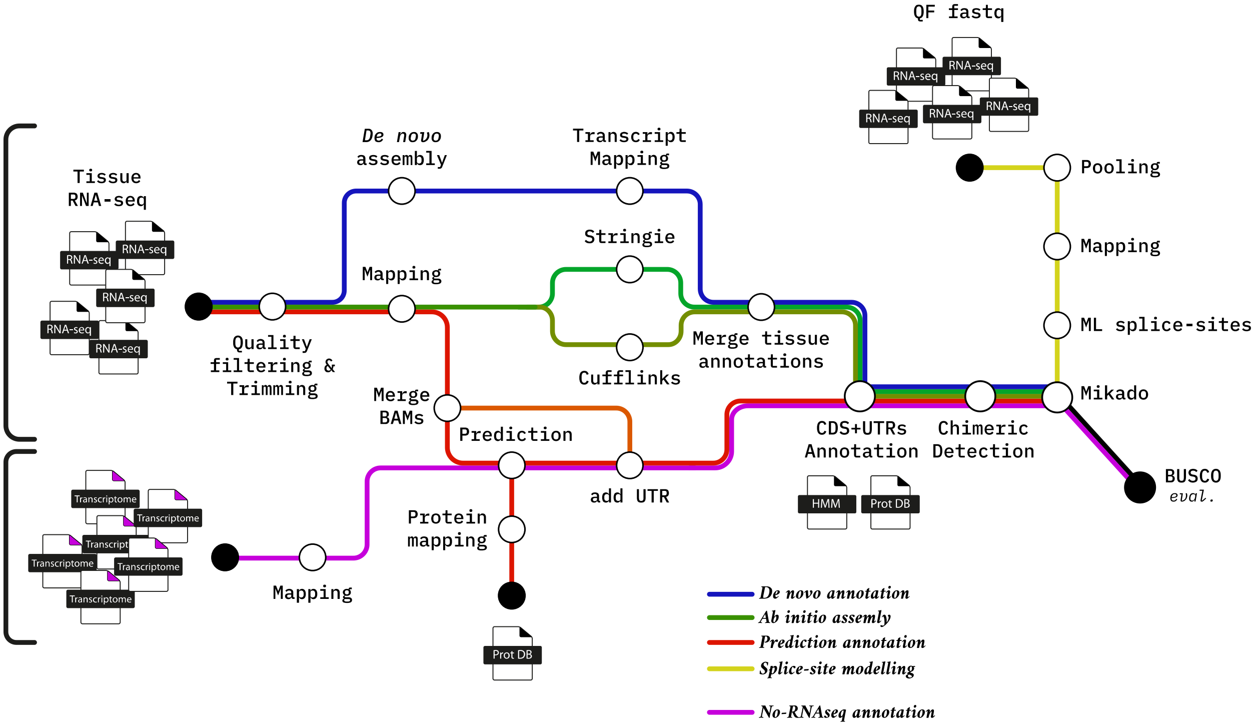

Supplementary Fig. 2 | Annotation pipelines. *Schematic diagrams showing the annotation approaches (colour) for species for which RNA-seq data was available, and when it was not (purple).*

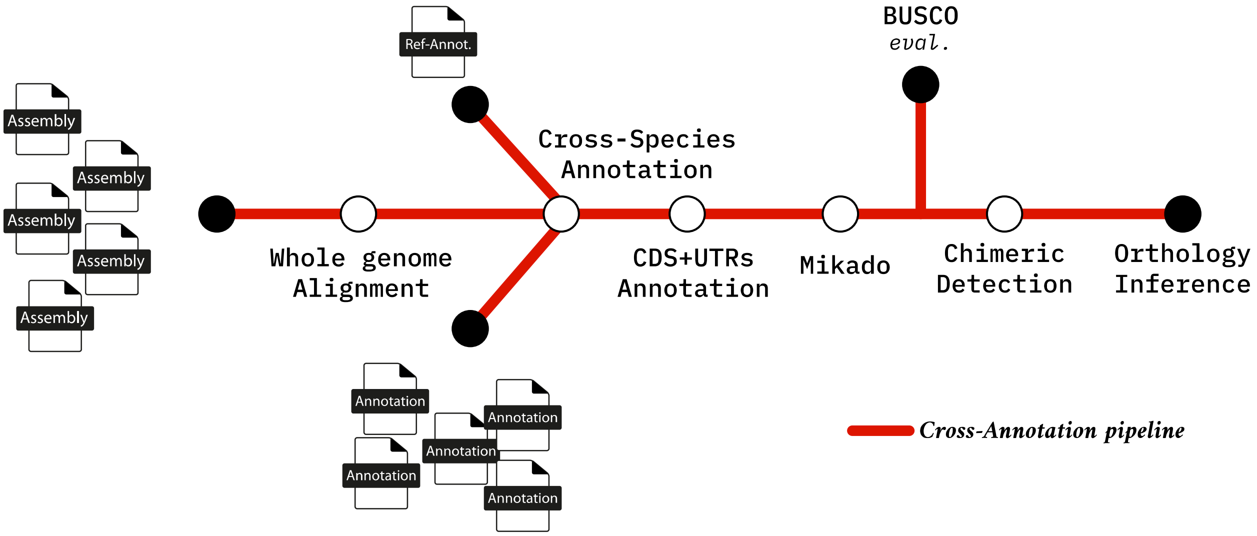

Supplementary Fig. 3 | Comparative annotation pipeline. *Schematic diagram showing the final step of the annotation which include the Comparative Annotation Toolkit (CAT), and the following steps, which include the artificial chimeric correction and the orthology inference.*

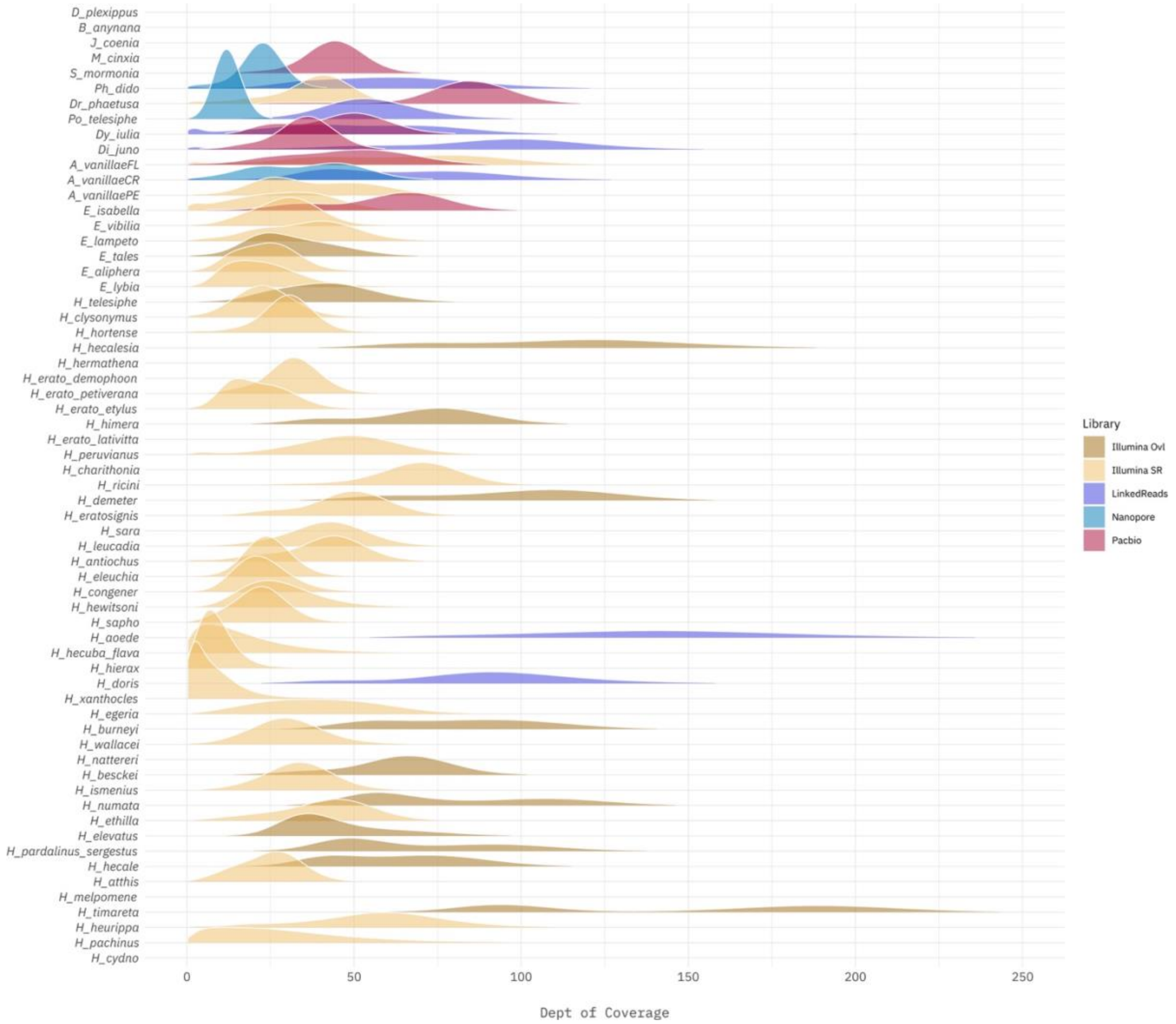

Supplementary Fig. 4 | Depth of coverage. *For each dataset assembled in this study the plot shows the distribution of coverage for the different sequencing strategy (colour).*

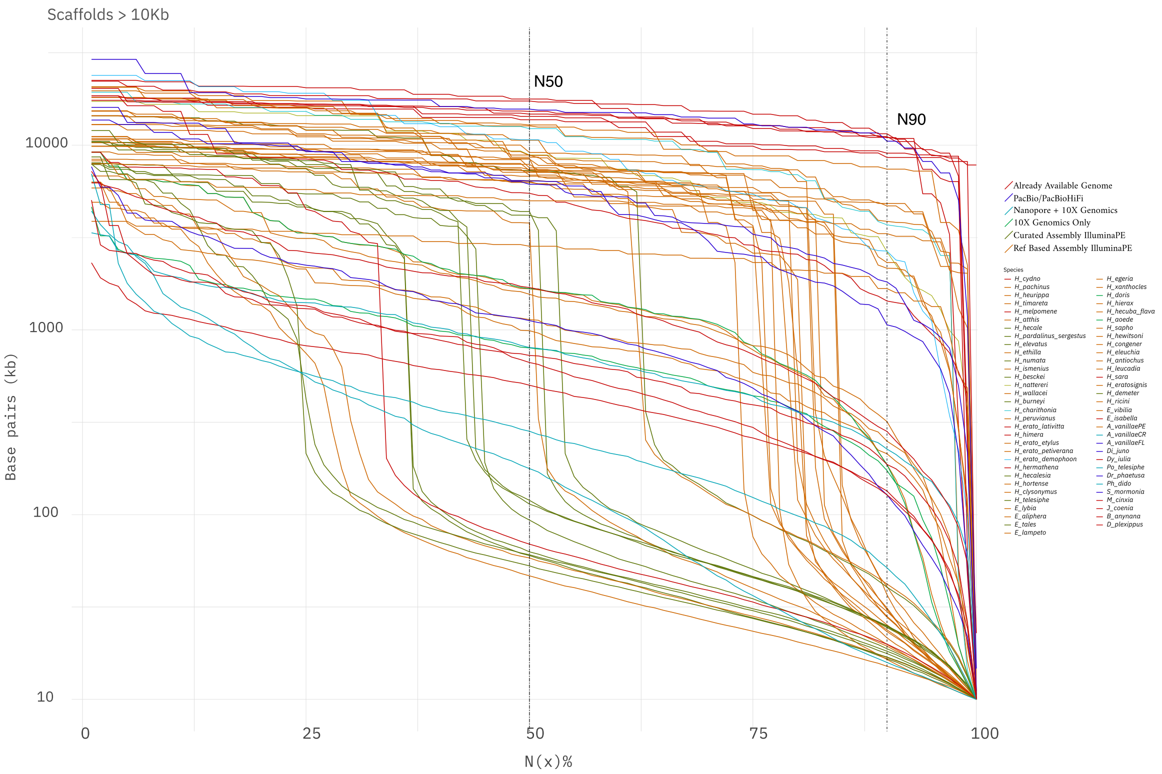

Supplementary Fig. 5 | N(x) distributions across genome assemblies grouped by assembly strategy. *The plot shows the contiguity for each genome according to its sequencing/assembly strategy. Note how the great majority of them are distributed on the upper side of the plot with a N50 > 1000. Only few have low contiguities and correspond to the reference-based assemblies, which have a low depth of coverage of short Illumina reads. Dashed lines correspond to N50 and N90.*

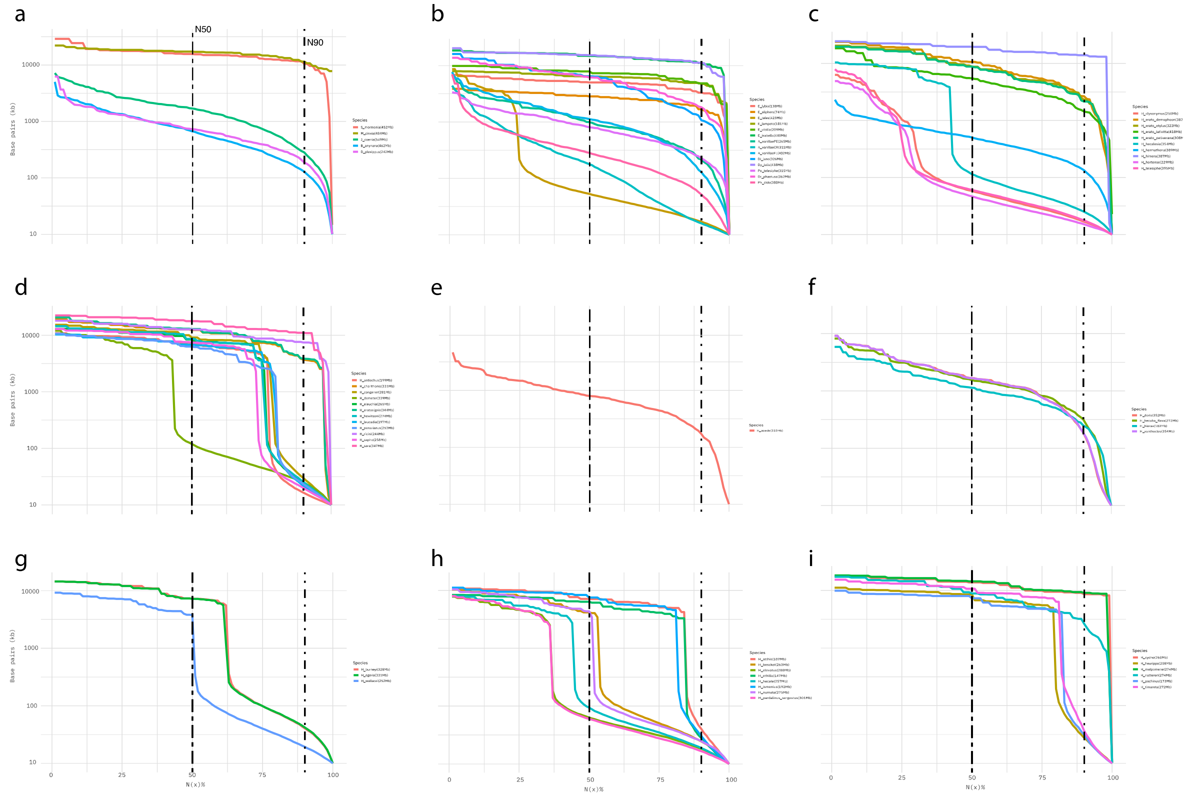

Supplementary Fig. 6 | N(x) distributions across genome assemblies grouped by clade. *The plot shows the contiguity for each genome according to its clade: (a) Outgroup species; (b) Heliconiini non-Heliconius species; (c) Erato clade; (d) Sara/Sapho clade; (e) H. aoede; (f) Doris clade; (h) Wallacei clade; (h) Silvaniform clade; (i) Melpomene clade. Dashed lines correspond to N50 and N90. In each clade there are more than one very contiguous assembly (N50 ~ 1Mb).*

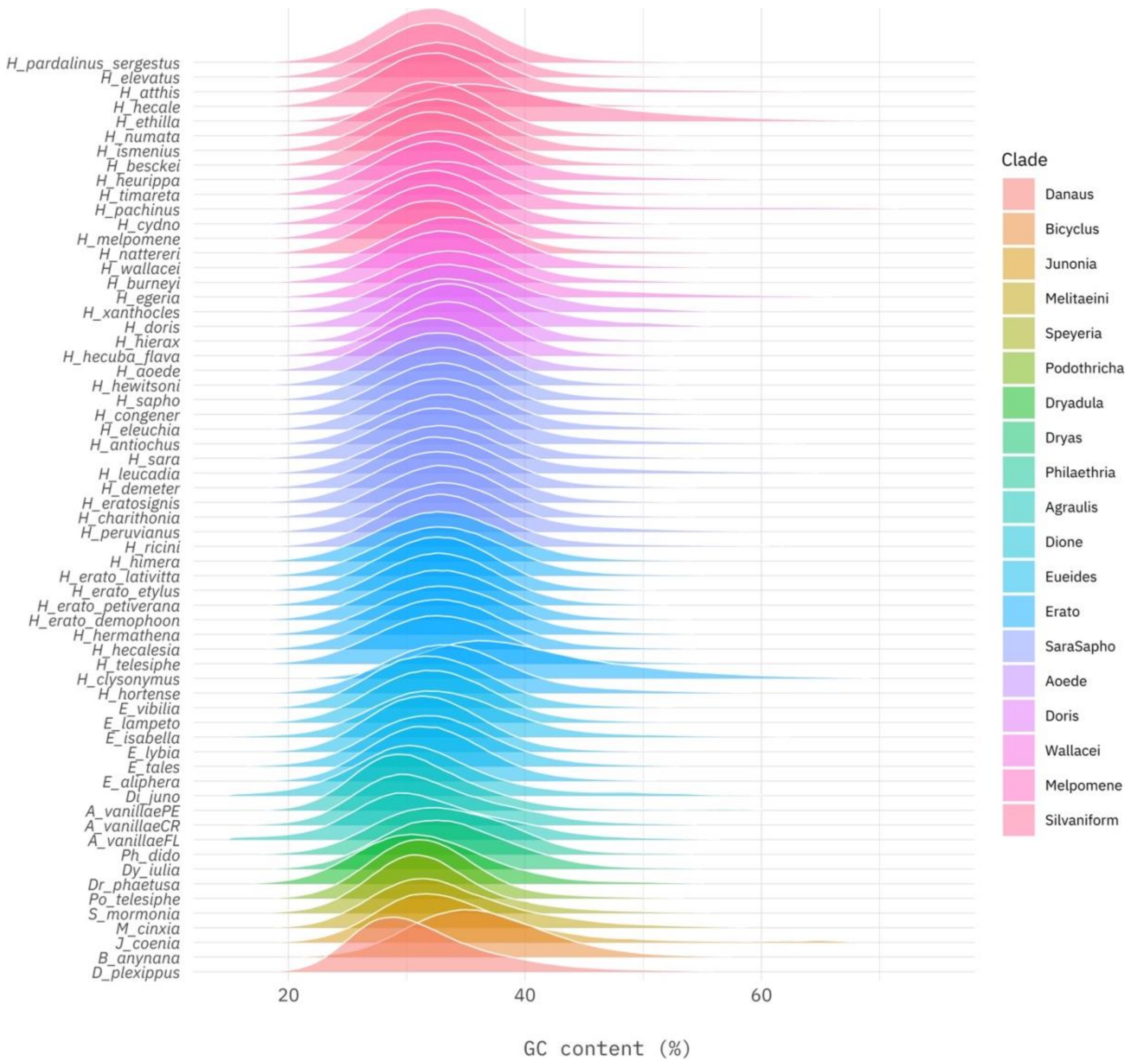

***Supplementary Fig. 7 | CG distribution**. For each dataset assembled in this study the plot shows the distribution of coverage for the different sequencing strategy (colour).*

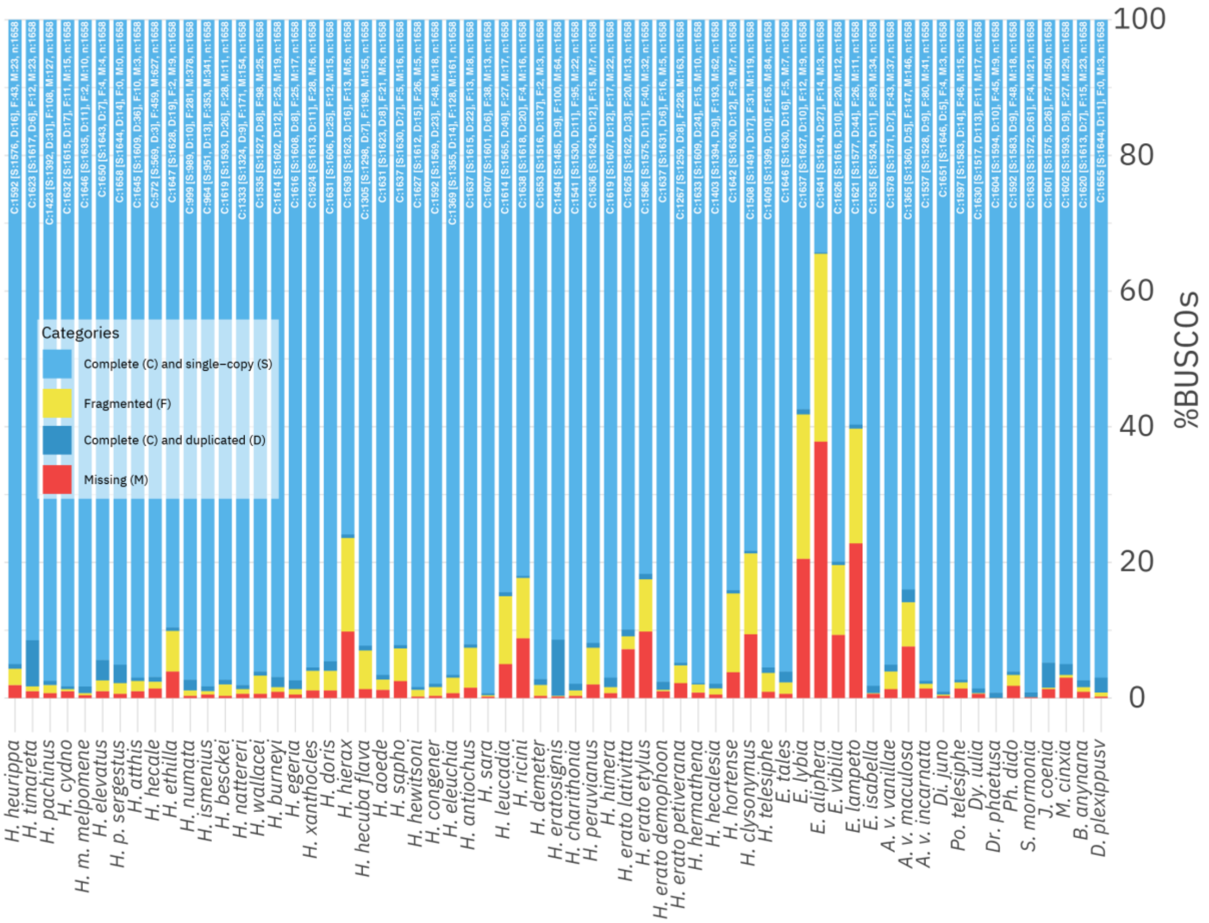

Supplementary Fig. 8 | BUSCO Assessment Results. *BUSCO profile across the dataset shows a remarkable completeness for the great majority of assemblies having a complete and single > 90%. Within Heliconius species very few have lower scores, followed by three of the six Eueides species.*

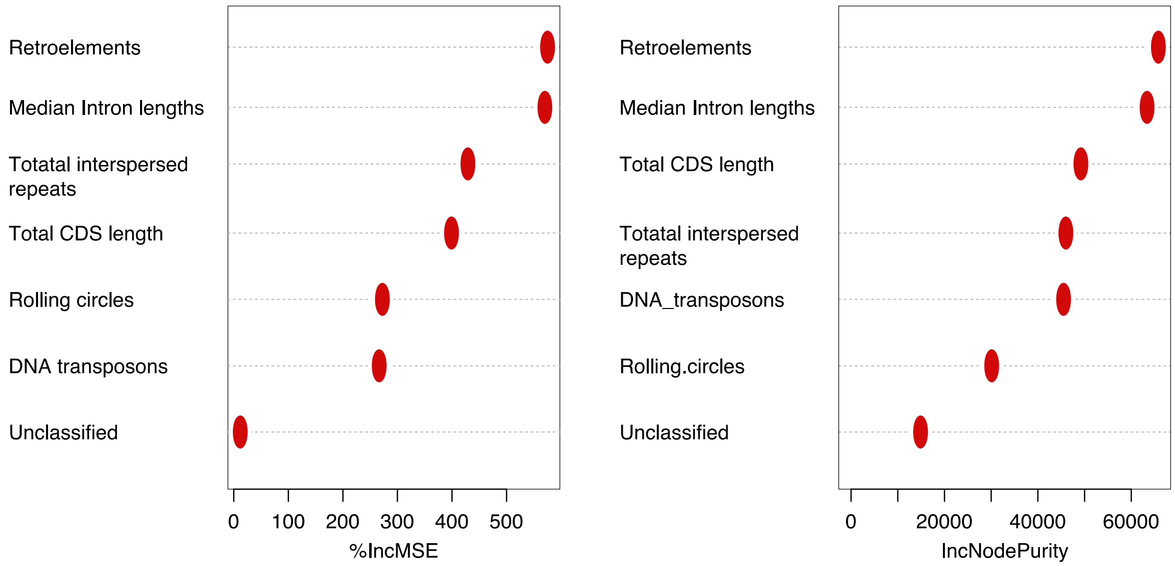

***Supplementary Fig. 9 | Genome size main factors**. The plots show random forest analysis (ntree=1,000,000) using different genomic features. The analysis underling both Retroelements and introns as the features most responsible, followed by Interspersed repeats and CDS regions, for the genome size variation among Heliconiini.*

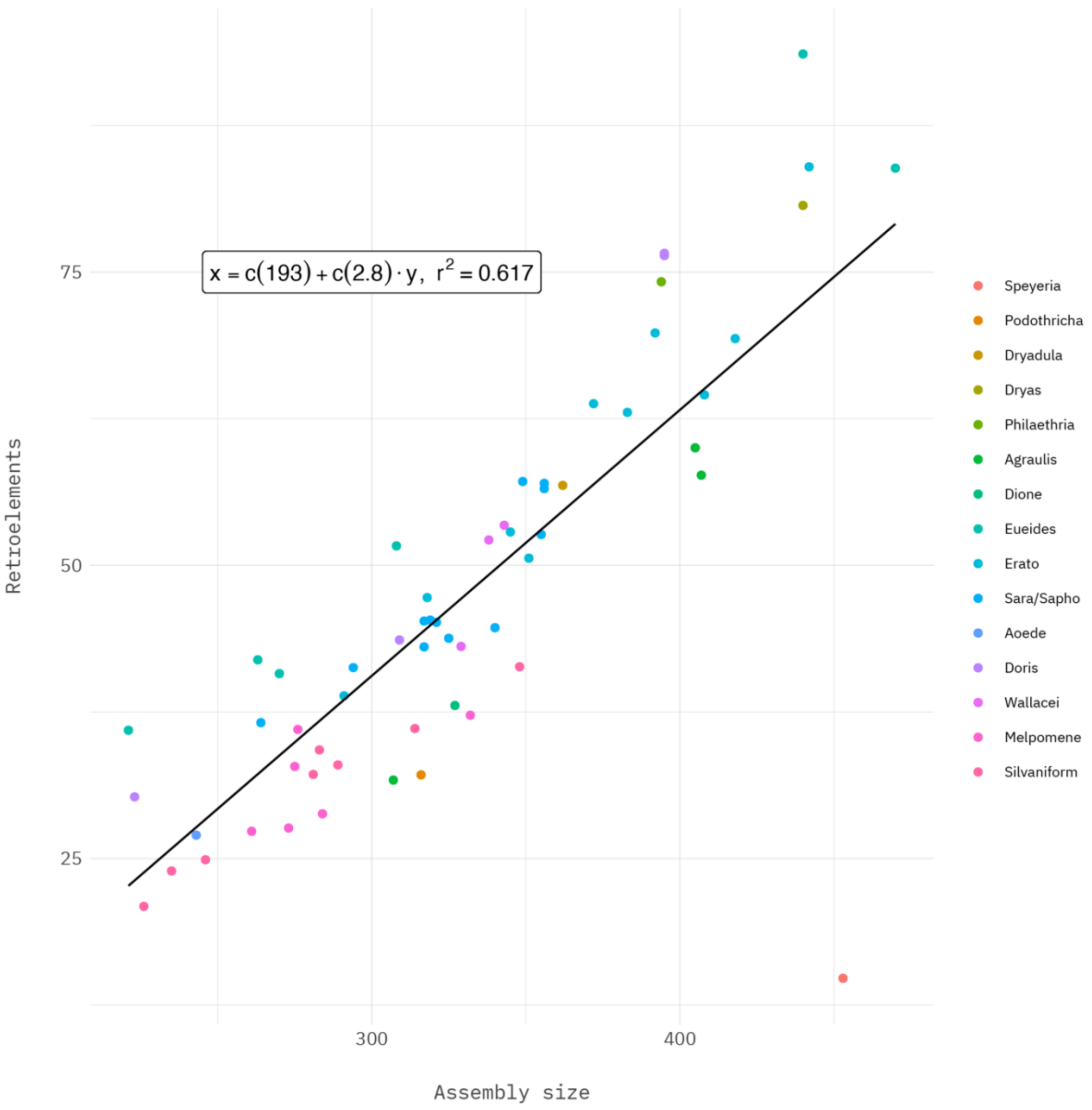

Supplementary Fig. 10 | Retroelements vs Genome size. *Correlation between retroelements and genome size indicate a great correlation between the two within Heliconiini (Pearson's ρ=0.78; R^2=0.62).*

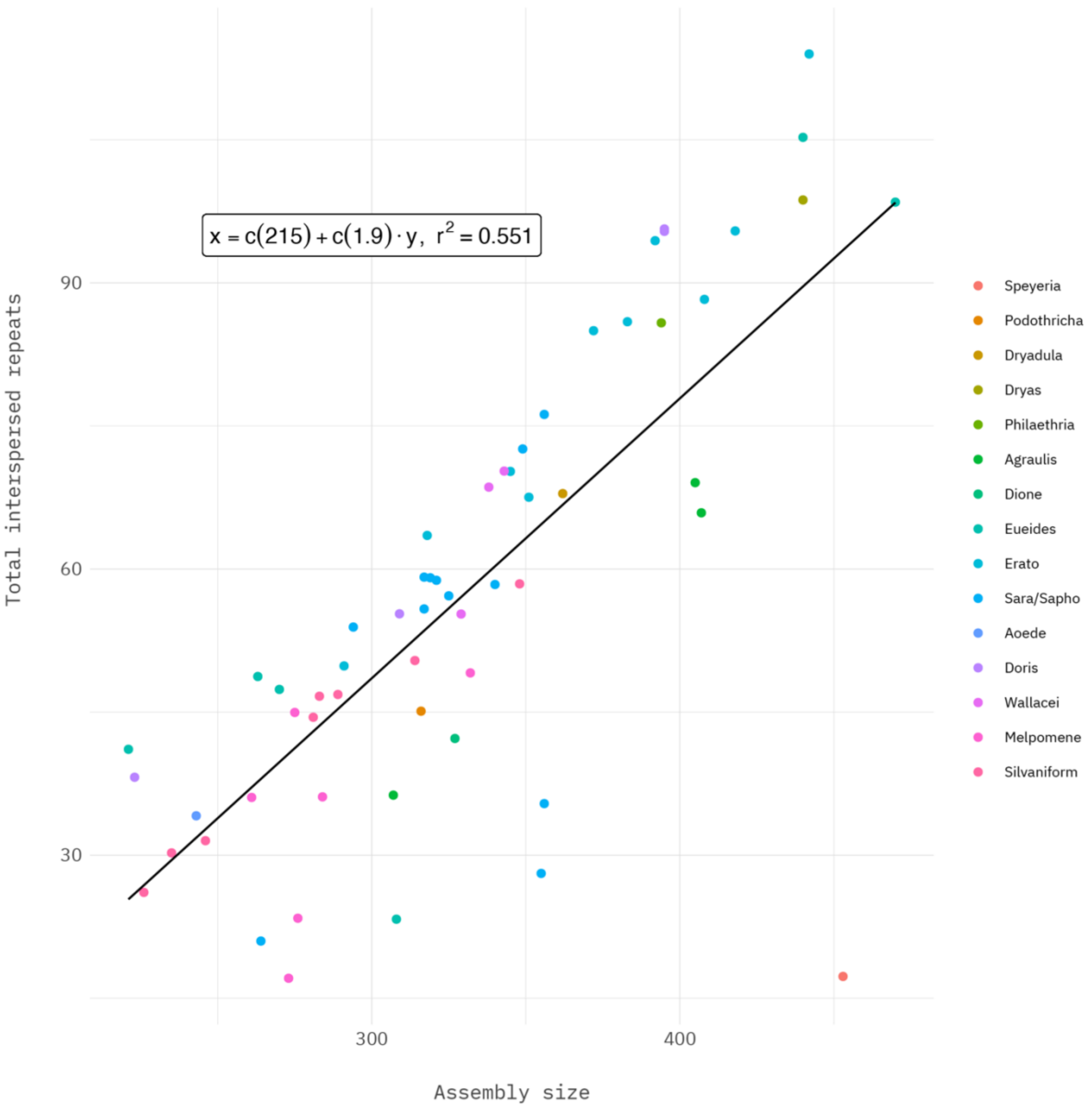

Supplementary Fig. 11 | Total interspersed repeats vs Genome size. *Correlation between total interspersed repeats and genome size indicate a good but lesser correlation between the two within Heliconiini compared with retroelements (Pearson's* ρ*=0.74;* R^2*=0.55).*

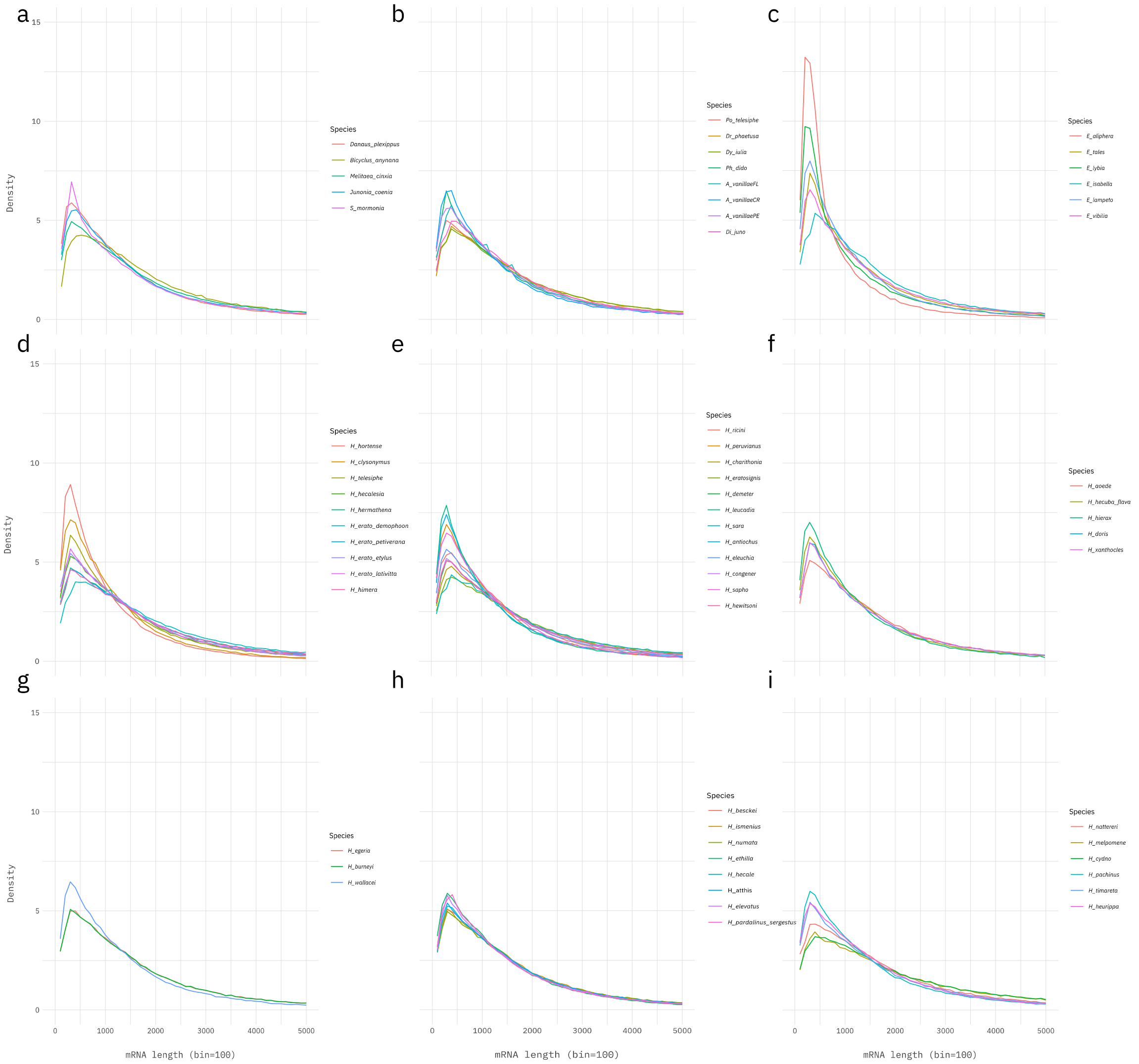

Supplementary Fig. 12 | mRNA length distributions. *mRNA Length distributions for the entire Nymphalid dataset divided by clades. (a) Outgroup species; (b) Heliconiini non-Heliconius and non-Eueides species; (c) Eueides species; (d) Erato clade; (e) Sara/Sapho clade; (f) H. aoede and Doris clade; (g) Wallacei clade; (h) Silvaniform clade; (i) Melpomene clade. A strong consistency can be observed across taxa, with very few exceptions, mainly in Eueides species where the genome fragmentation is impacting of the full-length loci reconstructions.*

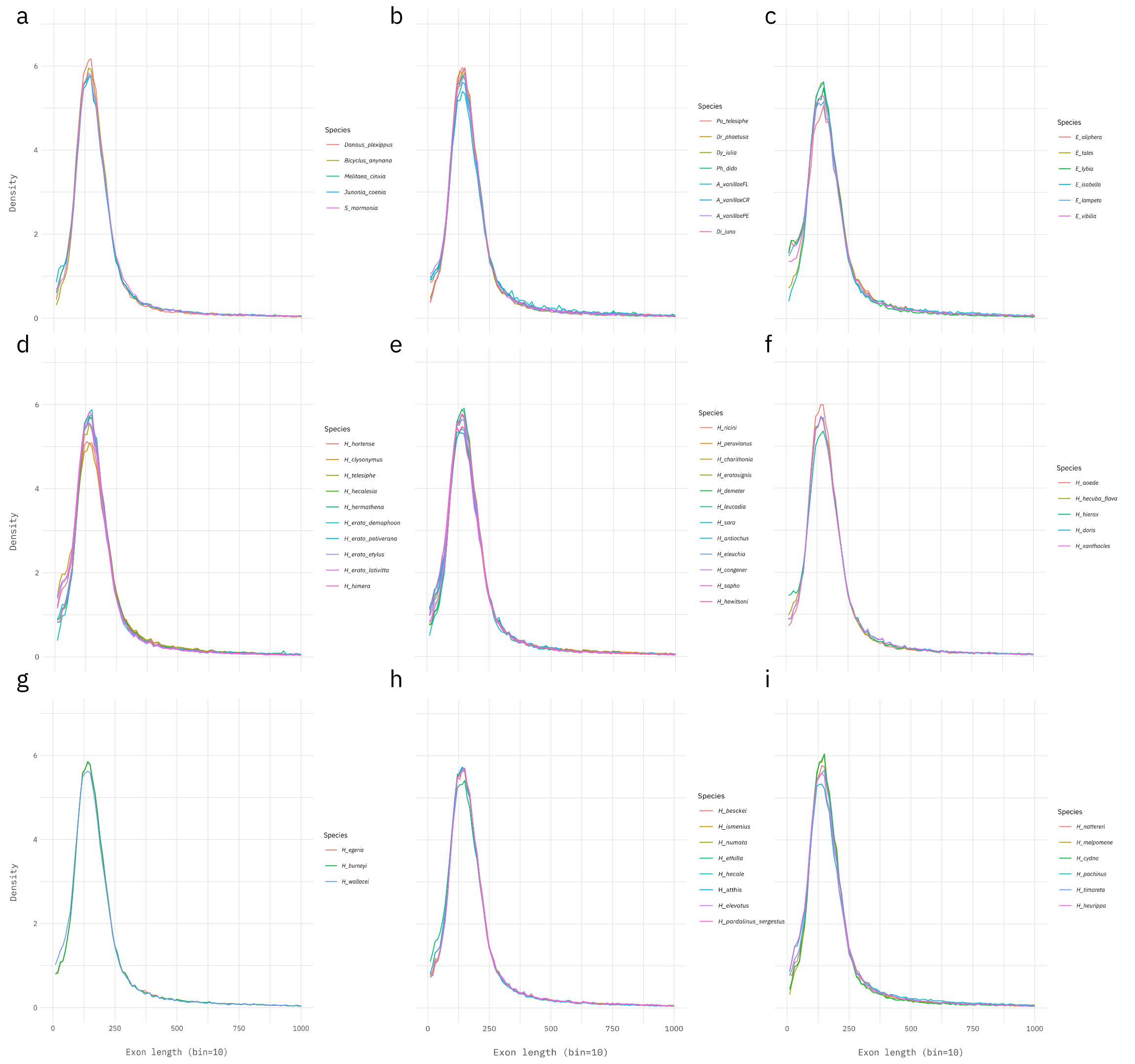

Supplementary Fig. 13 | Exon length distributions. *mRNA Length distributions for the entire Nymphalid dataset divided by clades. (a) Outgroup species; (b) Heliconiini non-Heliconius and non-Eueides species; (c) Eueides species; (d) Erato clade; (e) Sara/Sapho clade; (f) H. aoede and Doris clade; (g) Wallacei clade; (h) Silvaniform clade; (i) Melpomene clade. A strong consistency can be observed across taxa.*

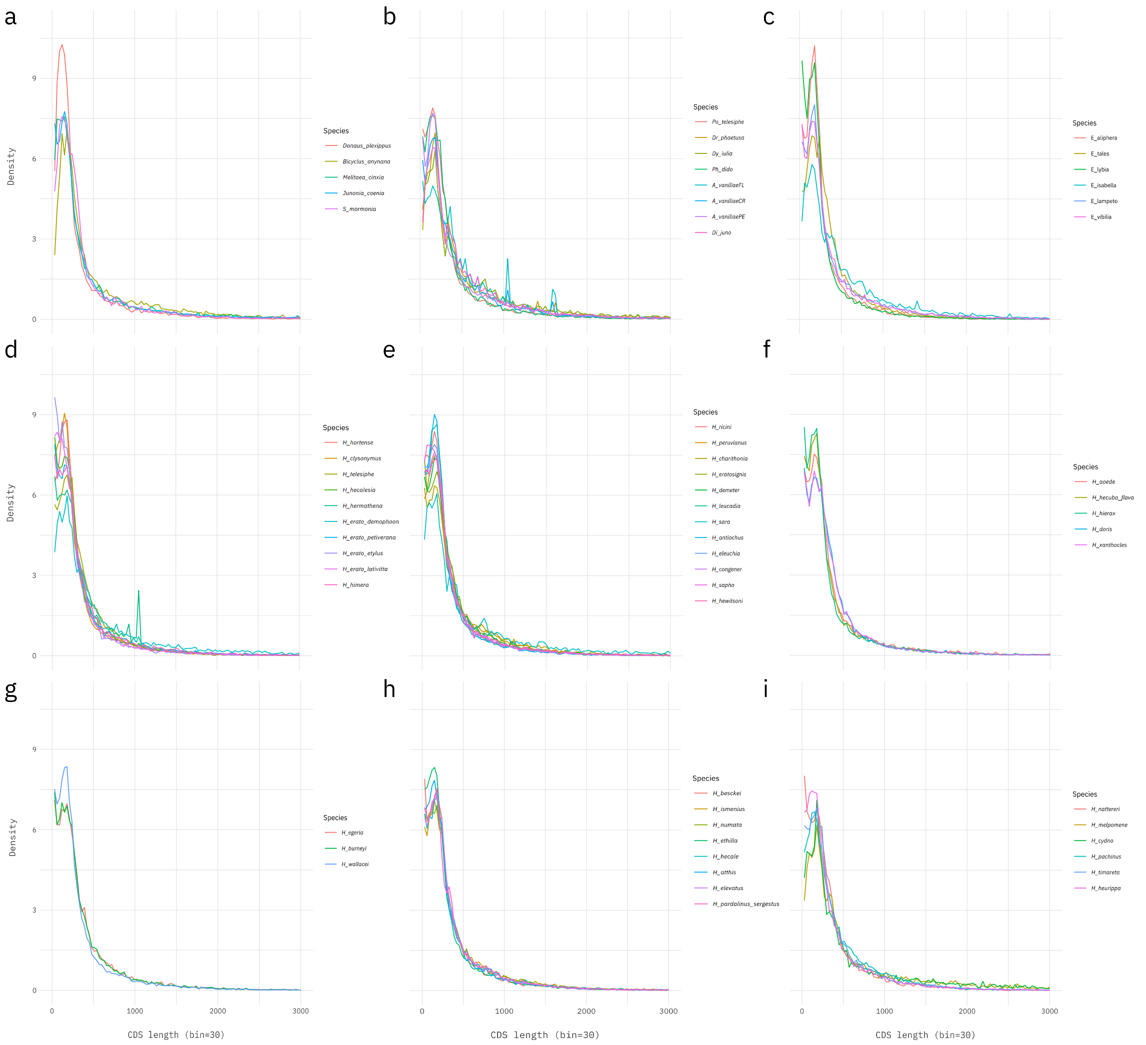

Supplementary Fig. 14 | CDS length distributions. *CDS Length distributions for the entire Nymphalid dataset divided by clades. (a) Outgroup species; (b) Heliconiini non-Heliconius and non-Eueides species; (c) Eueides species; (d) Erato clade; (e) Sara/Sapho clade; (f) H. aoede and Doris clade; (g) Wallacei clade; (h) Silvaniform clade; (i) Melpomene clade. A strong consistency can be observed across taxa, with very few exceptions (i.e.: Danaus plexippus).*

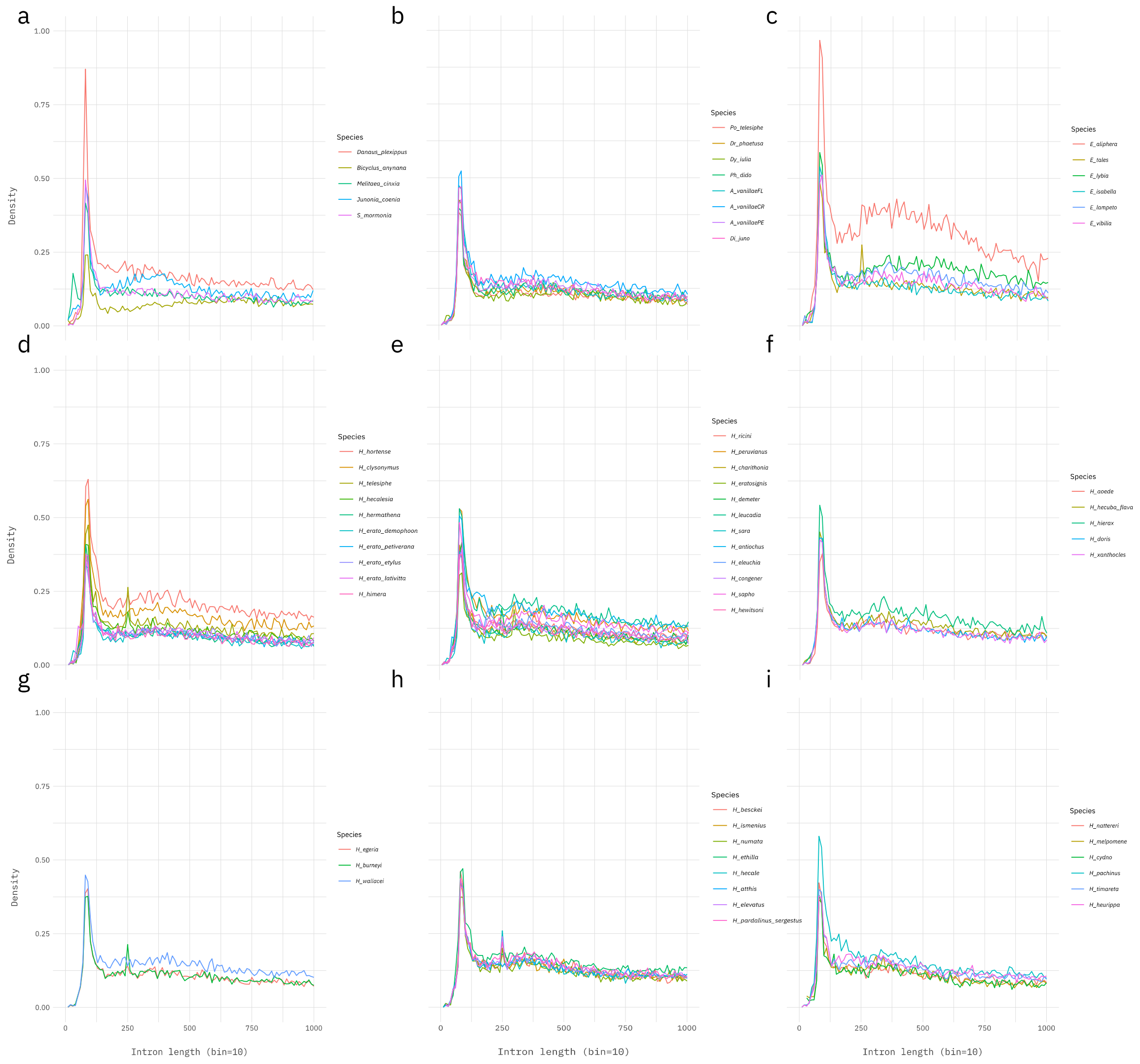

Supplementary Fig. 15 | Intron length distributions. *Intron Length distributions for the entire Nymphalid dataset divided by clades. (a) Outgroup species; (b) Heliconiini non-Heliconius and non-Eueides species; (c) Eueides species; (d) Erato clade; (e) Sara/Sapho clade; (f) H. aoede and Doris clade; (g) Wallacei clade; (h) Silvaniform clade; (i) Melpomene clade. This genomic feature seems to be the most variable among species compared with others. Apart for possible outliers (i.e.: E. aliphera), this could be the effect of transposable element as we show in Fig. 3 in the main text.*

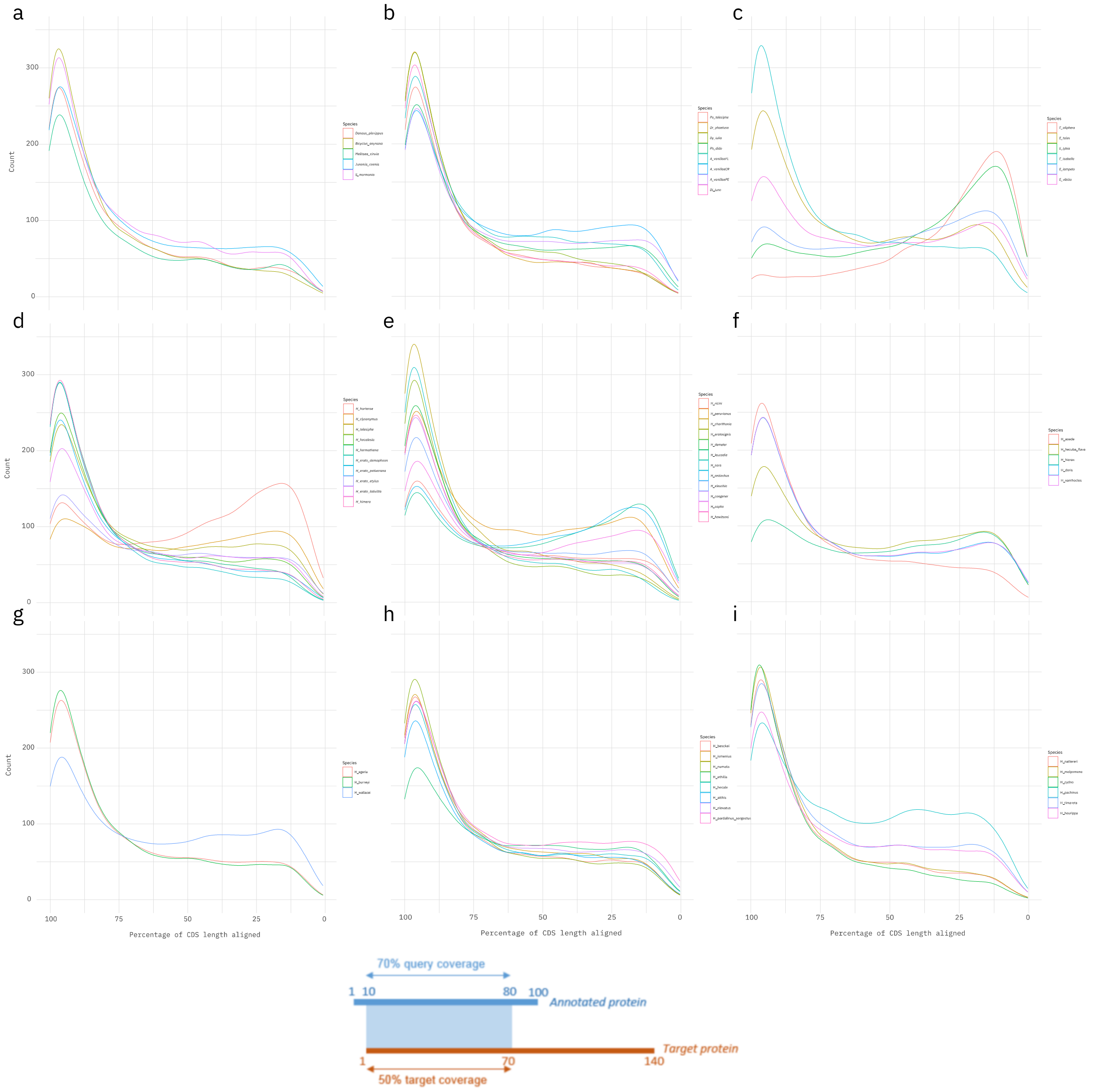

Supplementary Fig. 16 | Transcriptome completeness assessment. *The final set of annotated proteins, single protein for each locus, was searched with DeltaBLAST against Uniprot DB protein. For each hit the target coverage was calculated as the percentage of the target length that is included in the alignment. The plots show the distribution of the target coverage for each annotation. For almost all annotations the great majority of the transcripts are accumulating towards the 100% (left side of the x-axes). The target coverage is the percentage of the target length that is included in the alignment. (a) Outgroup species; (b) Heliconiini non-Heliconius and non-Eueides species; (c) Eueides species; (d) Erato clade; (e) Sara/Sapho clade; (f) H. aoede and Doris clade; (g) Wallacei clade; (h) Silvaniform clade; (i) Melpomene clade. For H. Melpomene and H. erato the previous annotations are also included, in both cases the distributions of the new annotations are more skewed towards the left side (completeness) of the x-axis.*

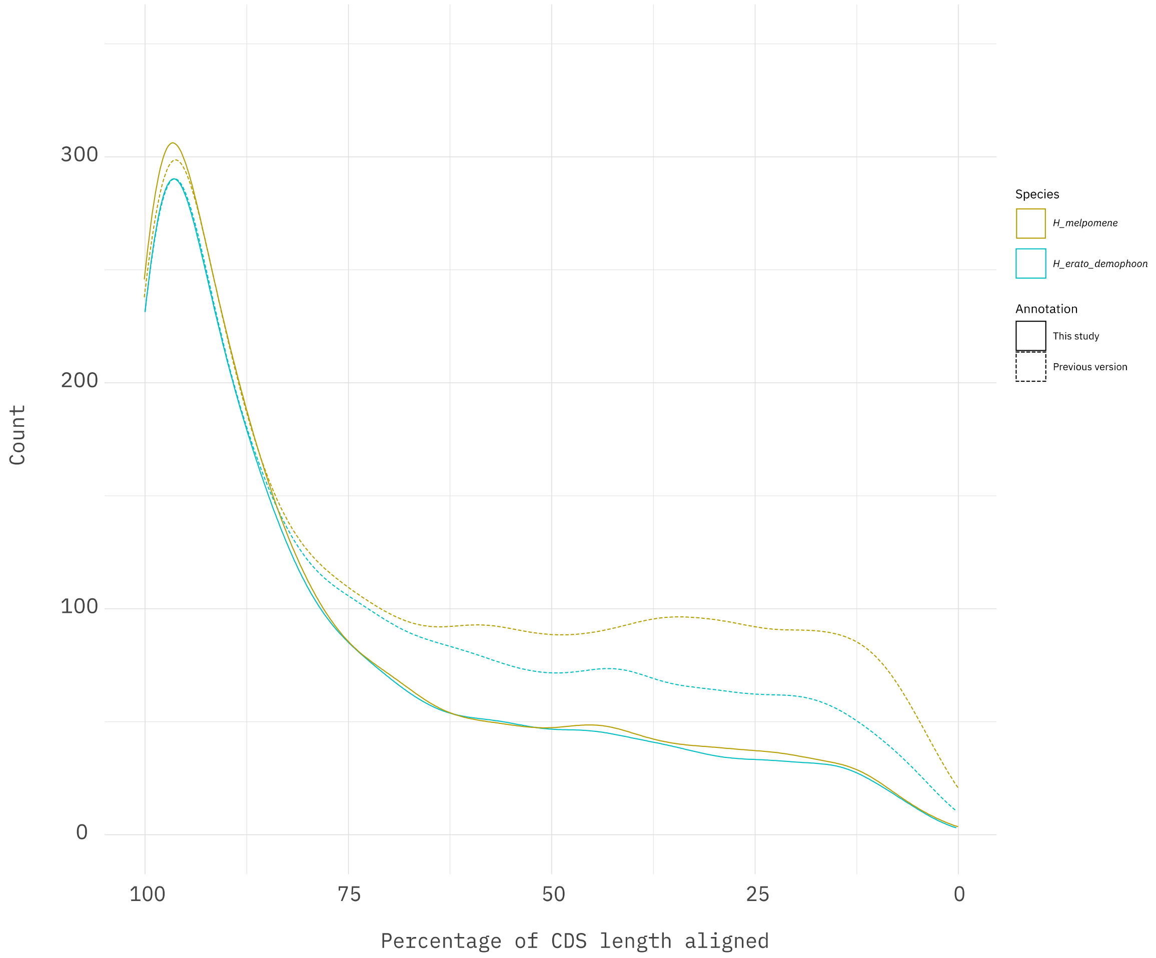

Supplementary Fig. 17 | Comparison with previous H. erato/melpomene annotations. *The plots show the distribution of the transcript completeness with its homologous in the Uniprot DB. The comparison is between the previous annotation version (dashed line) with the one generated in this study (continuous line). Note that for the previous annotation of H. erato all transcripts were used as it was difficult to extract the longest transcript per locus due to its transcript id name. For the other annotation the longest transcript per locus was used. The two new version have an overall reduction of short (probably incomplete) transcript (right side of the distribution). In H. melpomene can also be appreciated how the pick with the more complete transcript (left side of the distribution) is also higher than the previous version. To note also the higher consistency between the two species in the new version of the annotation.*

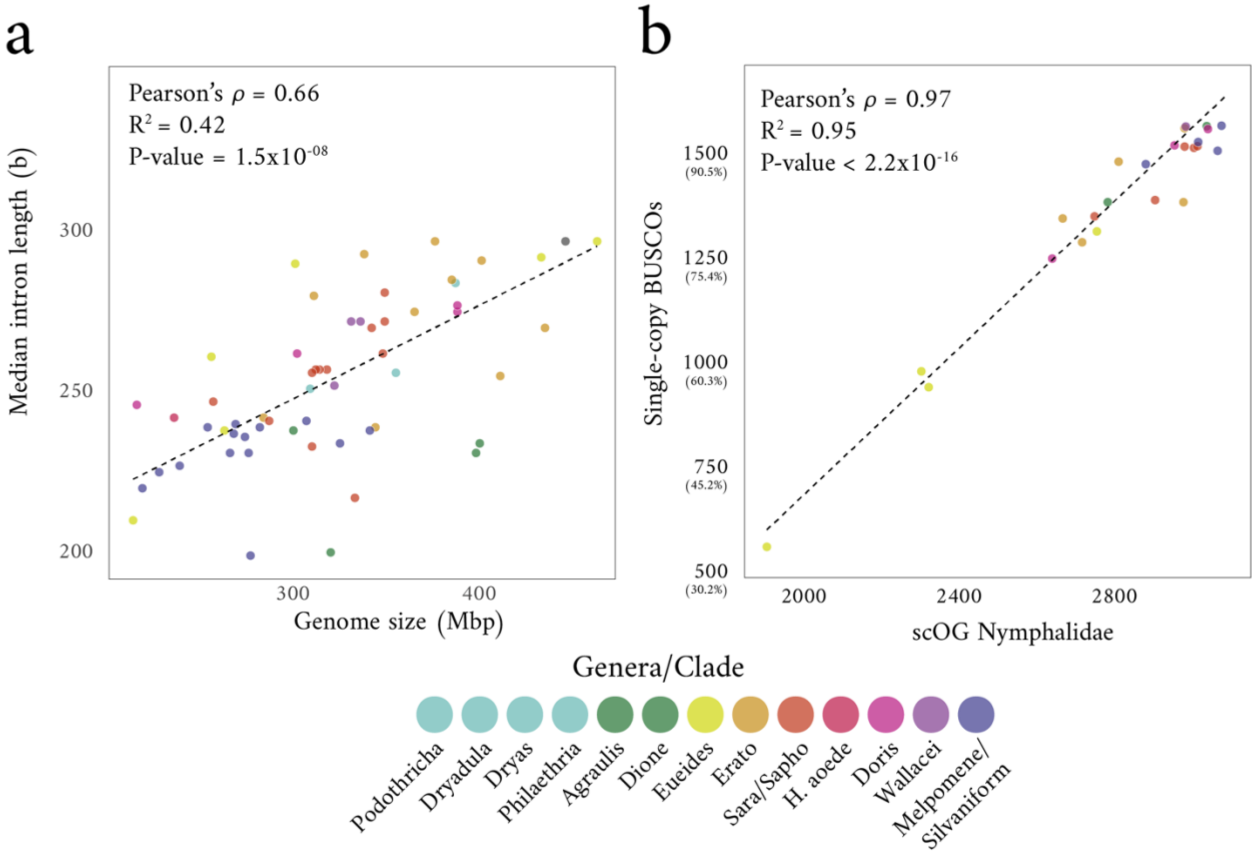

Supplementary Fig. 18 | Intron size vs Genome size and single copy relations with BUSCOs**.** ***a*** *Scatter plot of median intron length and genome sizes showing a robust correlation between the two (Pearson's ρ=0.66; R^2=0.42). Among all genomic components (TE classes, CDS and introns) intron size and Retroelements seem to contribute most to genome size variation (Random forest analysis; ntree=1M).* ***b*** *Correlation between single-copy BUSCO genes and scOGs indicate how the great majority of the genomes are highly complete (Pearson's ρ=0.97; R^2=0.95). In both plots data were fitted to a linear model (lm; P values ≤ $1.5x10^{-08}$).*

Supplementary Note 2. Improved Resolution of Phylogenetic relationships and Signatures of Introgression

The orthology-search analysis (see Methods) produced a total of ~34k orthologous groups (OGs), 65% of them in single copy (scOGs). Of these, 3,393 were conservatively identified across all *Heliconius* and *Eueides* clades (see Methods) and were used for phylogenetic analysis. The resulting topology and divergence dates (Fig. 1c, supplementary Fig. 19, 20, supplementary data 3) are largely consistent with previously inferred phylogenetic relationships[1,9–11]. However, the new topology shows marginal differences within some *Heliconius* clades (*e.g.*, paraphyly of the Silvaniform/Melpomene) and among other genera of Heliconiini. We now identify *Po. telesiphe* and *Dy. iulia* as sister lineages, outgrouped by *Dr. phaetusa* and then *Ph. dido*. We estimate the subfamily Heliconiinae originated ~45.3 million years (Mya) (95% CI: 35.9-55.5), with the last common ancestors of *Eueides* and *Heliconius* dating to ~11.1 Mya (95% CI: 7.3-12.1) and 9.6 Mya (95% CI: 8.8-13.8), respectively. We also find evidence of a higher molecular substitution rate (number of substitutions per site^{-Mya}) for Heliconiinae, Heliconiini, *Eueides* + *Heliconius, Eueides* and *Heliconius* branches

(0.49, 0.52, 0.43, 0.61, 0.61, median 0.37), compared with the overall distribution (Fig. 1c, supplementary data 3), indicating a series of bursts in evolutionary rate, beginning at the base of the radiation.

Of critical importance for understanding the adaptive evolution of *Heliconius*, and the set of traits linked to pollen feeding, is the position of the Aoede clade, which do not pollen feed but seems to be grouped within *Heliconius* in our topologies (Fig. 1c, 2b, supplementary Fig. 19-21), in agreement with previous molecular trees[1]. It remains possible that gene-tree species-tree discordance, caused by ILS and/or gene flow, could suggest a more complex pattern of divergence around these, or other, nodes which could alter interpretations of this topology. We therefore next examined evidence for these phylogenetic patterns.

First, to quantify the level of ILS within the phylogeny using, a coalescent summary method for species tree using ASTRAL-III (Fig. 2b). This resulted in an almost identical topology with the ML tree (Fig. 1c, supplementary Fig. 19, 20), with a single exception of the *H. clysonymus* + *H. hortense* + *H. telesiphe* branch, which could be due to high rates of ILS or introgression (coalescent units = 0.08), disrupting the monophyly of the *Erato* group. We find little evidence of ILS around these basal nodes, with the percentage of quartets in gene trees that agree with the ML topology (normalized quartet support) q1 (f1) being 0.62 (1989); higher than nodes supporting other deep splits in *Heliconius* (Doris + Wallacei + Silvaniform + Melpomene clade, with the Wallacei + Silvaniform + Melpomene branch). A further assessment of nodal support was performed using the Quartet Concordance (QC), Quartet Differential (QD) scores, and Quartet Informativeness (QI) (within Quartet Sampling) to identify quartet-tree/species-tree discordance (see Methods). The position of *H. aoede* remained supported, with a strong majority of quartets supporting the focal branch (QC = 0.9), with a low skew in discordant frequencies (QD = 0) indicating that no alternative history is favoured, and no sign of introgression (i.e., QD < 1 but >0) and a QI of 1 indicates that the quartets passed the likelihood cut-off in 100% of the cases. This is also true for the Doris group, once placed outside *Heliconius* (Brown 1981), which show perfect scores (QC/QD/QI: 1/-/1) clustering sister to Wallacei + Silvaniform + Melpomene clades (supplementary Fig. 22, 23).

Second, as *Heliconius* are a key example of the impact of gene flow and hybridization on adaptive divergence[12,13], particularly with the advent on short paired-end read sequencing libraries[1,9,14], we revisited this topic adopting a different methodological approach on a wider taxonomical range, using two complementary phylogenomic approaches originally implemented in the *Drosophila* radiation[15]; the discordant-count test (DCT) and branch-length test (BLT). These approaches differ from previous methods applied to gene flow in *Heliconius*[1,9,14], both relying on discordance between the species and gene trees, rather than SNPs or genomic alignment. Briefly, DCT is based on the amount of gene trees (scOG) that are topologically discordant with the inferred species trees, and BLT compares the distribution of branch lengths

for discordant gene trees among rooted triplets of taxa[15]. This identified several introgression events within Heliconiini, primarily within the major clades (Fig. 2a, b; supplementary data 4, 5). Among the non-*Heliconius* species, a single event was identified between *A. v. vanillae* and *A. v. incarnata*, and two within the genus *Eueides*: one, between *E. aliphera* and the most recent common ancestor (MRCA) of *E. vibilia*, *E. lampeto* and *E. isabella*, and a second between *E. aliphera* and *E. isabella*. A greater number of introgression events are detected within *Heliconius*, specifically between the MRCAs of Erato + Sara/Sapho clade and the Doris + Wallacei + Silvaniform + Melpomene clade. Several introgression events seem to have happened within the Erato group, particularly compared to the Sara/Sapho groups, potentially reflecting a greater tendency for monandrous females[16] in the latter, where males locate pupae to mate with eclosing females (pupal mating)[17]. For *H. aoede* there is no evidence of introgression with other non-pollen feeding Heliconiini or with the Erato/Sara/Sapho clade, but we identify two low-support introgression events with the Silvaniform/Melpomene common ancestor (significant triplets 5/98; $\gamma = 0.13$), and another with the Doris ancestor (significant triplets 2/68; $\gamma = 0.13$). Finally, most introgression events were identified in the Silvaniform/Melpomene clade. Some of the events identified here were previously found in other studies, such as between *H. hecalesia* and the MRCA of *H. clysonymus*, *H. hortense* and *H. telesiphe*, or between the MRCA of the Doris and Silvaniform/Melpomene clades[1,18,19]. There are a few "ghost" introgressions, events that happened between taxa that did not overlap in time, in our current dating, but all have extremely low support (significant triplets ≤ 3%; $\gamma < 0.01$). The estimated fraction of introgressed genome mostly varies between 0.02 to 0.15, with a peak around 0.30, within the Erato group (range of average γ estimates = 0.023–0.323). Most introgression events occurred in a restricted time frame between 5 Mya to present (Fig. 2b, c), and no significant relationship was found between the midpoint estimate of the timing of introgression (Mya) and the estimated γ (Fig. 2b), indicating that the fraction of a genome that is introgressed within *Heliconius* does not depend on the timing when introgressions occurred.

a

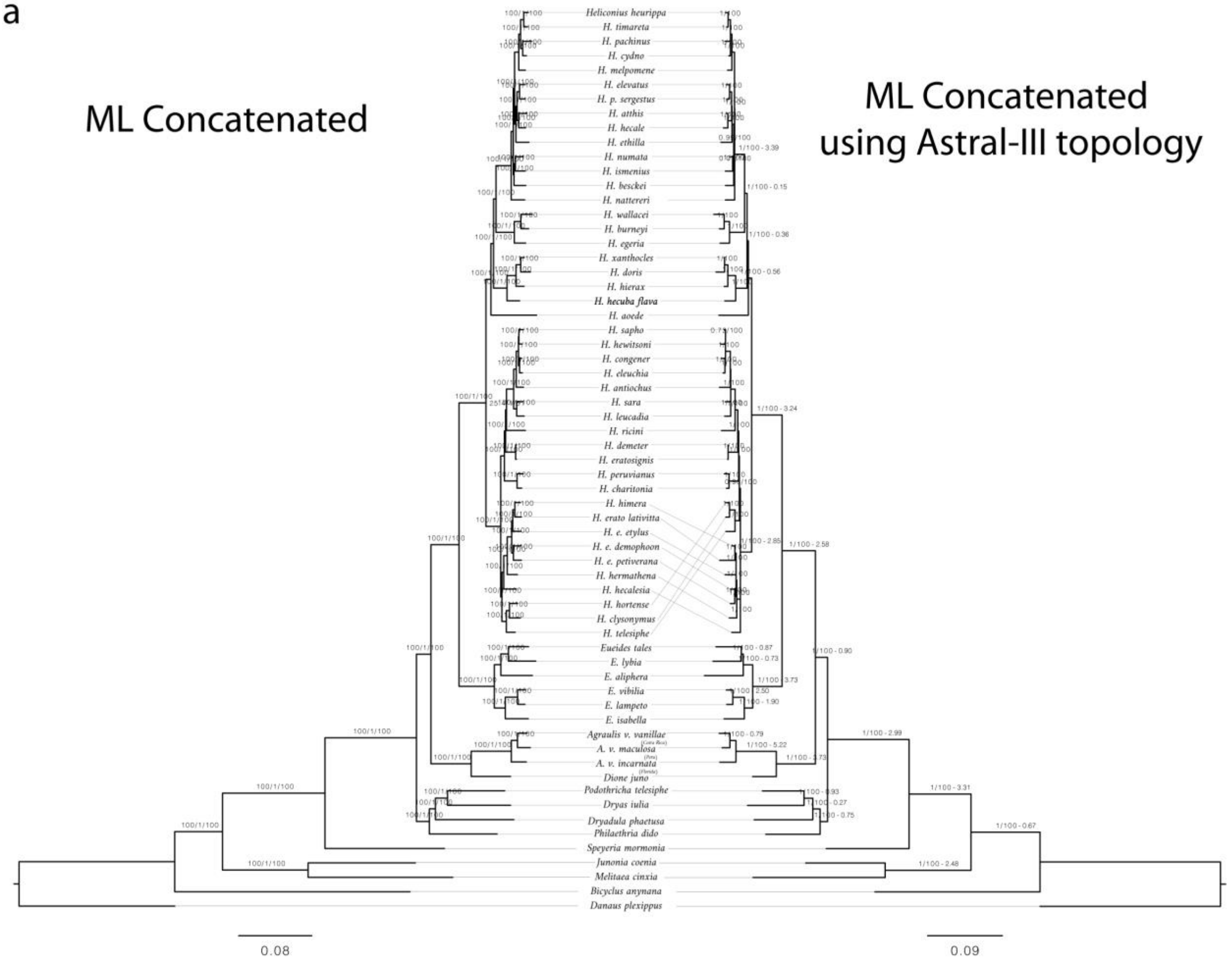

Supplementary Fig. 19 | *| Comparison of maximum likelihood (ML) concatenated species tree, and maximum likelihood (ML) concatenated dataset + (ASTRAL-III) species topologies. The number beside each node on the left tree indicates bootstrap support. On the right tree we used the concatenated dataset to infer branch length over the ASTRAL-III topology; bootstrap values plus the coalescent units (CU)/ gene concordance factor (GCF) shown to the right of the tree for Heliconius and more deeper nodes. The topological differences are minimal with a single displacement of H. hortense, H. clysonymus, and H. telesiphe monophyletic clade. The Alignment has 63 sequences with 4,011,390 columns, 2,273,960 distinct patterns, 1,558,772 parsimony-informative, 499,830 singleton sites, and 1,952,788 constant sites.*

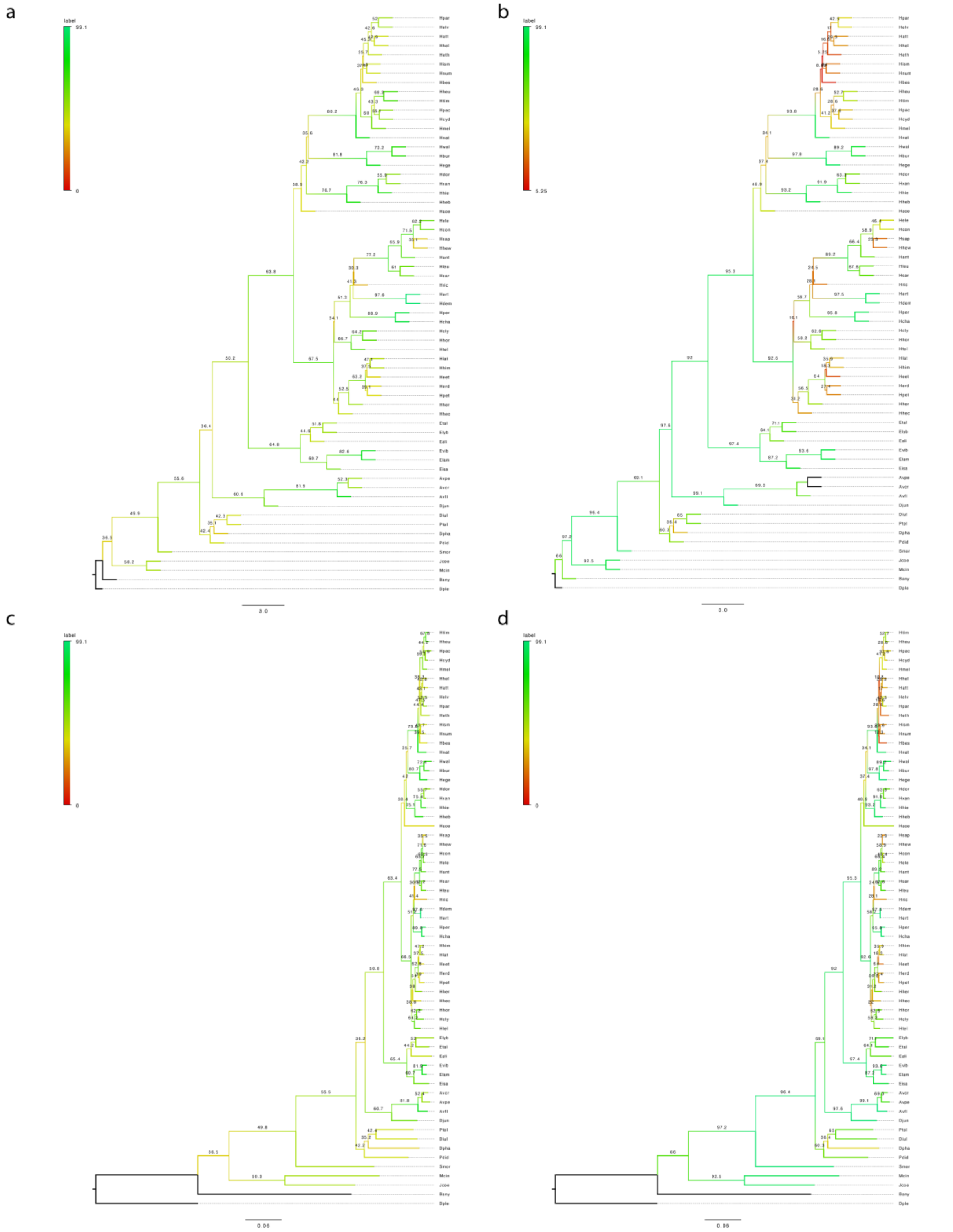

Supplementary Fig. 20 | IqTree concordance factor. *a) Astral III tree with site concordance factors (sCFs) computed with Iqtree2 at each node, using the concatenated alignment. b) Astral III tree with gene concordance factors (gCF) at each node using gene trees. c) Concatenated ML tree with site concordance factors (sCF) at each node using concatenated alignment. d) Concatenated ML tree with gene concordance factors (gCF) at each node using gene trees.*

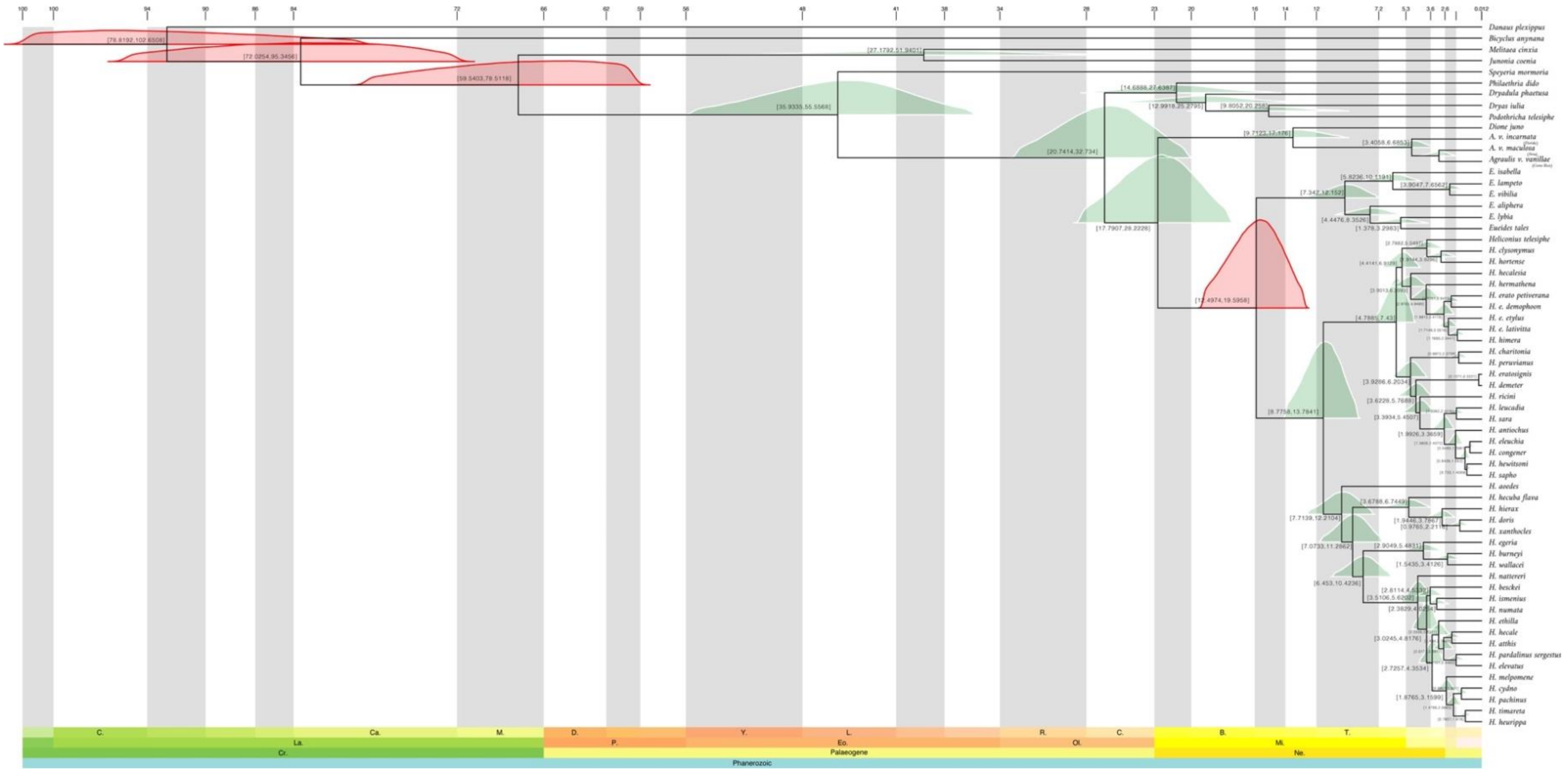

Supplementary Fig. 21 | Node calibrated maximum-likelihood phylogenetic tree*. Inferred dated tree of Heliconiinae from a supermatrix of 3,393 single-copy OGs (total of 4,011,390 sites). At each node the posterior probability distribution of divergence points from MCMCTree. The Red distributions correspond to calibrated nodes (supplementary data 3). At each node the 95% confidence interval is also present.*

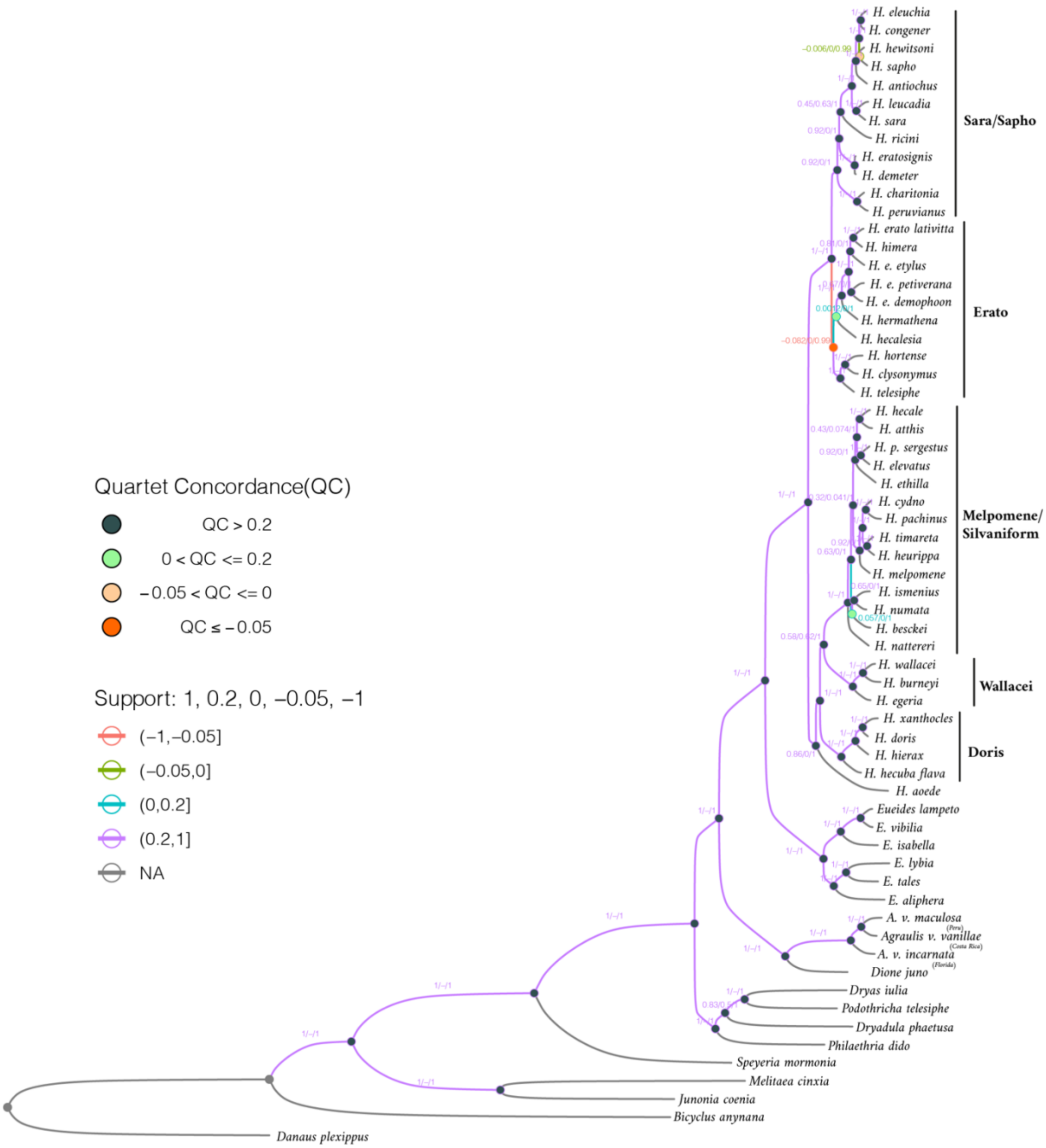

Supplementary Fig. 22 | Quartet Sampling analysis and conflicting supports in Heliconiinae ML species tree. *Phylogeny from ML analysis (supplementary Fig. 19,20). The branch colorations show the score support. In circles the Quartet Concordance (QC) scores for internal branches: dark green (QC > 0.2), light green (0.2 ≥ QC > 0), light orange (0 ≥ QC ≥ −0.05, or dark orange (QC < −0.05). Numbers at nodes indicate the QC, the Quartet Differential (QD), and the Quartet Informativeness (QI) scores (100 replicates of full alignment). The great majority of nodes have high scores with the exclusion of H. hortense, H. clysonymus and H. telesiphe clade, which is the clade that shows the topological conflict between the ML and ASTRAL tree.*

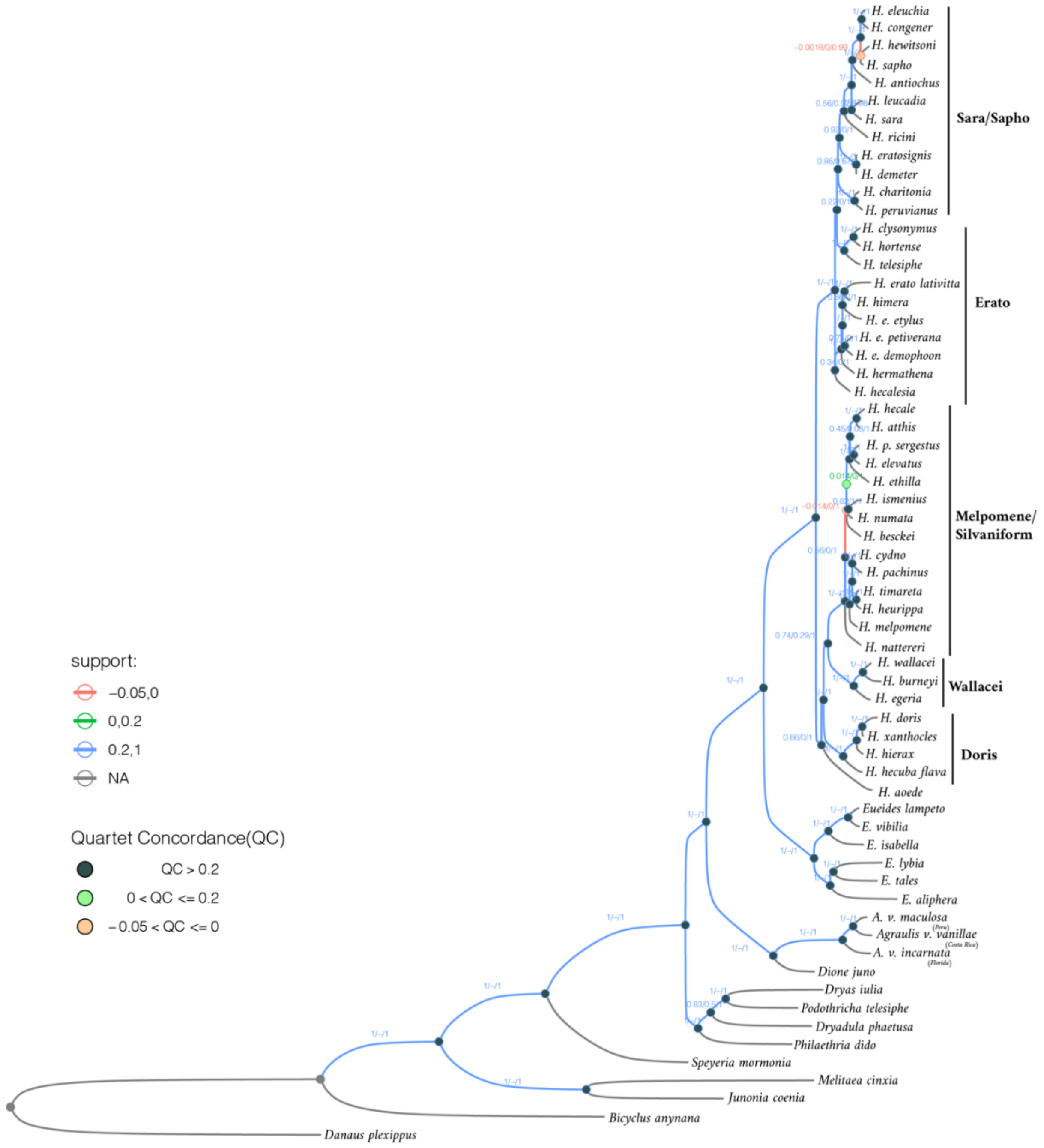

Supplementary Fig. 23 | Quartet Sampling analysis and conflicting supports in Heliconiinae ML search with ASTRAL-III topological species tree*. Phylogeny from ML analysis using the ASTRAL-III topology (supplementary Fig. 19,20). The branch colorations show the score support. In circles the Quartet Concordance (QC) scores for internal branches: dark green (QC > 0.2), light green (0.2 ≥ QC > 0), light orange (0 ≥ QC ≥ −0.05, or dark orange (QC < −0.05). Numbers at nodes indicate the QC, the Quartet Differential (QD), and the Quartet Informativeness (QI) scores (100 replicates of full alignment). The great majority of nodes have high scores, now including the exclusion of H. hortense, H. clysonymus and H. telesiphe clade, which now breaks the monophyly of the Erato clade.*

<u>Supplementary Note 3. Genomic landscape of topology, introgression, and ILS</u>

Pollen-feeding trait is one of the most important innovations within *Heliconius* radiation. The presence of *H. aoede*, a non-pollen feeder, formerly placed outside *Heliconius* genus, offers the possibility to understand the genetic basis of this trait and the conundrum about its emergence: whether it emerged once and never evolved if the species is outside pollen-feeding *Heliconius*, secondarily lost, or evolved independently twice with no loss, if the species is evolved from the common ancestor of all *Heliconius* species. So far, all the studies involving molecular data of this enigmatic species placed it within *Heliconius*. Using extensive genomic data in the form of scOGs, our data support the monophyletic status of the pollen-feeding *Heliconius* + *H. aoede*. Specifically, the clade seems to cluster sister to the stem of three other clades: Melpomene/Silvaniform, Wallacei and Doris (supplementary Fig. 24a). Leveraging the 63-way whole genome alignment and using as reference *E. isabella* we further tested the robustness of this topology by inferring the local topology history across the 63 species. Using a non-overlapping windows of 10kb we inferred ML trees and explored the frequency of different possible topologies, the effect of introgression and ILS with a coalescent based method. From the more 43 thousand non-overlapping sliding windows ~30 thousand return one of five main topologies, ~12 thousands no valid topology and ~400 did not contain sequences from *H. aoede*. Most of the windows (~70% on average) overlap with CDS, expected giving the presence of relatively short intergenic regions (median: ~3kb; mean: ~10k). We classified the five most frequent topologies based on the position of *H. aoede* relative to the other *Heliconius* clades, *Eueides* and other non-*Heliconius* species (supplementary Fig. 24a). The most frequent topology (Topology 1), supported by 49% of the genome, shows the same relationships of the species tree generated wit scOGs, where *Eueides* are sister to *Heliconius* and the Sara-Sapho + Erato clades are sister to *H. aoede*, sister to Doris/Wallacei/Silvaniform/Melpomene clades. The second most frequent topology (Topology 2; 12.8% of the genome), shows *H. aoede* nested in Doris/Wallacei/Silvaniform/Melpomene clades. The third (Topology 3; 3.7%) and forth topologies (Topology 4; 1%) show instead *H. aoede* sister to all the other *Heliconius*, and nested in Sara-Sapho + Erato clades, respectively. In total over 65% of trees support *H. aoede* within *Heliconius* and only the 3.7% support being sister to all the other *Heliconius* species. Topology 1 shows the highest number of consecutive window topologies with block lengths reaching over 100kb more than 200 times (supplementary Fig. 24b). That is followed by topology 2 with two blocks longer than 100kb, while other topologies are represented by shorter blocks.

Because the Z chromosome was previously reported to be less affected by introgressions [9], we estimated the fraction of the genome that introgressed (average *f*-branch statistic) across all triplet comparisons from each chromosome and Z chromosome versus all autosomes (supplementary Fig. 25). Indeed, the Z chromosome does show a lesser degree of introgression overall, but without dramatically changing the topology distributions. Topology 1 remains the most frequent (56%) followed by topology 2 (7.1%), topology 3 (5.2%). The introgression patterns, with the exception of some changes within the Erato clade, and *H. clysonymus* + *H. hortense* with the base of Sara/Sapho clade (supplementary Fig. 25), are also largely unaffected. In fact, the highest frequency of each topology is 65.8% in chr3 (*Heliconius* chromosome 11), 20.5% in chr24 (fused with chr14 in the *Heliconius* chr18), and 9.3% in chr6 (fused with chr22 in the *Heliconius* chr10), respectively for Topology 1, 2 and 3. We also explored concordant factor (CF) across chromosomes (supplementary Fig. 26). The lower introgression in the Z chromosome is evident in the higher values of the CF in clades affected by a higher degree of gene flow (*e.g.*, Melpomene/Silvaniform clade), but overall, the CF for the Z chromosome is within the range of the other chromosomes, except for the Sara-Sapho and Wallacei/Melpomene/Silvaniform clades, which clearly appears as an outlier (supplementary Fig. 26b).

Overall, the landscape of local history seems to confirm the species tree as the most consistent topology, with *H. aoede* clustering within *Heliconius* clades. This would exclude the most parsimonious option of only one gain event for the pollen-feeding, confirming that two events, equally parsimonious remains: one gain at the stem of Heliconius clade, followed by one loss at the branch of *H. aoede*, or two independent gains at the base of Sara-Sapho/Erato and Doris/Wallacei/Melpomene/Silvaniform.

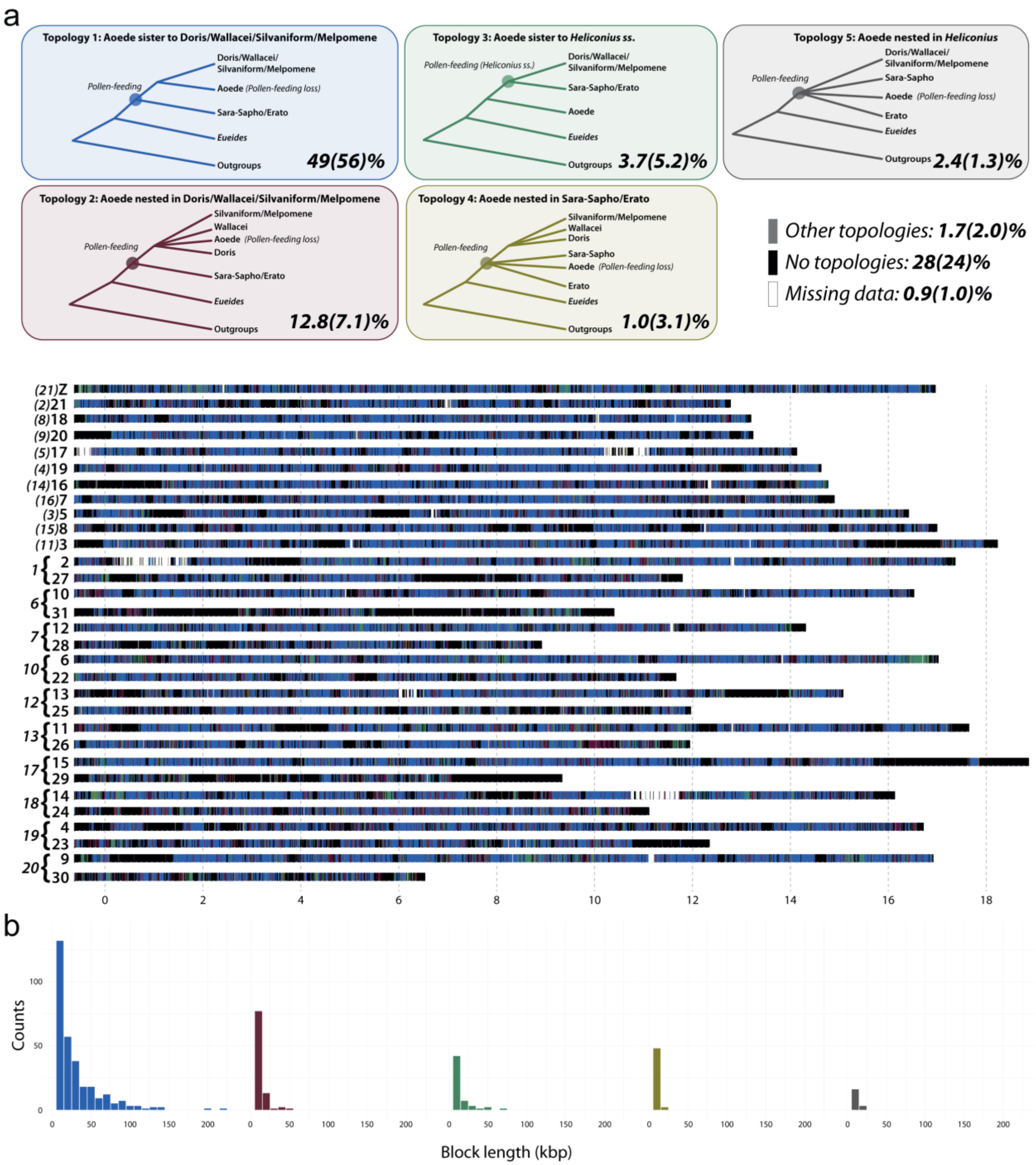

Supplementary Fig. 24 | Phylogenetic landscape. A *Phylogenetic landscape of the most common topologies that focus on the split at the base of Heliconius species. Numbers in the rectangles indicate the proportion of the genome/sliding windows that produce the given topology across the entire genome; in parenthesis the proportion is relative to the Z chromosome only. Below the topologies diagrams showing for each ancestral chromosomes 10 kb sliding windows, in colours the corresponding topology. Numbers in parenthesis are the corresponding chromosome in Heliconius, while the one next to the curly brackets the corresponding fused chromosomes in Heliconius.* ***b*** *Distribution of the sum of continuous topological blocks. Topology 1 (blue) shows the longest continuous blocks of 100kb and more.*

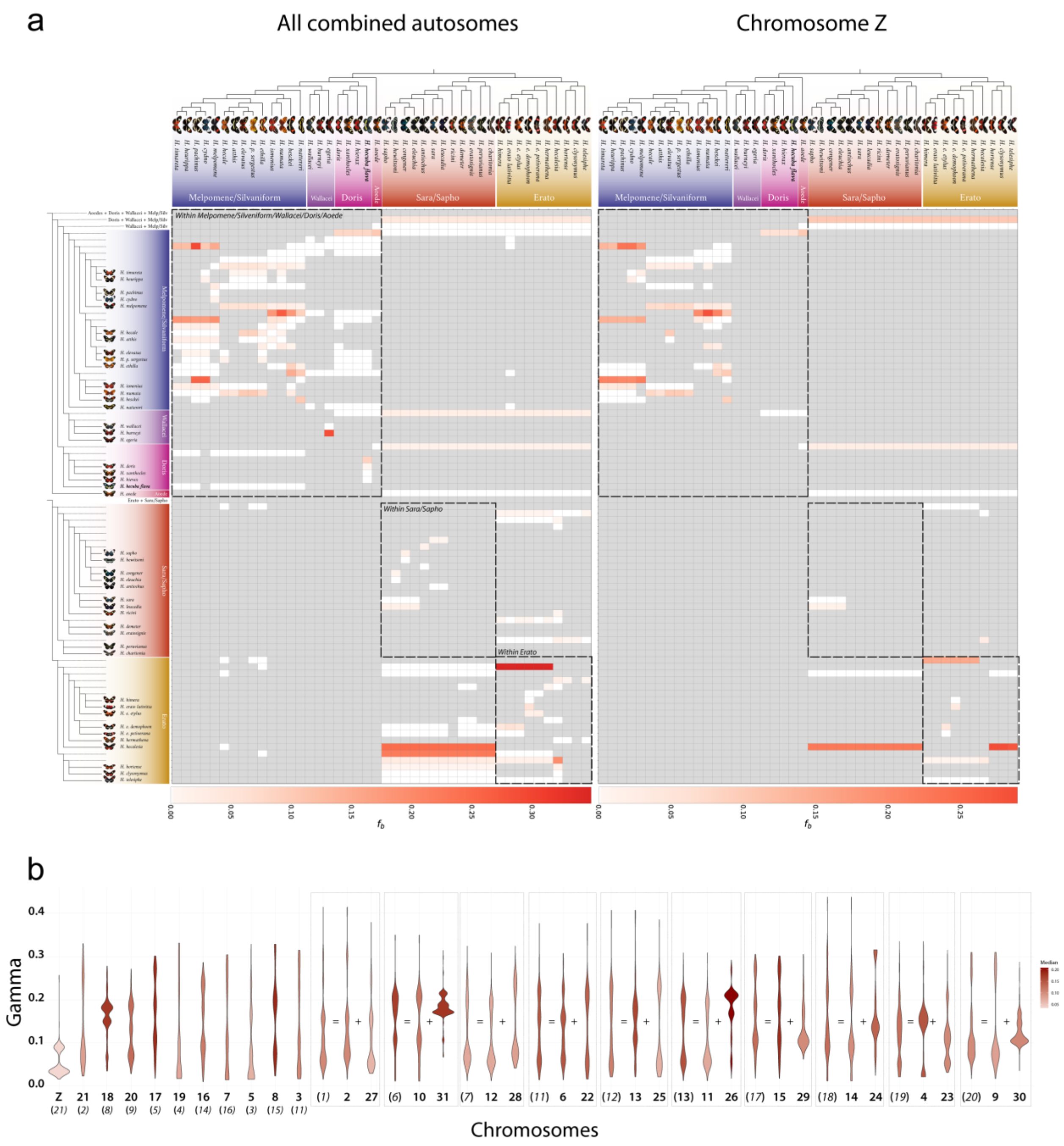

Supplementary Fig. 25 | Landscape of introgression. A *Matrixes showing the inferred introgression proportions as estimated from the sliding windows, from all combined autosomes (left) and Z chromosome (right), in the introgressed species pairs, and then mapped to internal branches using the f-branch method. The expanded tree at the side of the matrix shows both the terminal and ancestral branches.* ***B*** *Violin plots showing the inferred gamma, the proportion of genomic introgression, from each chromosome. Numbers in parenthesis are the corresponding chromosome in Heliconius, while the dashed rectangles indicate the ancestral chromosomes that are fused in Heliconius.*

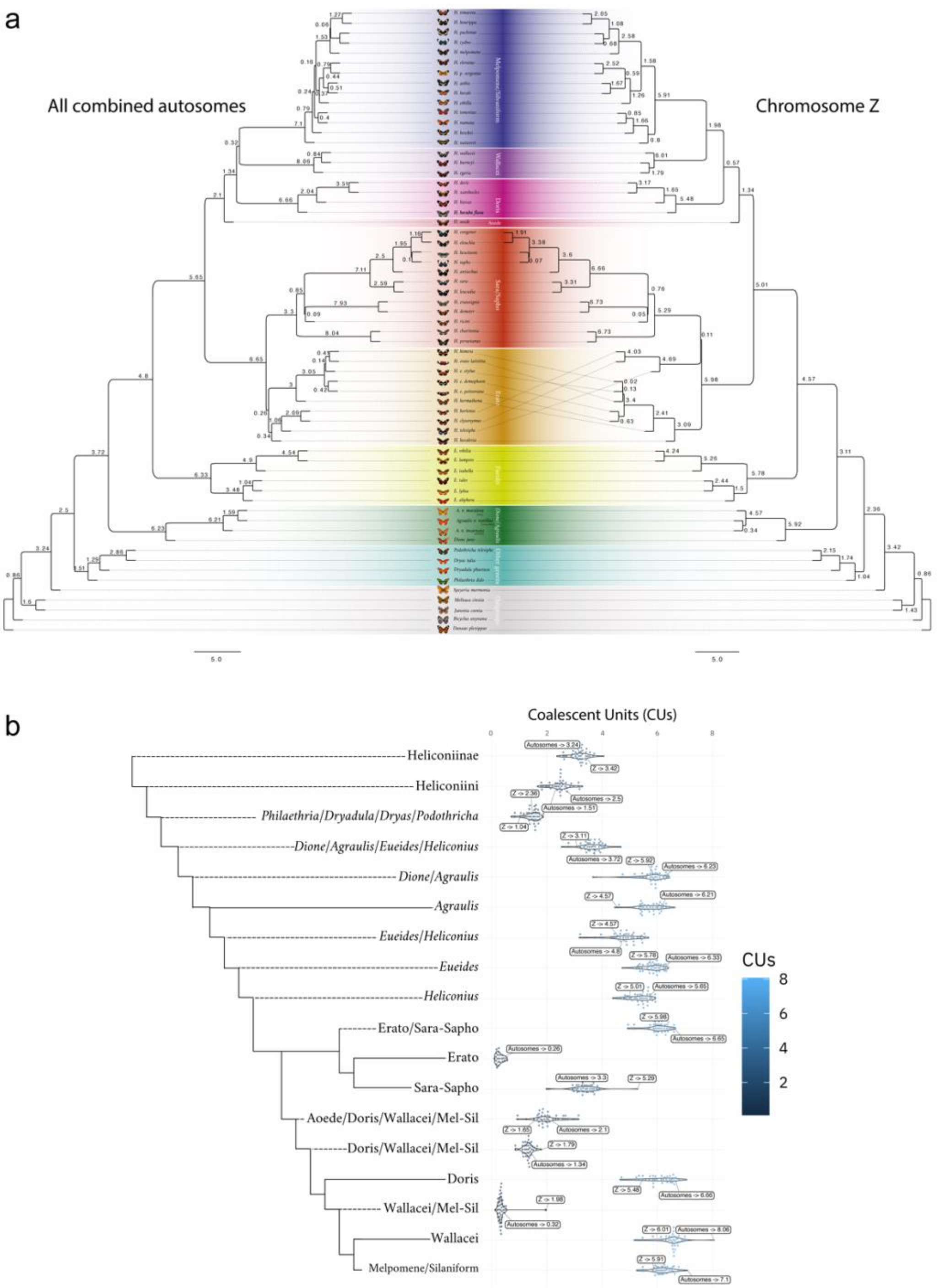

Supplementary Fig. 26 | Landscape of concordance factors. A *ASTRAL-III species trees inferred from the 10 kb sliding windows, from all combined autosomes (left) and Z chromosome (right). Numbers at each node indicate the relative branch length, which correspond to the coalescent units (CUs).* ***b*** *Violin plot showing the distribution of CUs at each node for all ancestral chromosomes, including the CU from all autosomes.*

Supplementary Note 4. Evolution of Genome Size and Content

Genome size variation has been formalised in an "accordion" model[20], in which genome size can gain, lose, or maintain its size in equilibrium in each species, with increases in size due to TEs balanced by decreases due to large segmental deletions. This pattern has been documented in several taxa, such as squamates[21], birds, mammals[20], and *Drosophila*[22]. Our extensive comparative genomic study, as well as the knowledge on the temporal and phylogenetic relationships of the tribe Heliconiini, allowed us to analyse the evolutionary dynamics of genome size across this whole tribe. The inferred genome size ancestral state reconstruction indicates that the Heliconiinae genome size was not constant over time. On the contrary, there was a trend toward a genome reduction (Fig. 3a, c): the MRCA of Heliconiinae had a genome size of ~400M and all internal branches leading to the Silvaniform + Melpomene branch (~283Mb) had a constant reduction (*e.g.*, from ~367Mb for Heliconiini branch to ~308Mb for Wallacei + Silvaniform + Melpomene branch). There were also several independent expansions in branches leading to *Philaethria*, *Dryadula*, *Dryas* and *Podothricha* (~372Mb), and within the genus *Heliconius* in the Erato (~353Mb), Doris (~315Mb) and Wallacei (~329Mb) groups. Strikingly, *H. aoede* shows an estimated loss of about 68 Mbp, a fifth of its genome size (~22%) from its ancestral node.

Different genomic compartments (CDS, introns, 5′-UTR, and 3′-UTR) typically evolve under different degrees of selection pressure. To confirm this in our data, and to test for consistent patterns across the two most speciose clades, *Heliconius* and *Eueides*, rates of evolutionary change of different genomic compartments were computed by calculating CONACC scores from PHYLOP, assessing departures from neutrality across all sub-branches of the *Heliconius* and *Eueides* taken together (Fig. 3b). CONACC scores showed marked differences among genomic features, with CDS sequences being the most conserved feature, followed by 5′-UTRs, 3′-UTRs, and finally by introns, consistent with expectations that coding regions are more constrained compared to non-coding, and that sites in 5′-UTRs will be enriched for high scores compared with 3′-UTRs, as observed in vertebrates[23]. Between the two genera, we identified an enrichment for higher CONACC scores in *Heliconius* for CDS and introns, compared to the same compartments in *Eueides*. A trend that is inverted for the two UTR regions (Wilcoxon rank-sum test 'two-sides' P value < 2.2×10^{-16}). This suggests an increased tendency for clade-specific selection. We tested whether the shift towards higher scores in the CDS of *Heliconius* was driven by an increase of negative purifying selection over positive selection by inferring the number of sites under purifying and positive selection separately in *Heliconius* and *Eueides.* The adopted fast-unconstrained Bayesian approximation (FUBAR) [24] showed that the number of sites under purifying selection is several orders of magnitude higher

than the sites under positive selection (supplementary Fig. 33), with *Heliconius* having 2.5 times more sites under purifying selection per codon than *Eueides*, suggesting the CONACC enrichment in *Heliconius* might be more likely due to higher degrees of purifying selection.

The distribution of intron length is one of the least consistent features of genome structure (supplementary Fig. 12-15) and one of the main contributors to genome size (supplementary Fig. 9) in our dataset. Heliconiini species with longer introns tend to have larger genomes (supplementary Fig. 18a), with a relatively high correlation with the total TE content (supplementary Fig. 34; Pearson's ρ=0.72; R^2=0.51). On average, introns have a median length of 257b ± 40, with the smallest median (200 b) in *H. pachinus*, for which 19.1% of its genome is made up of TEs, and the highest median (345 b) in *Dy. Iulia,* in which 31.7% of its genome is constituted by TEs. The average intron length across Heliconiinae is 306 ± 104 kb, with the smallest average being 146 kb in *H. cydno* (22.7% TEs); and the longest 754 kb in *H. timareta* (23.5% TEs) (supplementary data 1). To explore these very skewed distributions, introns of each species were classified as 'short' or 'long' based on the median values of the species [5] to explore how they differ in TE content. Long introns contain significantly more TEs than short introns (supplementary Fig. 34; Wilcoxon rank-sum test *P* value = 2.13×10^{-13}), and significantly higher amount than would be predicted by their length (*P* value = 1.3×10^{-10}; elevation of long introns 1.11 vs. shorts 0.84, slopes *P* value > 0.05; Fig. 4a). As a consequence, longer introns are significantly more GC rich (Fig. 4b). Genome compaction, measured as gene density (number of loci/genome size), shows relatively low diversity among species, with an average of 62±15 genes per Mb. Interestingly, TE content correlates less with genome compaction less (supplementary Fig. 31; Pearson's ρ=-0.6; R2=0.36) than with intron size (supplementary Fig. 34; Pearson's ρ=0.72; R2=0.51). This suggests that Tes are affecting gene length more than intergenic regions, as has been observed in other organisms[25,26]. These results, together with evidence that TEs accumulate more in the tails of the chromosomes in Heliconiini[5], suggest that intergenic regions might be under purifying selection, perhaps to avoid the disruption of regulatory elements[27].

Intron–exon boundaries were also explored using scOGs as a proxy for all protein-coding genes within Heliconiinae. Two analyses were performed, one looking at intron gain and loss throughout the phylogeny and the percentage of intron retention from the MRCA of Nymphalids. Both analyses show a relatively stable dynamic over the last 50 Mya for Heliconiinae. The evolutionary histories of intron gains and losses revealed no significant shift among species, with ~7% of ancestral intron sites retained across species (Fig. 4c), which is similar to patterns reported in a phylogenomic analysis of *Bombus* species (Hymenoptera)[28]. Nevertheless, this result differs from drosophilids and anophelines, which show

significantly more intron losses than gains [29], and from *Dinophilus gyrociliatus* (Dinophilidae: Annelida), in which a dramatic loss of introns was followed by an intense genome size reduction[26].

To gain a more detailed understanding of the putative mutational events that accountable for the genomic changes that balance TE content evolution, different mutation spectra were extracted from the whole-genome alignment (supplementary Fig. 27-32). Insertions are the most frequent and abundant of mutation events, supporting the dynamic accumulation of TEs, while inversions and transpositions are the least abundant, also in line with the conserved synteny between these butterflies[5]. By calculating the mutated Mbps per Mya (rate), the rate of insertions is estimated to have been high across Heliconiini, particularly at the base of the Erato group, and the MCRA of Doris + Wallacei + Silvaniform + Melpomene, with a dramatic reduction within Silvaniform/Melpomene species (supplementary Fig. 34). By comparing deletion and duplication rates at each node, all internal branches of Heliconiini show values > 1, meaning that deletion events are more prevalent than duplications, with independent inversions of this trend leading to Sara-Sapho + Erato, Erato, Doris and Wallacei clade MRCAs (Fig. 4c). Together these data are consistent with the "accordion" hypothesis, with TEs insertions being balanced by loss of genomic content.

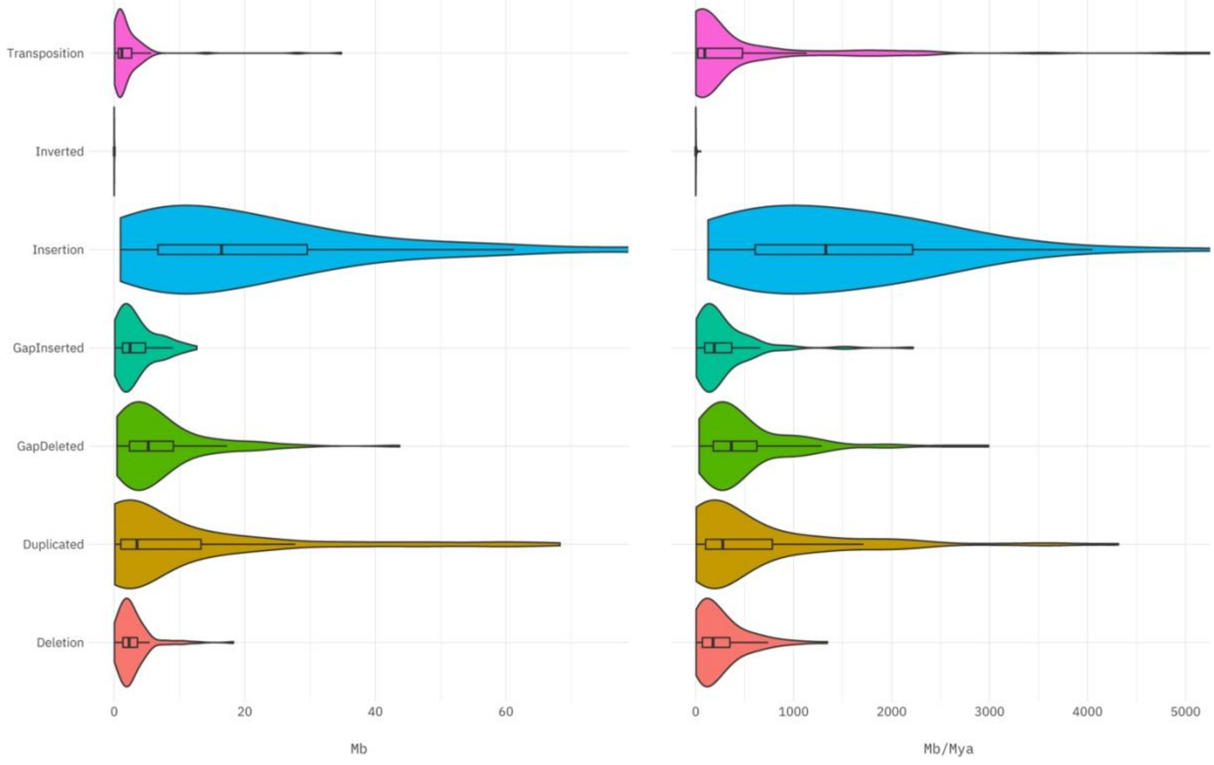

Supplementary Fig. 27 | Genome rearrangements. *Distributions of different types of structural genomic variation across Heliconiinae. Within the violin plots boxplots where bars indicate the interquartile ranges (IQR), while whiskers the quartile (Q) ± 1.5*IQR.*

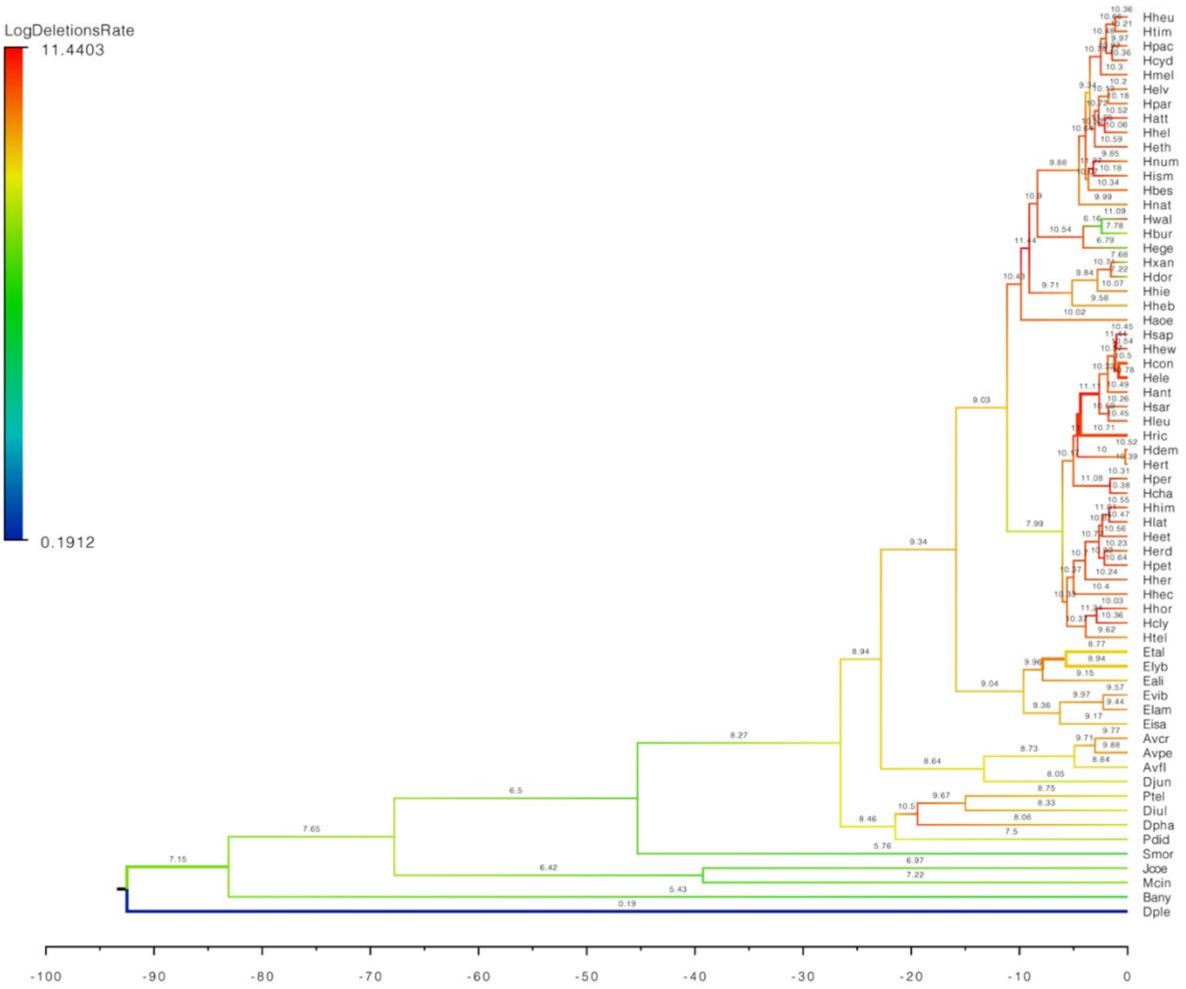

Supplementary Fig. 28 | Genome wide deletion rate. *Map of the deletion rates of genomic regions across the Nymphalid phylogeny.*

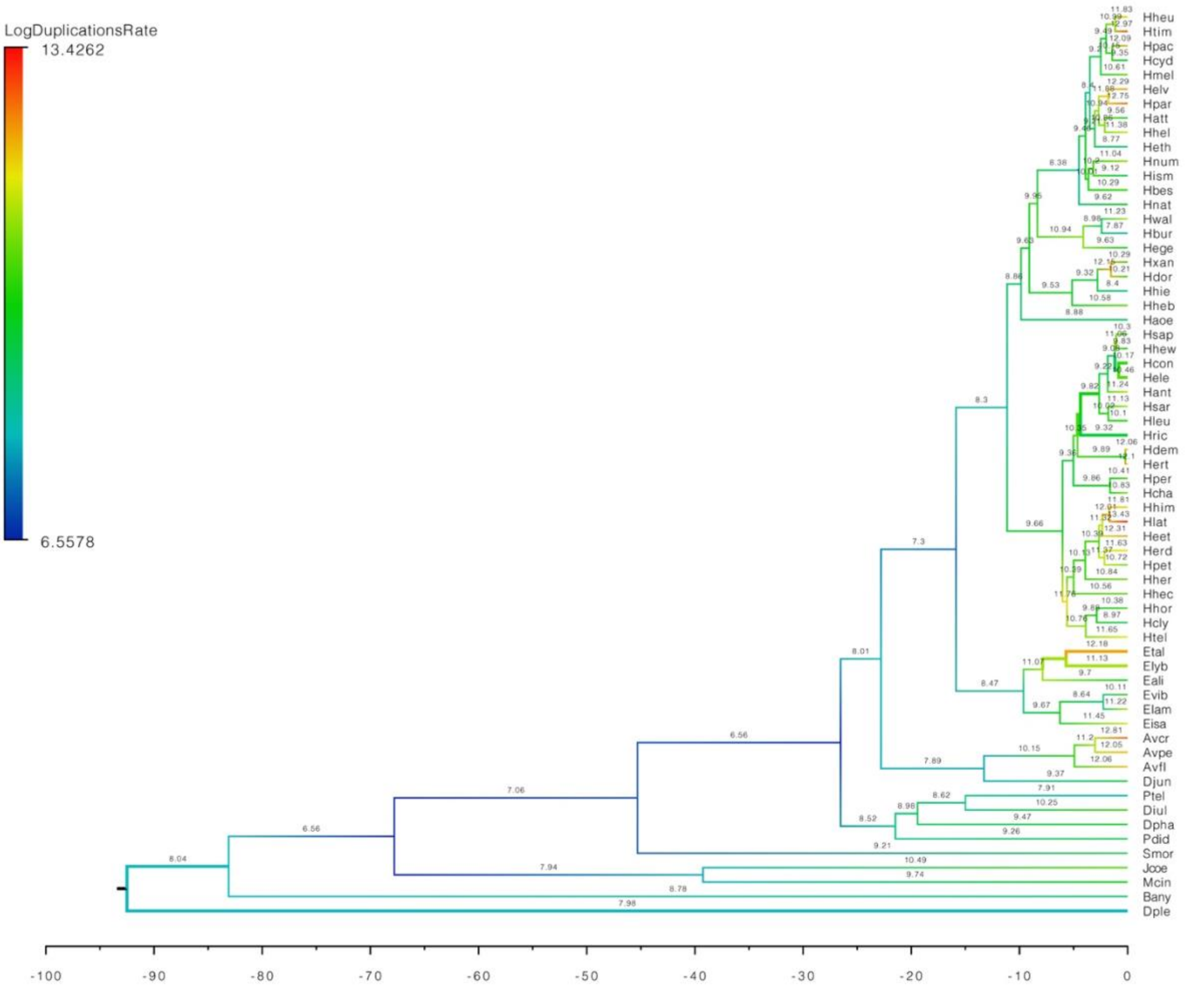

Supplementary Fig. 29 | Genome wide duplication rate. *Map of the duplication rates of genomic regions across the Nymphalid phylogeny.*

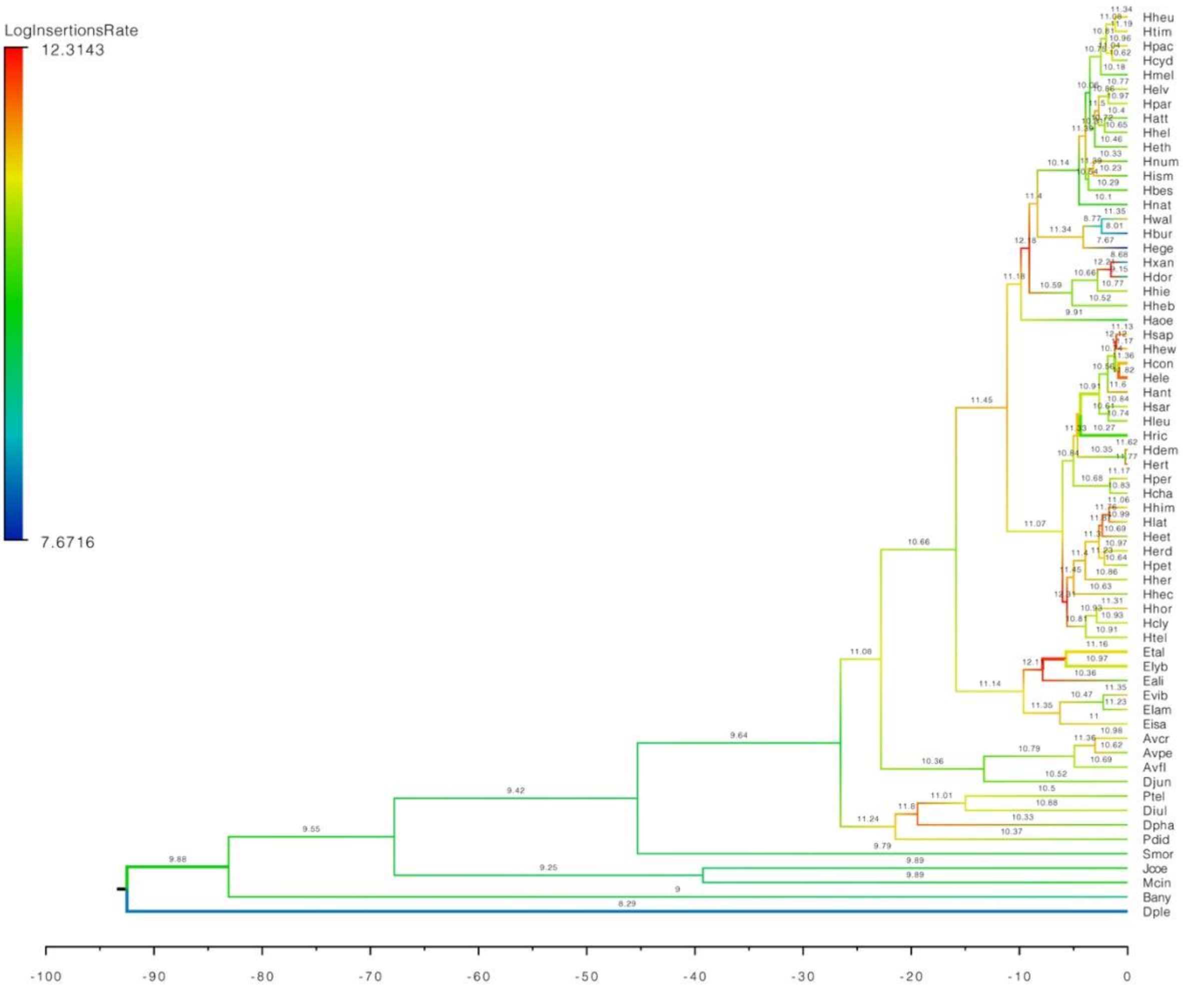

Supplementary Fig. 30 | Genome wide insertion rate. *Map of the insertion rates of genomic regions across the Nymphalid phylogeny.*

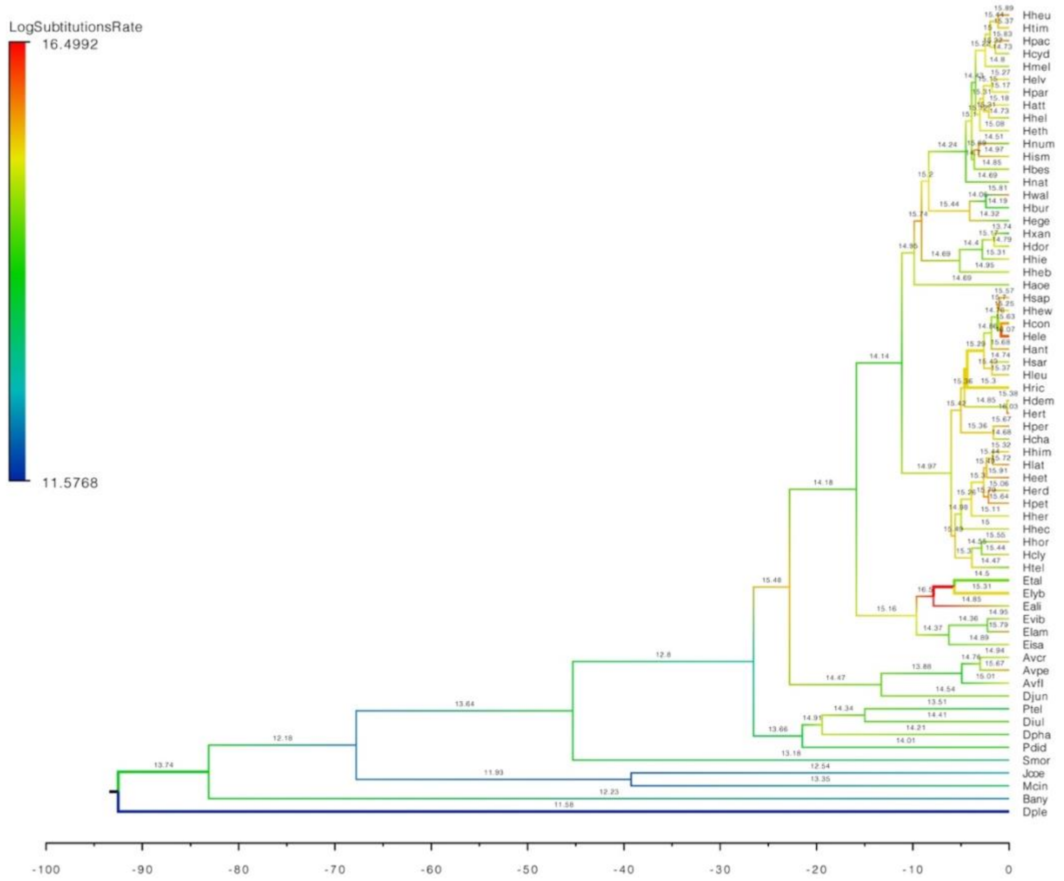

Supplementary Fig. 31 | Genome wide substitution rate. *Map of the substitution rates of genomic regions across the Nymphalid phylogeny.*

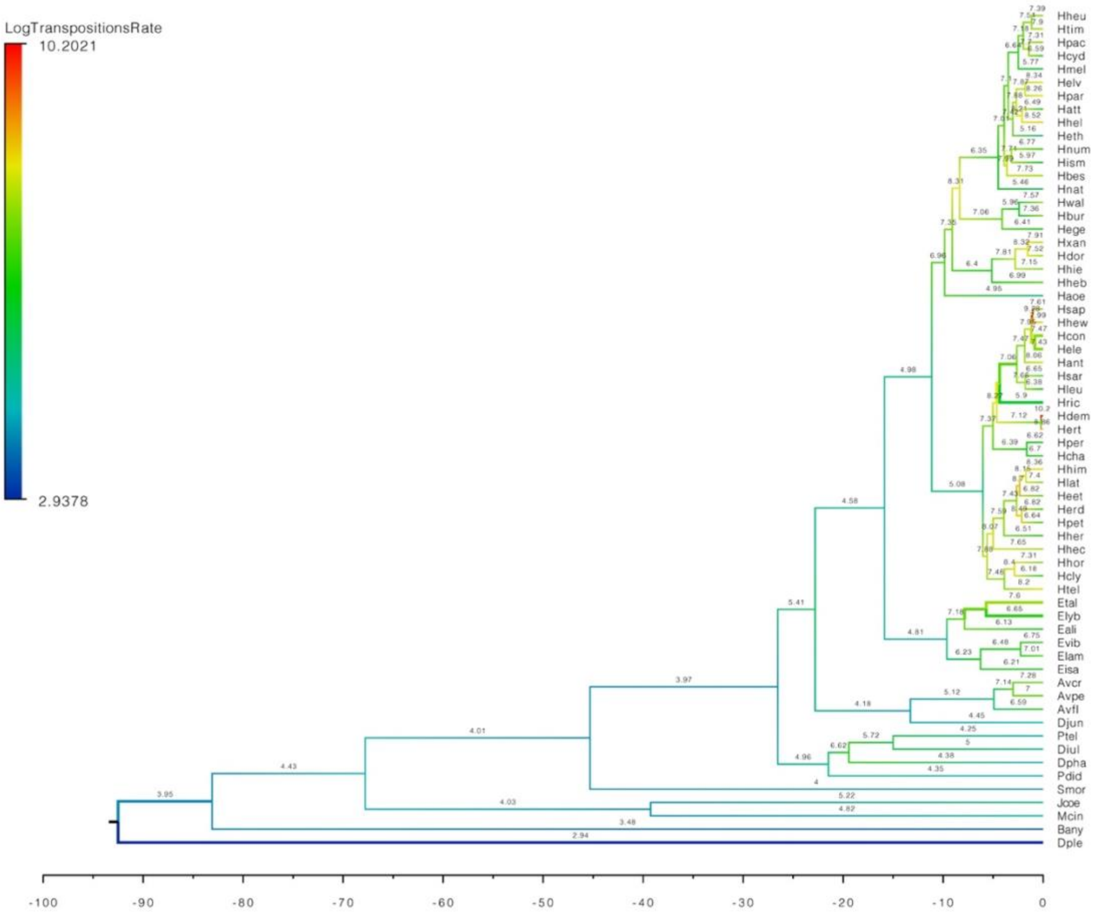

Supplementary Fig. 32 | Genome wide transposition rate. *Map of the transposition rates of genomic regions across the Nymphalid phylogeny.*

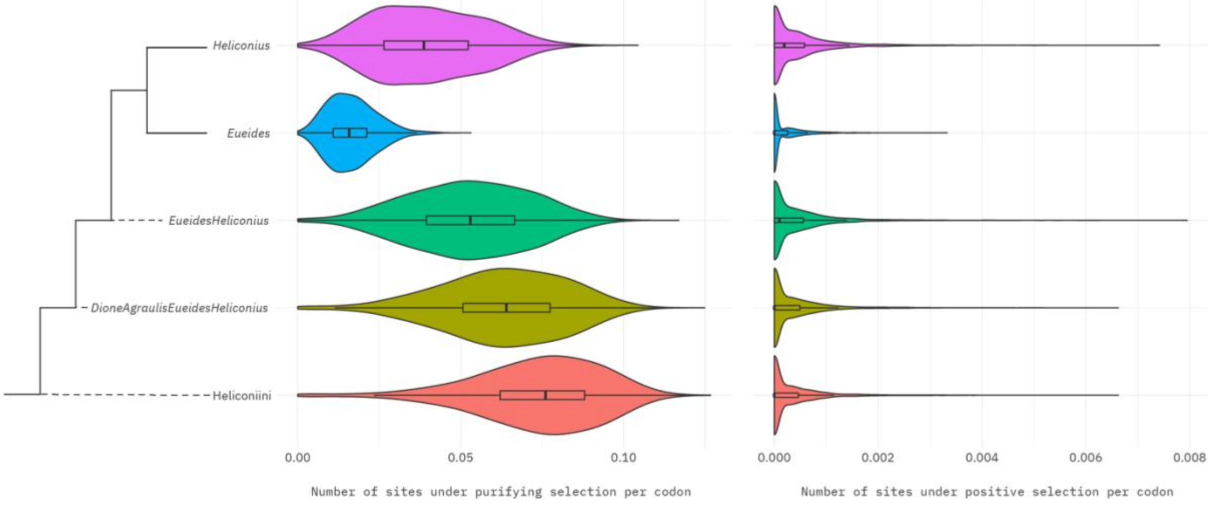

*__Supplementary Fig. 33 | FUBAR diversifying selection analysis__. Violin plots showing the number of sites under purifying selection (left) and positive selection (right) per codon. Within the violin plots boxplots where bars indicate the interquartile ranges (IQR), while whiskers the quartile (Q) ± 1.5*IQR.*

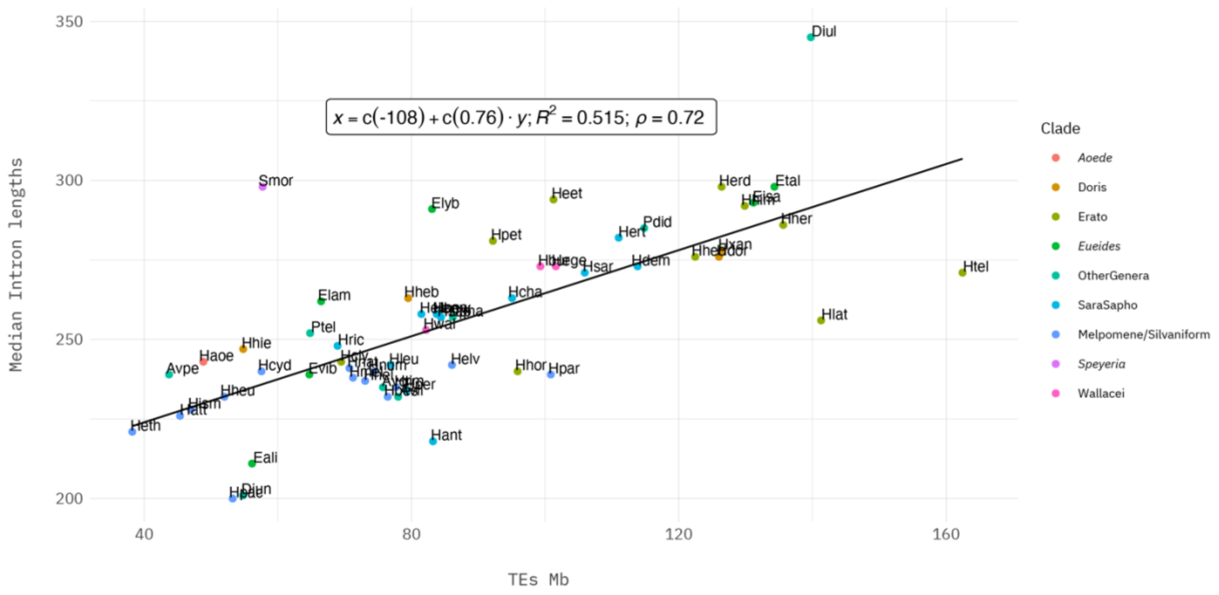

__Supplementary Fig. 34 | Median intron size vs Transposable Elements__. Correlation between median values of intron size per species and TEs indicate a high correlation between the two within Heliconiini (Pearson's ρ=0.72; R^2=0.51). The correlation was also corrected for phylogenetic signal suggesting that intron length is associated with assembly size independent of phylogenetic effects (F-statistic = 24.65 on 1 and 61 DF; P-value = 5.853e-06; r^2 = 0.2761).

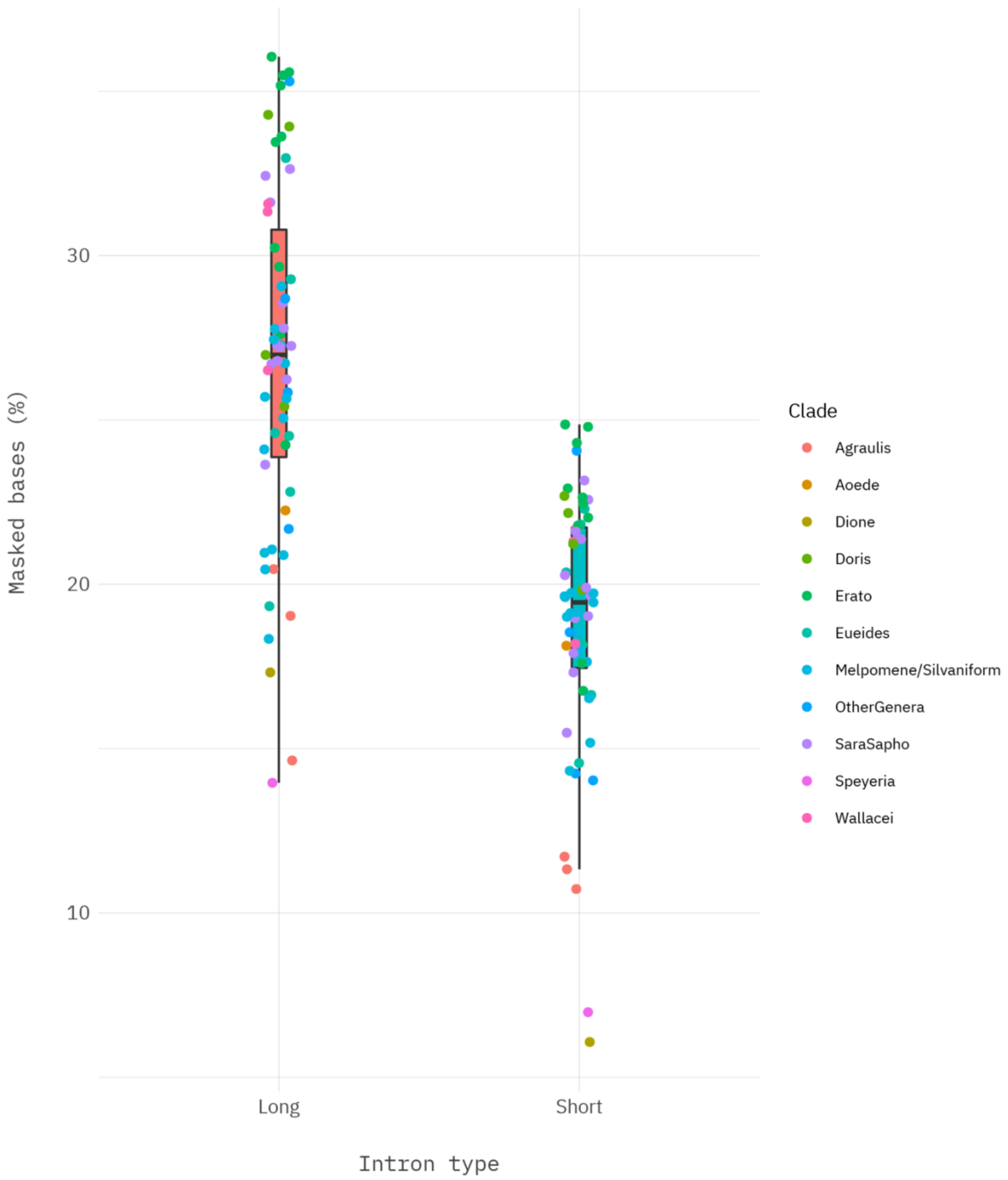

Supplementary Fig. 35 | Composition of intronic regions. *Intron types have a significantly differ amount of TEs; with long introns being more TE rich (One-sided Wilcoxon rank-sum test P value = 2.13x10*$^{-13}$*). Boxplots where bars indicate the interquartile ranges (IQR), while whiskers the quartile (Q) ± 1.5*IQR.*

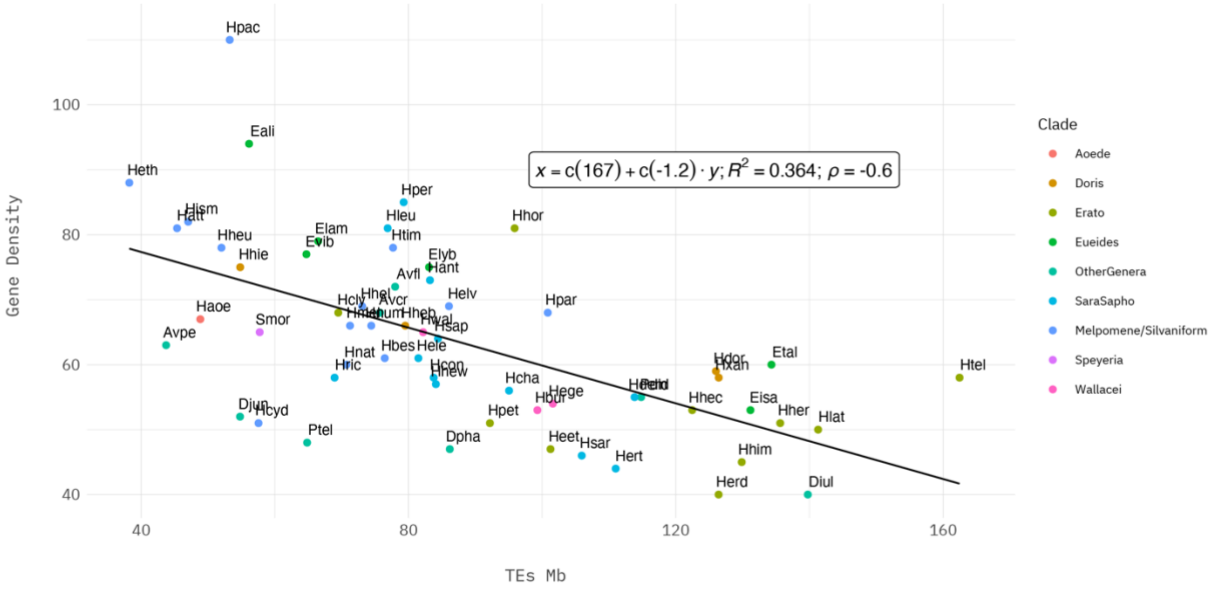

Supplementary Fig. 36 | Gene density vs Transposable Elements. *Correlation between median values of intron size per species and Tes indicate a high correlation between the two within Heliconiini (Pearson's ρ=-0.6; R^2=0.36).*

<u>Supplementary Note 5. Expansion and Contraction of Gene Content</u>

To explore how, and if, the genomic changes described above influence gene content, ortholog group (OG) size evolution was modelled with CAFE for 10,361 OGs using the 52 most complete genomes (BUSCO score ≥ 90%), to account for potential biases of incomplete assemblies and annotations (see Methods; supplementary Fig. 37). The analysis identified 656 OGs which vary in size across species (*P*-value < 0.05), with an estimated overall rate over 10 runs (supplementary data 6) of gene turnover (λ) of 0.006/gene gain-loss/Mya, which is relatively high compared to rates for *Bombus* (λ = 0.004) and anopheline species (λ = 0.003)[28,29], but similar to drosophilids (λ = 0.006)[30]. At the base of the phylogeny (*i.e.*, Nymphalinae + Heliconiinae, Heliconiinae and Heliconiini MCRAs) there was relatively strong OG expansions (branches leading to *Dione* + *Agraulis* + *Eueides* + *Heliconius* and *Eueides* + *Heliconius* MRCAs), with few contractions, followed by stasis. While the MRCAs of *Dione* + *Agraulis* and *Eueides* have a similar proportion of expanded and contracted OGs, *Heliconius* have 48 contracted OGs versus only eight expanded OGs (Fig. 3c, supplementary data 7). Contractions are also frequent in the Aoede, Doris, Wallacei, Silvaniform, Melpomene clades, while for both Sara/Sapho and Erato clades, several OGs were expanded, a trend that generally reflects the deletion/duplication rates for the different clades.

Notably, several OGs were expanded multiple times across the tribe. Regarding their PFAM domains, genes with the Cytochrome P450 domain (PF00067) expanded in the common ancestor of the subfamily Heliconiinae, the tribe Heliconiini, the *Dione* + *Agraulis* branch, and within the genus *Heliconius*

in the Erato group and Silvaniform/Melpomene branch. In insects, Cytochrome P450s are related to the detoxification of specialized metabolites and hormone biosynthesis/signalling. Cytochrome P450s are also involved in the biosynthesis of cyanogenic glucosides in heliconiine butterflies, which form the basis of their chemical defence[31–33]. Notably, a range of diet related OGs are also highlighted: Glucose transporters (PF00083) expanded several times in both Heliconiinae and Heliconiini, Trypsins (PF00089) expanded in Heliconiinae, Heliconiini, *Eueides*, and the Silvaniform/Melpomene branch, and finally Lipases (PF00151) expanded in the Sara/Sapho + Erato branch and Silvaniform/Melpomene clades. Although glucose transporters play an important role in energetic metabolism in all animal species, in phytophagous insects they are also hypothesised to be involved in the sequestration and detoxification of specialized metabolites from plants[34]. Lipases are key enzymes in energetic metabolism, but also have roles in non-dietary functions such as pheromone biosynthesis[35]. These results therefore suggest potential links to the chemical ecology of the Heliconiini. Among contracted OGs the most frequently conserved domains have known associations with TEs, such as the PiggyBac transposon system (PF13843) or Reverse transcriptase (PF00078). Among the OGs expanded on the *Heliconius* stem branch, where a novel suite of traits are thought to have evolved, our analysis highlights *methuselah*-like (*mth*), a G-protein coupled receptor, which is involved in oxidative stress response, metabolic regulation, and lifespan regulation in *Drosophila*[36,37], and *Esterase P* (*Est-P*), which expanded twice, and a *juvenile hormone acid methyltransferase* (*jhamt*), which is expanded three times, and is required for catalysis in the final reaction in which the carboxyl group juvenile hormone acids and farnesoic acids are methylated, generating the corresponding active JH compounds[38].

The unsupervised analyses on evolutionary pressures and gene turnover of OGs showed several gene families possibly affected by selection and contraction/expansion. To explore this in more in detail, we examined 57 gene families that maximise multiple aspects of cell biology, including a combination of transporters (*e.g.,* ABC transporters, amino acid transporters), receptors (*e.g.,* Rhodopsin-like receptors), enzymes (*e.g.,* P450, Carboxylesterases, Methyltransferases), and developmental proteins (*e.g.,* Insect cuticle protein, Vitellogenin) (supplementary data 8). We assessed the degree of "phylogenetic instability"[39] in these gene families, which provides a measure of incongruence between the single gene phylogeny and the species tree caused by processes such as gene duplication/loss, horizontal gene transfer, and ILS. We adopted the instability score implemented in MIPhy[40]. The score represents *i*) the product of the duplications and loss events, and *ii*) the "relative spread" of the dissimilarities between the OG topology and the species tree. It is therefore a representation of the incongruence of a cluster of genes throughout their evolutionary history. We coupled the instability score with the gene turnover rate computed with CAFE, to identify which families have higher duplication rates, and may therefore contribute to altered or gain of

function. The number of OGs per gene family varied from one OG, in the families of Cuticle protein CPCFC or DMT family transporters, to the ~250 OGs, rich families of Trypsins and Protein kinases. The average instability score is 37.45 while the average λ is 0.005. The number of OG per family moderately correlates with λ (Pearson's $\rho = 0.42$), but not with mean instability scores, whereas the two measures (λ and mean instability scores) broadly correlated with each other (Pearson's $\rho = 0.37$), once a single clear outlier was removed. The outlier gene family (GF) (here defined as the set of all related OGs) corresponds to the cuticle protein CPCFC (PF17223), which has a λ of 0.006 and IS of 146.92, a very high value. This GF experienced a duplication event at the base of *Heliconius* (Fig. 5a; supplementary Fig. 38, supplementary data 8), and independent duplications within Sara-Sapho + Erato and Silvaniform/Melpomene clades (supplementary Fig. 38). Among the most stable GFs with more than ten OGs there are PQ-loop repeat-containing proteins, UAA transporter family, Rhodanese-like domain, ZIP family metal transporter, and Permease family, while Ion transport proteins, the Beta-glucosidase 2 glycosyl-hydrolase family, SNF domain-containing proteins, Sodium/calcium exchanger proteins, and Hemocyanin superfamily have the highest instability and turnover rates (supplementary Fig. 39). At the base of the phylogeny, from the Heliconiinae to *Heliconius* + *Eueides* branches, the dataset shows mostly OG family expansions. Some of the GFs most affected are the Hemocyanin superfamily, Lipase, Trypsin and Sugar transporter family from the Major Facilitator Superfamily (Fig. 5a). We identify a series of notably large expansions within the Hemocyanin superfamily, with one expansion event in the *Dione* + *Agraulis* + *Eueides* + *Heliconius* clade, and another involving *Eueides* and *Heliconius*. The first expansion corresponds to a basal hexamerin homolog, and the second to an arylphorin, a lepidoptera specific hexamerin[41], which expanded independently in the two clades. Hexamerins do not bind copper and thus oxygen, but instead function as storage proteins, providing amino acids and energy for non-feeding periods, such as molting and pupation[42], and may also transport hormones[43]. Curiously, a contraction in the Hemocyanin superfamily was only observed in *H. aoede*, the only *Heliconius* species not to feed on pollen in our data, marking hexamerins a potential mechanistic link to the divergent strategies for nitrogen storage in pollen-feeding *Heliconius* (Fig. 5a).

Finally, we evaluated whether different evolutionary pressures existed between *Eueides* and *Heliconius* among OGs, within in the selected GFs. Seven GFs showed a significant difference between the two genera in the distribution of ω (Fig. 5b), with *Heliconius* species having more high values of ω. To understand the direction of the shift we calculated K[44], the relaxation index. When $K < 1$, the parameter indicates a general relaxation, while $K > 1$ indicates an intensification of selection. We observed that among the GFs which differ between *Heliconius* and *Eueides*, relaxed selection is generally the cause, but an intensification of positive selection is identified in some GFs, including Trypsin, Protein kinase, P450,

Hemocyanin, Sugar transporters, Ion transporters and ABC transporter. In these GFs, at least a fifth of the genes that show a significant shift in selective pressure between genera are under the intensification of positive selection in *Heliconius* (Fig. 5b). In the Hemocyanin superfamily two genes are hexamerin storage proteins, while in the P450 a *CYP303A1*-like gene (supplementary Fig. 40), a highly conserved protein in insects which has a pivotal role in embryonic development and adult moulting in *Locusta migratoria* and *Drosophila melanogaster*[45].

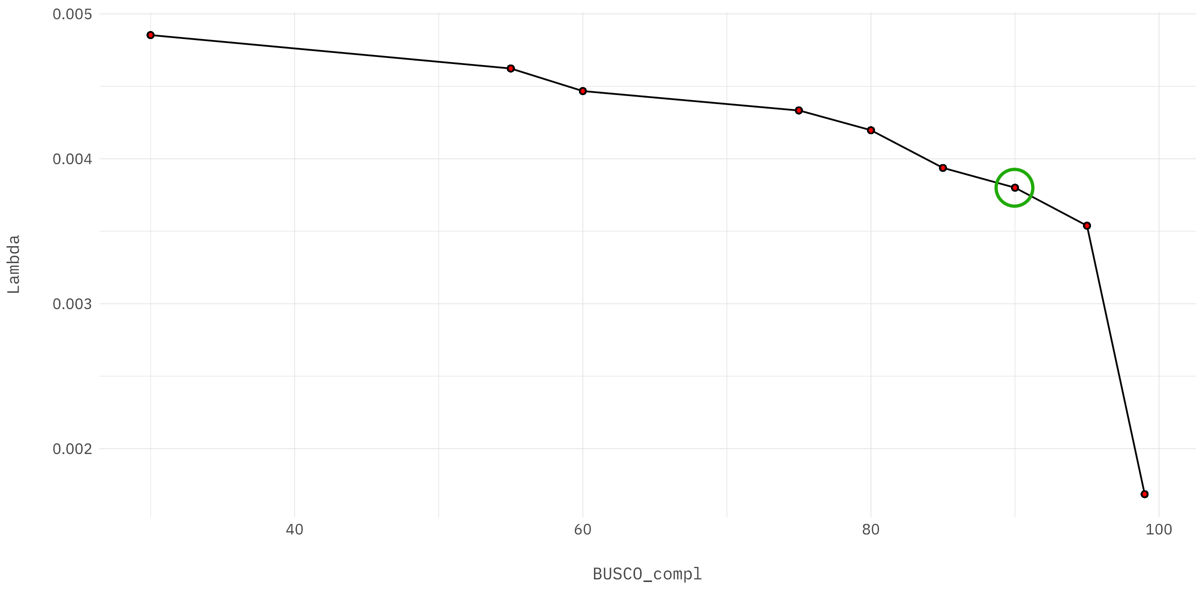

Supplementary Fig. 37 | Dataset selection for CAFE. *Diagnostic test to ensure the robustness of the assessment of the Nymphalid rate of gene gain and loss (λ) with respect to genome completeness (complete BUSCO gene %). The green circle indicates the threshold used (assembly with 90% complete BUSCO genes). Each point corresponds to 10 independent runs.*

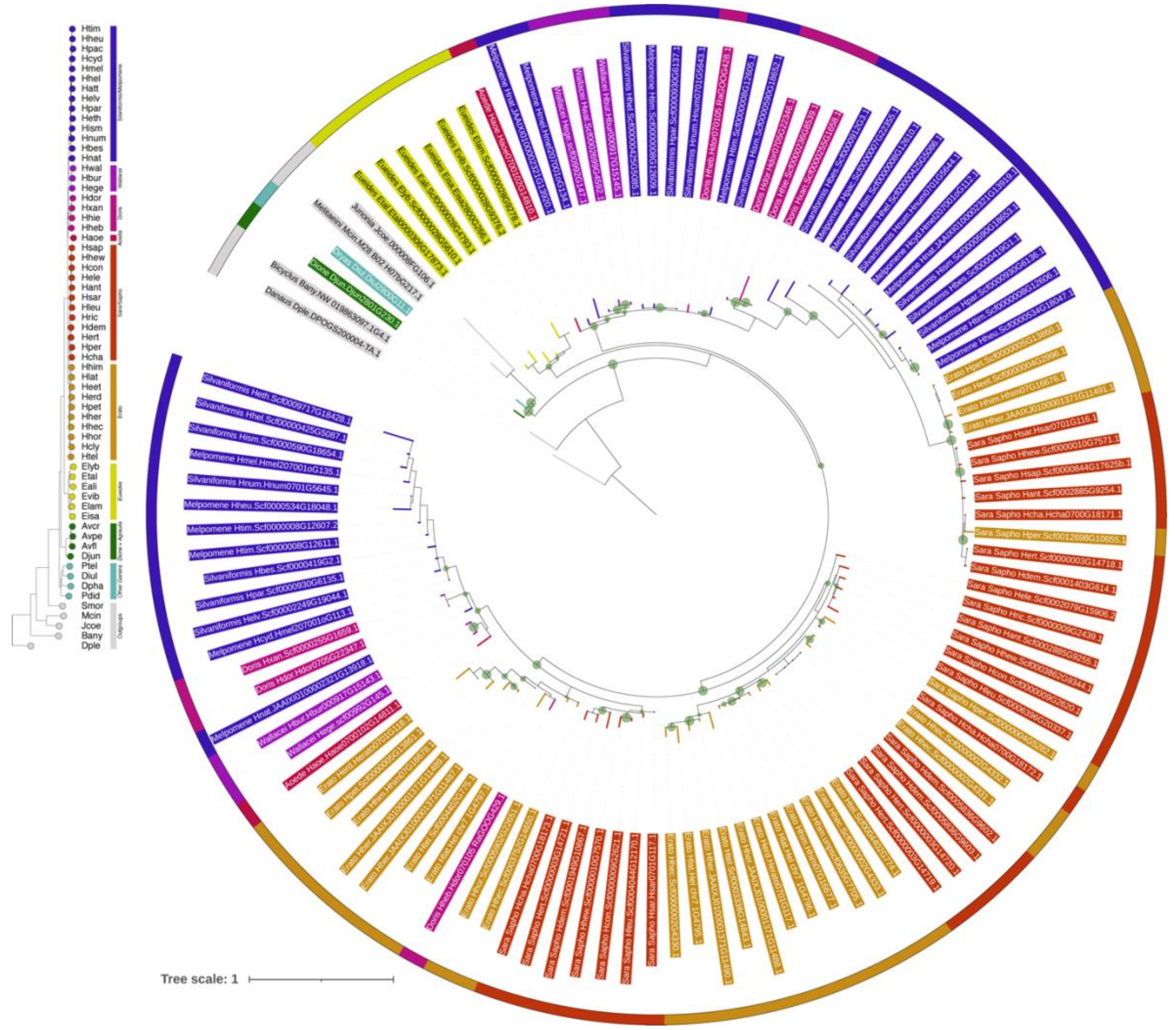

Supplementary Fig. 38 | Cuticle protein CPCFC (PF17223) phylogeny. *ML amino acid phylogenetic tree (Iq-Tree2) of the Cuticle protein CPCFC gene family indicating an expansion in Heliconius species. Green circles at nodes indicate the bootstrap values > 0.80 (1000 ultrafast bootstrap replicates), and the size is proportional to their values.*

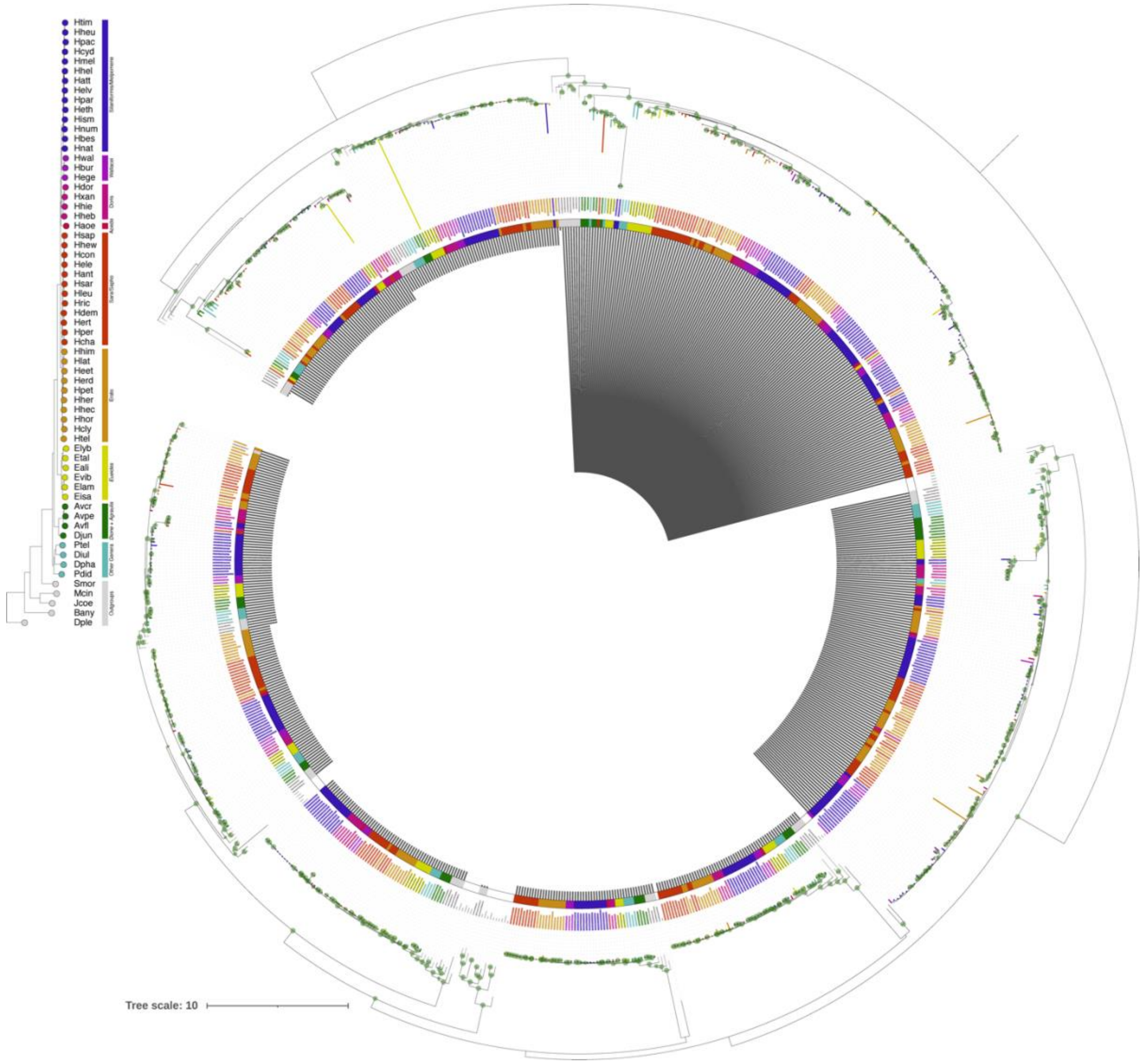

Supplementary Fig. 39 | Hemocyanin superfamily (PF00372) phylogeny. *ML amino acid phylogenetic tree (Iq-Tree2) of the Hemocyanin superfamily indicating two OG with high phylogenetic instability scores (grey histograms within the circle) compared with the other OGs. Green circles at nodes indicate the bootstrap values > 0.80 (1000 ultrafast bootstrap replicates), and the size is proportional to their values.*

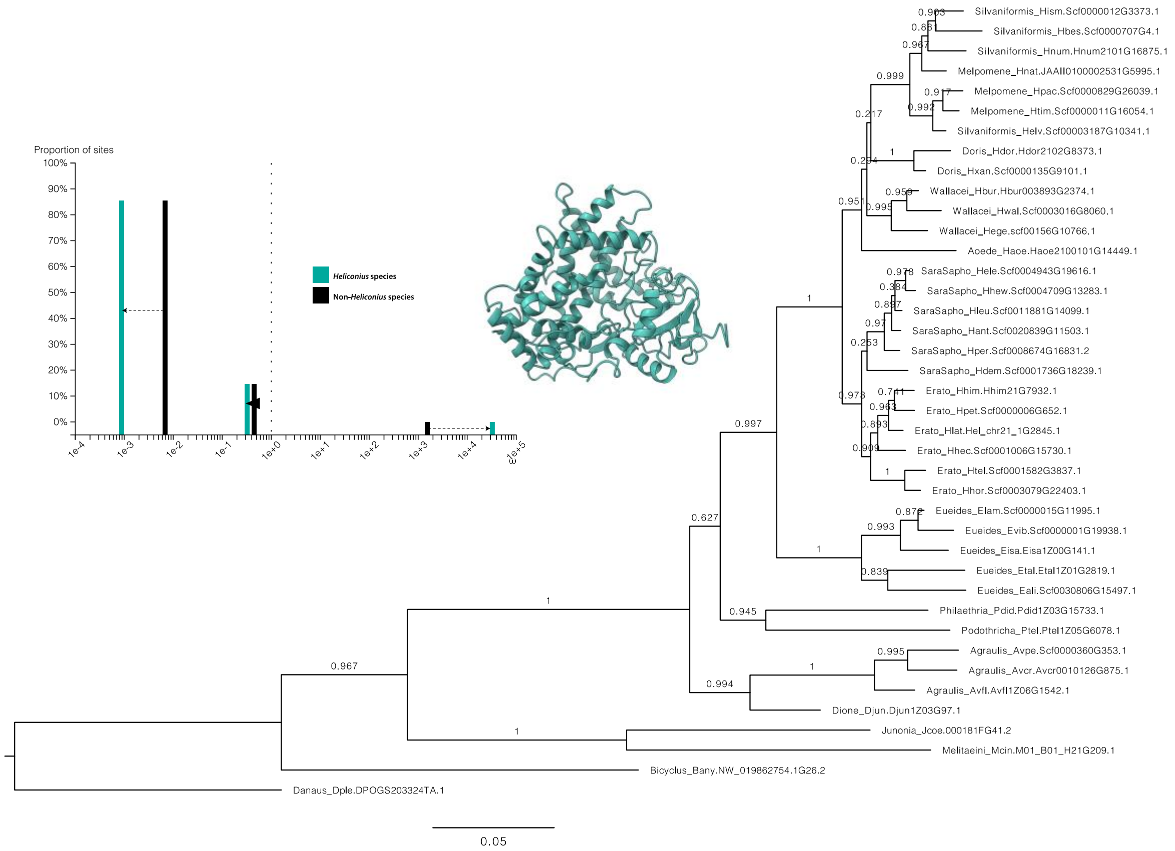

Supplementary Fig. 40 | Intensification of CYP303A1 phylogeny. *The RELAX analysis shows a significant intensification of CYP303A1 gene in Heliconius species (K = 1.41; P value = 0.011, LR = 6.43). Values at branches indicate bootstrap values. The arrows in the histogram show the direction of the three partitions. The partitions of the purifying and diversifying selection are more shifted in Heliconius species. Predicted 3D structure of the putative aa sequence from H. melpomene.*

Supplementary Note 6. Selection Across Heliconiinae Genomes and the Heliconiini Radiation

The impact of evolutionary pressures on Heliconiinae coding genes was further investigated adopting the adaptive branch-site random effects likelihood (aBSREL) method[46,47] on the 3,393 scOGs. This method allows sites to evolve under different selection regimes (ω < 1; = 1; > 1), and adapts its model complexity to the data, inferring the optimal number of rate categories to be used for each branch. Interestingly, when classified according to their phylogenetic attribution (*i.e.,* where they appeared throughout the phylogeny), scOGs show a significant trend for increasing purifying selection with time, from the genes with most recent origins to the most ancient ones (*i.e.,* from those specific to *Eueides* + *Heliconius*, towards Heliconiinae and Nymphalids) (Fig. 5c). This suggests that as genes become more stable across phylogenetic time, which likely reflects increased biological importance they become more conserved. The signature of diversifying positive selection was also assessed on five of the key Heliconiinae branches: those leading to the base of Heliconiini (2,829 tested genes), *Dione* + *Agraulis* + *Eueides* + *Heliconius* (2,317), *Eueides* + *Heliconius* (3,018), *Eueides* (3,276), and *Heliconius* (3,163). Comparing the branches, both the mean ω values and the

percentage of the gene under a diversifying positive selection ($\omega > 1$), showed that the Heliconiini branch evolved under the strongest selection (median ω and percentage of gene of 0.083 and 0.043, respectively), followed by the *Eueides* and *Heliconius* branches, and finally by *Eueides* + *Heliconius* branch (Fig. 5d).

The number of genes with a signal of diversifying positive selection varies between branches (*P*-adjusted < 0.05), with the stem branch of *Eueides* + *Heliconius* having the highest number of loci (96), followed by the stem *Dione* + *Agraulis* + *Eueides* + *Heliconius* branch (94), *Eueides* (89), Heliconiini (70), and finally the *Heliconius* (56 loci; supplementary data 9) branches (*P* values < 0.05, and GOstats ∩ GOATOOLS, see Methods; supplementary data 10). These numbers do not correlate with enriched biological processes (BPs) and molecular functions (MFs). Indeed, the stem *Dione* + *Agraulis* + *Eueides* + *Heliconius* and *Heliconius* branches have the highest number of enriched BPs, with 32 and 29, respectively; followed by Heliconiini and *Eueides* branches (21 enriched BPs), and *Eueides* + *Heliconius* with 19. Although the enriched BPs vary across branches (supplementary data 11), a notable pattern is the high proportion of branches enriched for BPs relating to neuronal development and related cellular functions, including regulation of hippo signalling, stem cell differentiation and cell-cell adhesion mediated by cadherin. For example, on the stem *Heliconius* branch, diversifying selection was found on genes associated with asymmetric division, while on the stem *Euiedes* + *Heliconius* branch there is enrichment of genes linked to the regulation of dendrite development. We also note BPs related to cuticle and wing development (stem *Eueides*), DNA methylation (stem *Heliconius*) and eye photoreceptor development (stem *Euiedes* + *Heliconius*). We next used a network based approach, which integrates both primary and predicted interactions to predict gene function [48,49], to examine connections between selected genes. Interestingly, although the amount of network interactions shows a significant degree of connectivity (absolute number of interactions) in the branches leading to *Dione* + *Agraulis* + *Eueides* + *Heliconius* (834), *Eueides* + *Heliconius* (627), *Eueides* (531), Heliconiini (410 interactions), and *Heliconius* (320), the network density shows a different picture, with *Heliconius* having a higher density (the portion of the potential connections in a network that are actual connections) with ~0.3 versus ~0.2 for the other networks, in case of BP networks (supplementary Fig. 41, supplementary data 11). While the Heliconiini network is mostly enriched with MFs associated to transmembrane transporter activity, and other metabolic processes, the *Dione* + *Agraulis* + *Eueides* + *Heliconius* network is enriched with terms associated with synaptic membrane, cell-cell junction, apoptosis involved in cell development, learning or memory, cognition, and cell junction assembly. The *Eueides* + *Heliconius* network is enriched for regulation of cell projection organization and neuron projection development (FDR > 0.05). The *Eueides* network is enriched for many BP and MF terms associated with chitin development and regulation of pH, which is potentially linked to the necessity for the larval gut to

have an alkaline pH to feed on *Passiflora* without creating cyanide poisoning[32]. The *Heliconius* network is characterised by BPs related to response to DNA damage/repair, and several terms all related to neuroblast division and stem cell and neural precursor cell proliferation, glial cell development, cell-cell junction assembly, asymmetric stem cell division (supplementary Fig. 42).

There are multiple genes that in our dataset show a signature of diversifying positive selection in more than one branch, specifically there are 36 and 9 genes that experienced selection two and three times in the five branches, respectively. One of them is a *Notch*-like gene which codifies an essential signalling protein, with major roles in many developmental processes, such as the regulation of oogenesis, the differentiation of the ectoderm and the development of the central and peripheral nervous system, eye, wing disks, muscles, and segmental appendages[50]. Notch regulates neuroblast self-renewal, identity and proliferation in larval brains, and it is involved in the maintenance of type II neuroblast self-renewal and identity[51,52].

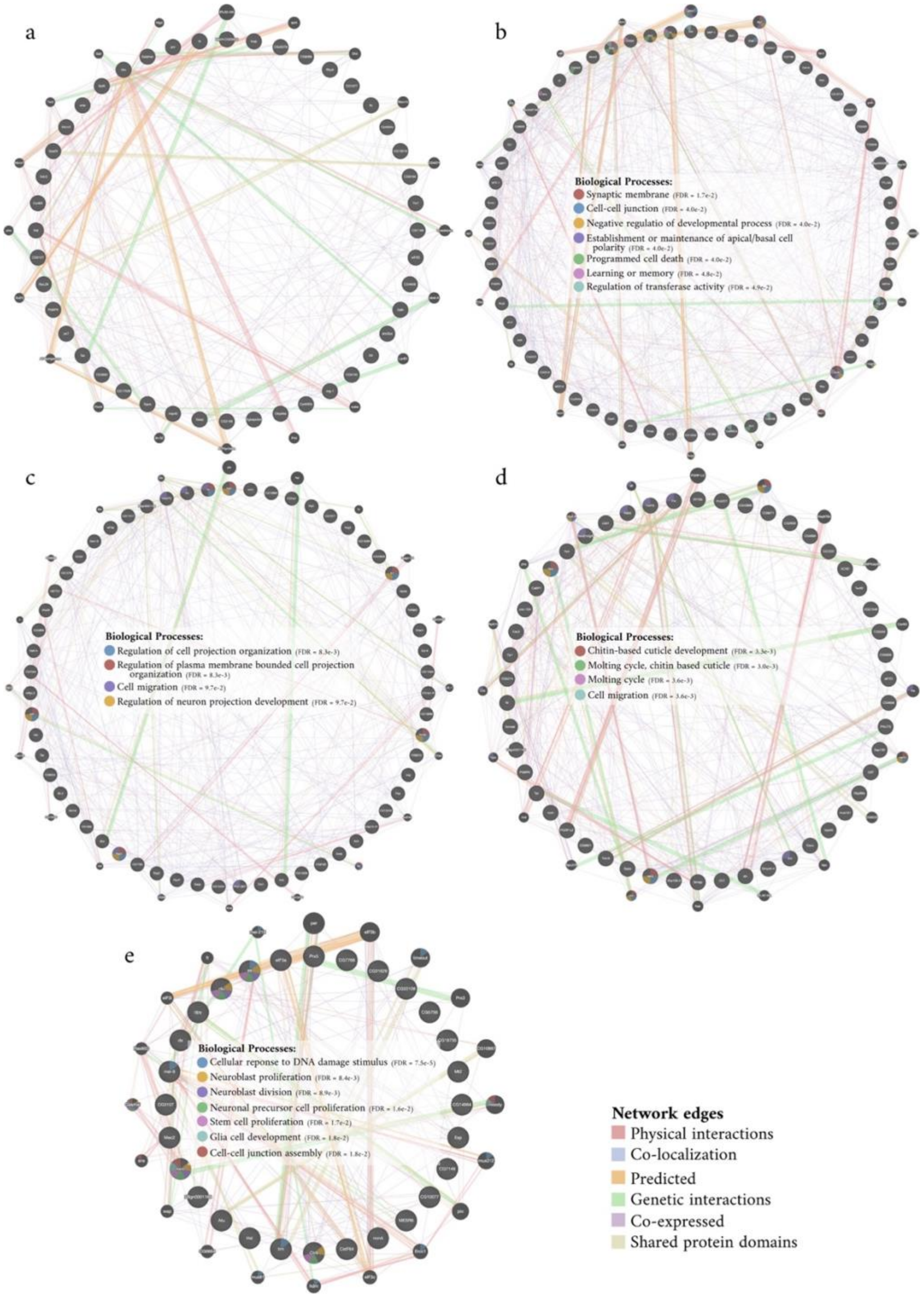

***Supplementary Fig. 41 | GeneMania Biological processes**. Extract from Fig. 5d in the main text. Networks of interacting genes analysed by GeneMANIA. Inner circles represent loci under diversifying positive selection for each focal branch, connecting with other genes (outside the circle) according to physical interaction, co-expression, co-localization, and others (coloured lines in networks). The method, implemented in GeneMANIA, creates a gene interaction network were different nodes (i.e., genes) are connected by edges, which represent data derived from physical interaction, co-expression, co-localization, and others (coloured lines in networks). This in turn, is used to extract information on their biological processes and molecular functions (GO term) to test for enrichment (see supplementary data 10).*

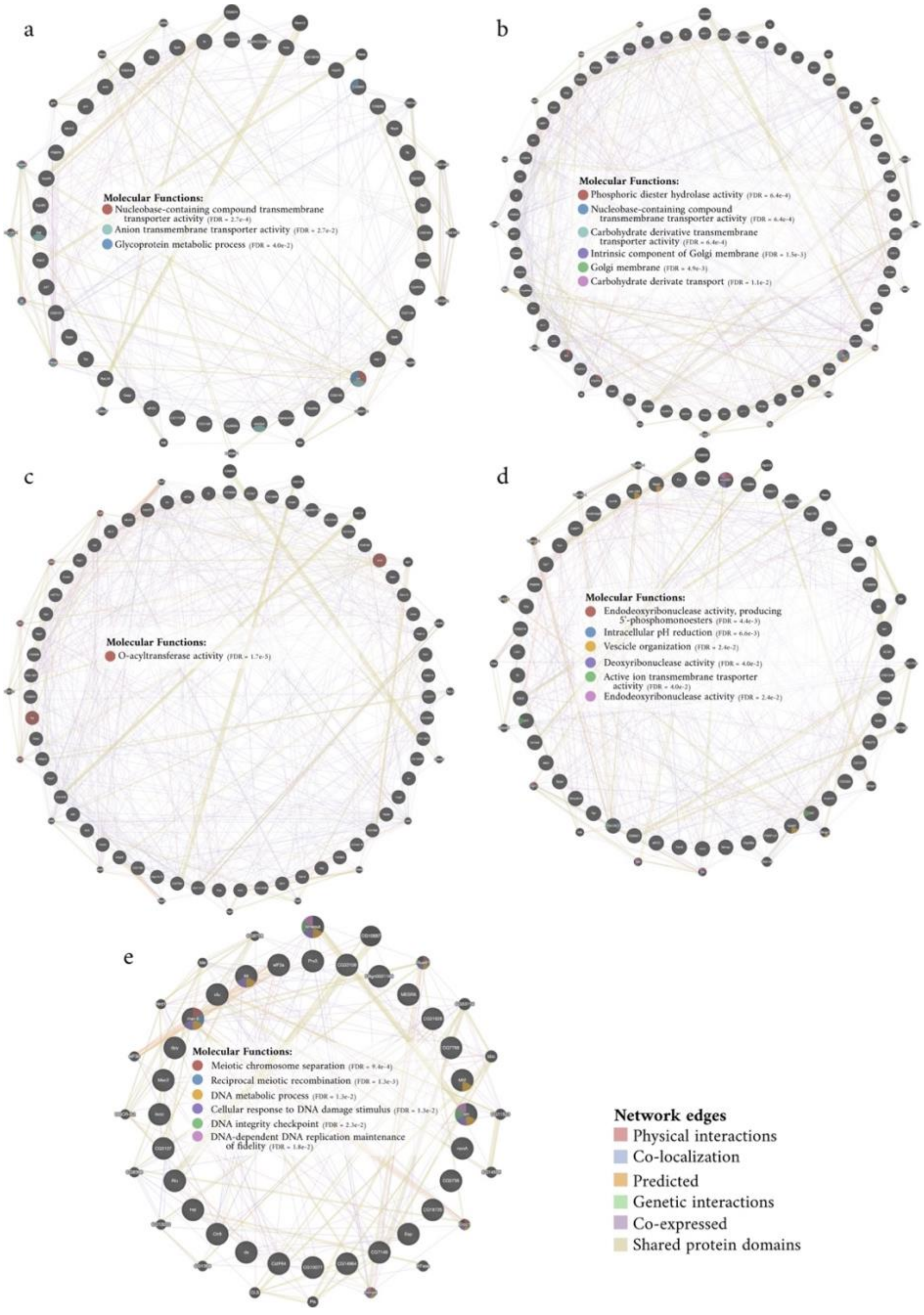

Supplementary Fig. 42 | GeneMANIA Molecular function. *Extract from Fig. 5d in the main text. Networks of interacting genes analysed by GeneMANIA. Inner circles represent loci under diversifying positive selection for each focal branch, connecting with other genes (outside the circle) according to physical interaction, co-expression, co-localization, and others (coloured lines in networks). The method, implemented in GeneMANIA, creates a gene interaction network were different nodes (i.e., genes) are connected by edges, which represent data derived from physical interaction, co-expression, co-localization, and others (coloured lines in networks). This in turn, is used to extract information on their biological processes and molecular functions (GO term) to test for enrichment (see supplementary data 11).*

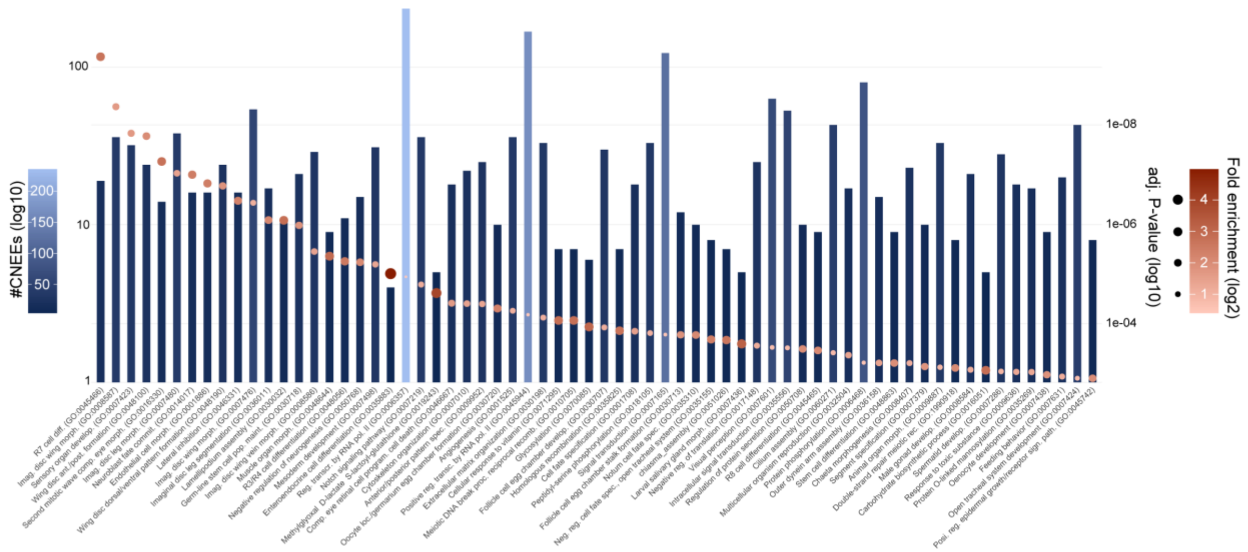

Supplementary Fig. 43 | GO term enrichment of aCNEEs. *Histogram showing the number of aCNEEs for each enriched GO term and scatter plot showing the corresponding adjusted P-value and the fold enrichment expressed as circle size and color intensity.*

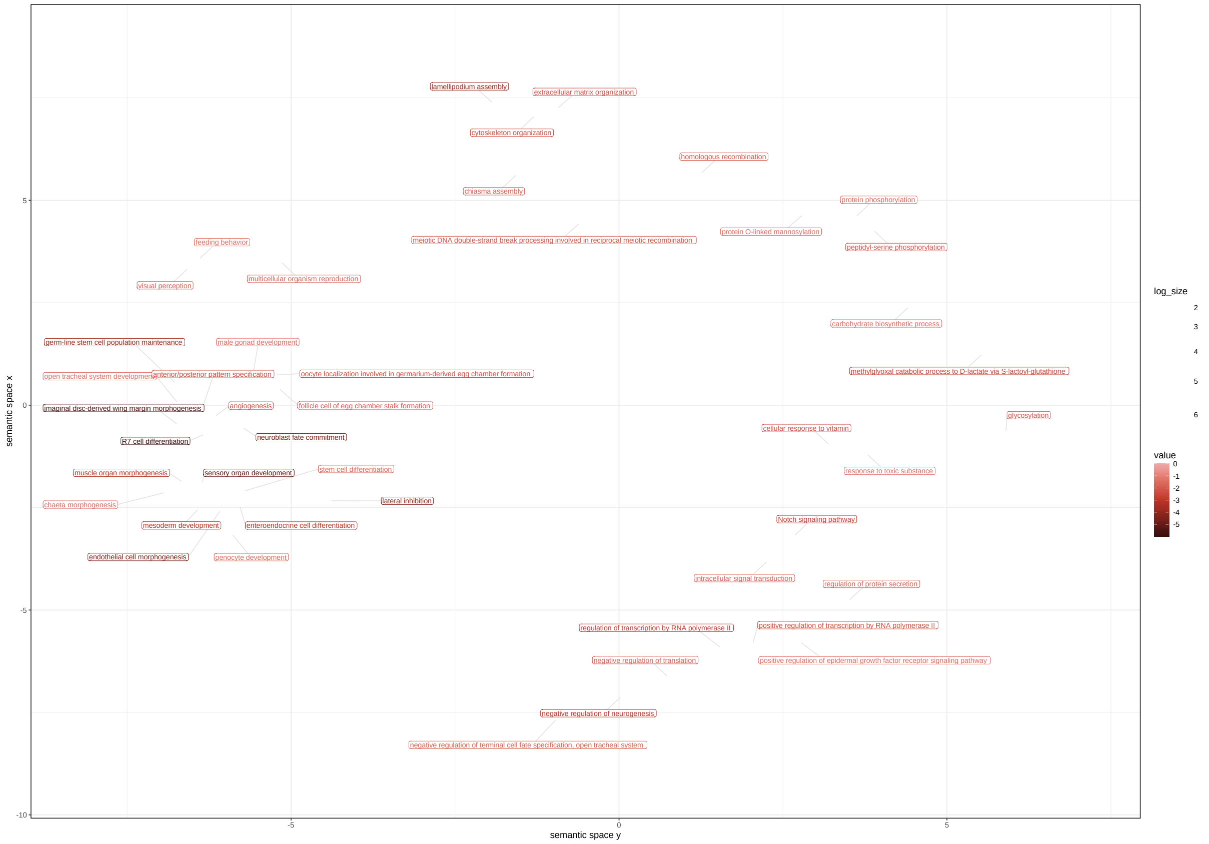

Supplementary Fig. 44 | GO term enrichment of aCNEEs. *Semantic space of enriched GO terms representing the similarity among terms, in color/size the corresponding adjusted P-values.*

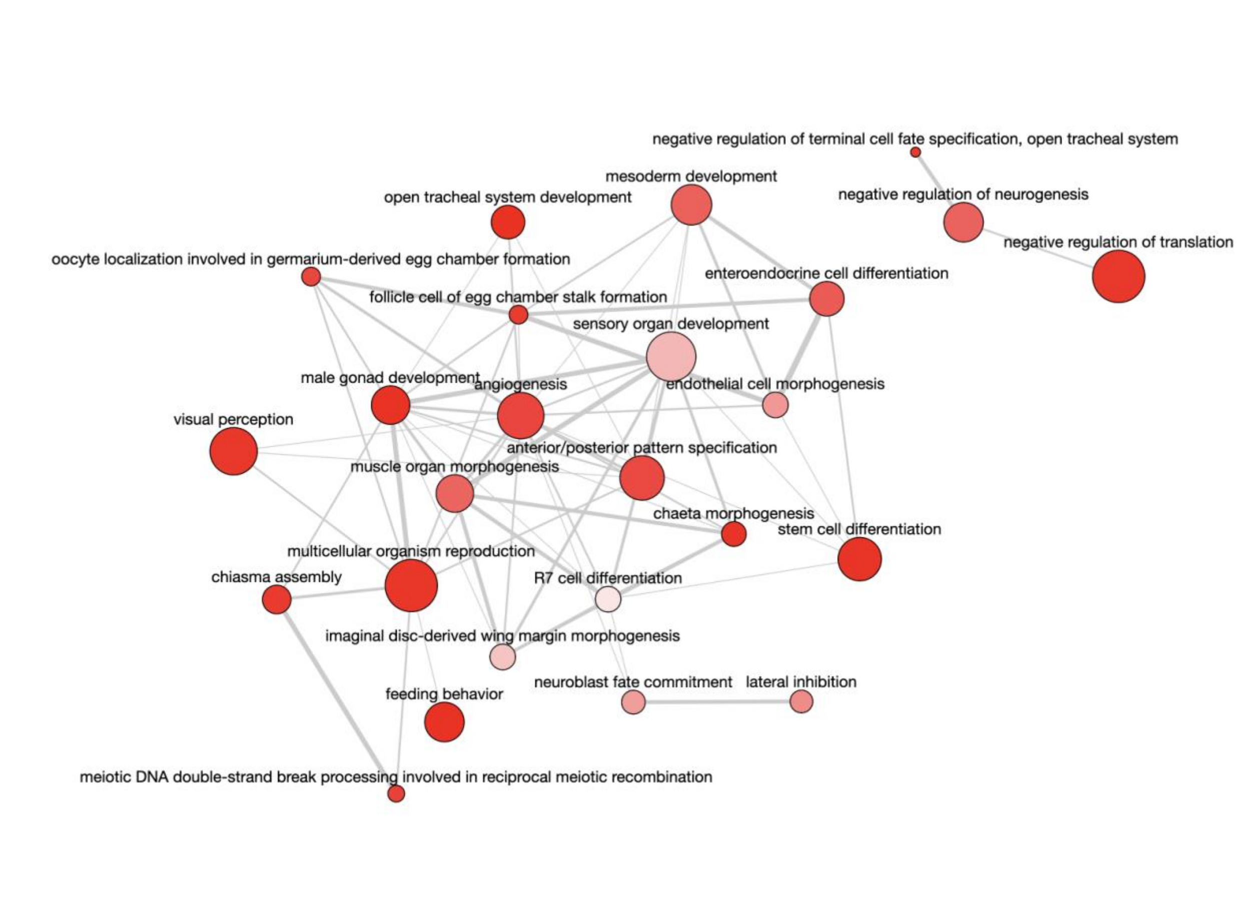

Supplementary Fig. 45 | GO term enrichment of aCNEEs. *Integrative maps of the enriched GO term network. Highly similar GO terms are linked by edges in the graph, where the line width indicates the degree of similarity.*

Supplementary Note 7. Tribe Wide Genomics Highlight Candidate Genes for Derived Traits.

Within the Heliconiinae, *Heliconius* displays a number of divergent traits and innovations that are thought to be associated with the evolution of pollen feeding[53]. Here, we exemplify how our results reveal new biological insights into two these traits, focusing on mushroom body expansion, and the enzymatic processes associated with breaking down pollen walls to aid digestion of pollen grains.

i) Mushroom body expansion: Mushroom bodies are paired organs within the central brain that receive visual and/or olfactory information, and play a pivotal role in learning and memory[54]. These structures show huge variation across Heliconiini with major expansions in the stem *Heliconius* branch, and independent increases elsewhere[55–57]. The molecular mechanisms underpinning these events are unknown, but the results of our selection analyses highlight multiple pathways that could regulate neural proliferation. One of these is the Hippo pathway which regulates cell growth and proliferation in *Drosophila*, including the proliferation of neural stem cells[58] and neuroblast quiescence[59,60]. In addition to evidence of expansions in Hippo pathway OGs (see above), we found multiple and repeated selection events on genes related to

the Hippo pathway, including *Focal adhesion kinase* (*Fak*), *lethal (2) giant larvae* (*lgl*), *Sarcolemma associated protein* (*Slmap*), *Akt kinase* (*Akt*), all of which show a signal of positive selection in the *Dione* + *Agraulis* + *Eueides* + *Heliconius* branch. These genes have known roles in regulating cell polarity, asymmetric division and cell proliferation[61–64]. Two other genes, *Moesin* (*Moe*) and *F-box and leucine-rich repeat protein 7* (*Fbxl7*), show signs of positive selection in the *Eueides* + *Heliconius* stem. Moe is another polarity protein, which drives cortical remodeling of dividing neuroblasts in *Drosophila*[65], while Fbxl7 regulates the Dachs, which in turn inhibit Hippo pathway activity[66]. Finally, in the stem *Heliconius* branch, other genes like *Ctr9*, *dachsous* (*ds*), *falafel* (*flfl*), and *locomotion defects* (*loco*), play key roles in neuroblast division and differentiation (Fig. 5b, supplementary data 10). For example, in *Drosophila*, Ctr9 is involved in the proliferation and terminal differentiation of the central nervous system[67], flfl is required for asymmetric division of neuroblasts and orchestrates neurogenesis in mushroom bodies[68,69] and may function by establishing cell polarity[70], and *ds*, which encodes a member of the cadherin superfamily, provides another example of a selected gene that interacts with the Hippo pathway[71]. Finally, loco plays a crucial role in glial specification, functioning as activators of glial fate, which constitutes an essential element in providing an efficiently operating nervous system [72].

ii) Cocoonase Evolution in Heliconiinae: Despite being a keystone innovation in *Heliconius*, very little is known about the mechanism underpinning pollen feeding. Saliva appears to be important for the external digestion of pollen grains, and is thought to contain proteases that digest sporopollenin, the primary component of pollen walls[53]. The leading candidates for this role are cocoonases which is secreted from the proboscis of silkworms and digests the silk cocoon during eclosion[73]. Like all butterflies, *Heliconius* do not produce a cocoon, but previous work has shown that cocoonase underwent several independent duplications in Lepidoptera[73] including the lineage leading to *Heliconius* and their sister genus, *Eueides*, suggesting they may have been co-opted for pollen feeding[74]. Here we used our expanded phylogenomic dataset and novel methodological approaches[75,76], to computationally assess levels of functional divergence, and further test this hypothesis.

We identified 233 cocoonase loci across Heliconiinae (supplementary data 15) which cluster into four main OGs (Fig. 7a, b). This reveals that the duplications not only predate the split between *Heliconius* and *Eueides,* but affect the whole Heliconiini tribe and its outgroup *S. mormonia*, which has three copies, one in-paralog and one out-paralog. All Heliconiini species with highly complete genomes have at least four copies and the genomic location and synteny of different copies strongly conserved. (Fig. 7b; supplementary data 16). The duplications are therefore likely the result of three independent tandem duplications and, based on their phylogenetic relationships, and we renamed the four OGs into *Coc1A,*

Coc1B, Coc2 and *Coc3* (Fig. 7a). Most Heliconiini clades have a consistent four copy pattern, including the non-pollen feeding *Heliconius* species, *H. aoede*, which does not show any sign of pseudogenisation, but some species have divergent patterns caused by translocation and further duplications. We quantified levels of purifying selection acting across the four OGs and found that ω varies little among them, with *Coc1B* showing the strongest purifying selection (ω = 0.14; 175 sites under purifying selection; one under positive selection) and *Coc2* the lowest (ω = 0.37; 111 sites under purifying selection; six under positive selection). A scan of all internal branches for signs of diversifying positive selection, shows that only six are putatively under selection (*P*-adjusted < 0.05) (Fig. 7a). Two of these selection events (Fig. 7a) incorporate *H. aoede* loci, so we subsequently tested if those loci show a sign of relaxation, to explore whether the wider pattern of positive selection could be due to pollen-feeding behaviour, under the assumption this would be relaxed in the non-pollen feeding lineage. Interestingly, for *Coc1A* the test indicates an intensification of positive selection (*K* = 1.41; *P*-value = 0.001), while for *Coc1B* we find no significant differences.

We next modelled the 3D protein structure of 181 full-length protein sequences (see Methods) inferring the key residues driving the difference among the overall protein structures of the four loci, adopting a new graph-based theory approach[76] to cluster proteins (supplementary Fig. 47; see Methods). This method identifies distributions of divergent residue positions that significantly different between OGs (P-value < 0,05). The Melpomene/Silvaniform *Coc2* sub-clade, which evolved under diversifying positive selection, is clustered away from its sister *Coc2* clade and the other 3 OGs. Furthermore, using the loading of each original variable (*i.e.,* the strength values associated with each residue position) we identified the residues most responsible for the clustering of OGs and mapped them onto the 3D structure of each group centroid. We observed the typical power law trend (supplementary Fig. 48), where only a few residues drive the clustering of OGs. In this case, the loadings of the first seven residues total 60% of the total loadings of all 227 positions in the structural alignment, suggesting that these residues have particular functional value which can maximise the differences between the four OGs in terms of their residue interaction network. Interestingly, the seven identified positions cluster into three distinct regions in the 3D structures (Fig. 7d), corresponding to three loops in regions highly exposed to the solvent; two of them (pos: 217-217 and pos: 119-122) face each other at the back of the active cleft. Comparing the modelled cocoonase structures with the x-ray model of several homologous serine proteases through a structural alignment (supplementary Fig. 49), we determine that the two largest loops (pos: 217-217 and pos: 119-122) corresponds to regions with high b-factor scores (high fluctuation), contrasting with the shortest third loop (pos: 68-71), which corresponds to a region with lower b-factor values (high stability). This is followed by the consistent presence of specific amino acids in those regions (Fig. 7d), an indication that the cocoonases codified by

these duplicated genes might have gained the capacity to bind and process different substrates by changing their flexibility and therefore acquiring other roles throughout the radiation of Heliconiini.

Our combined results therefore suggest that there is functional divergence across the Coc proteins in Heliconiini, but that this functional divergence is phylogenetically conserved with the exception of a split between the two halves of the *Heliconius* tree for Coc2. These findings present a more complex story than previously described, and both the high copy number variation and patterns of selection within Heliconiinae appear inconsistent with these genes playing a critical role in the evolution of pollen feeding. As such, our analyses highlight that more complete phylogenomic data can be critical for hypothesis testing, while elsewhere suggesting alternative candidate genes which could play a role in the evolution of pollen feeding and associated traits.

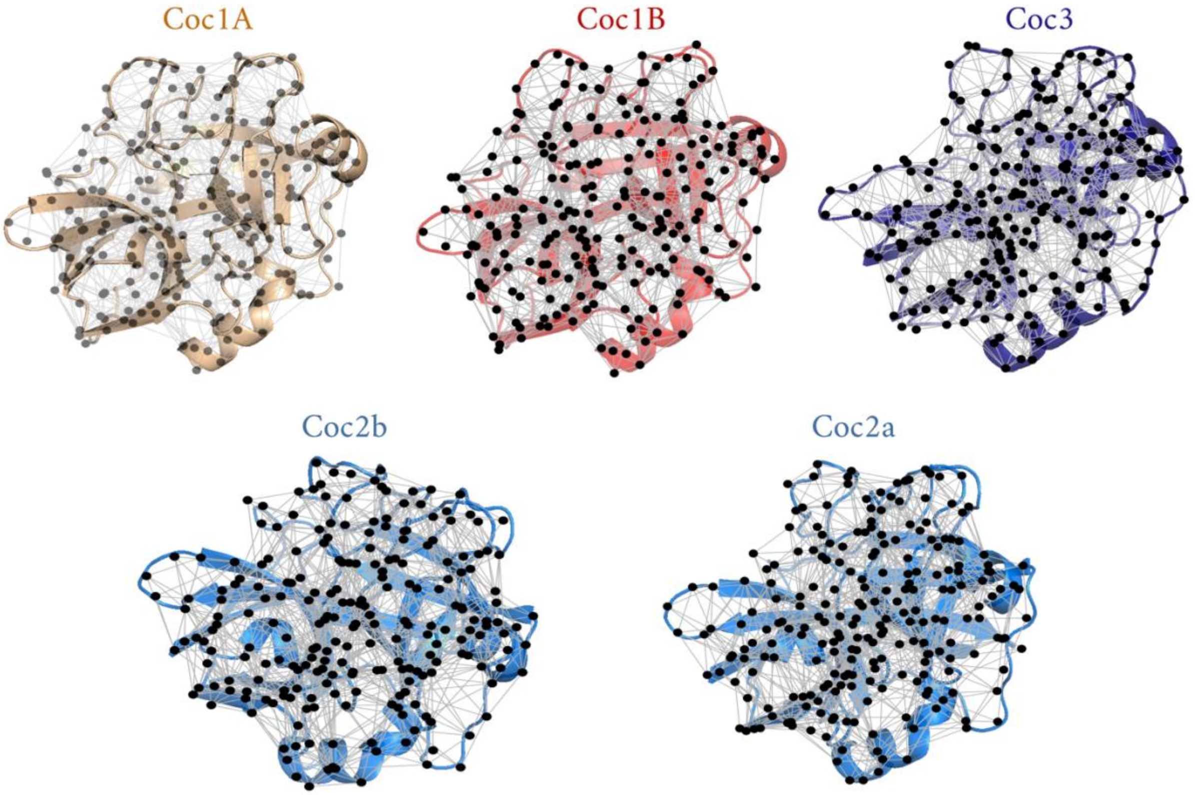

Supplementary Fig. 46 | Graph view of intramolecular interactions. *For each representative structure identified by the PCA, a graph view of intramolecular interactions is shown. Each alpha carbon of the protein is represented as a node of the graph and each interaction (defined on the basis of distance) is represented as a link. Two neighboring nodes in the structure are connected by a link in the graph representation.*

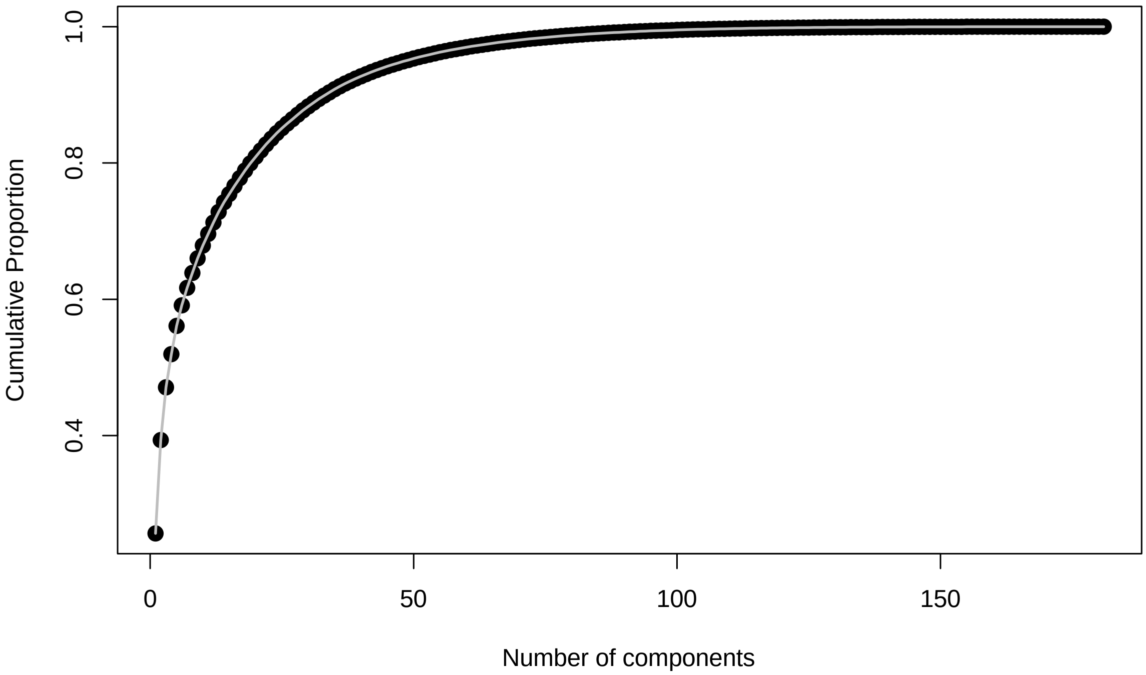

Supplementary Fig. 47 | Cumulative proportion of PCA components. *The cumulative proportion of all components of the Principal Component Analysis. Each component contributes a certain percentage to the explanation of the total variance. The curve shows that the first two principal components explain about 40% of the total variance of the data.*

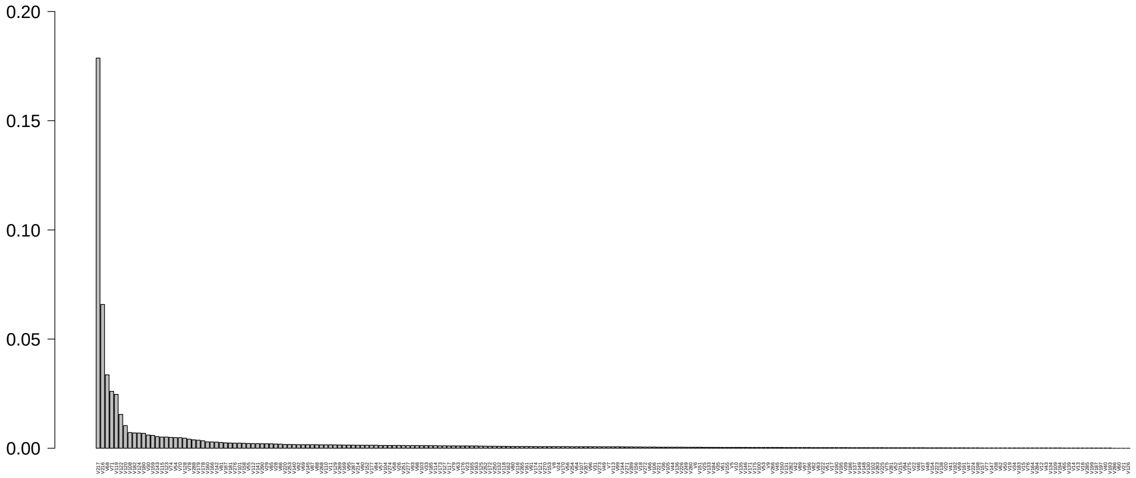

Supplementary Fig. 48 | Power law trend of PCA components Cumulative. *The total loading of the first components for all residues considered in this study are reported in descending order. The total loading of each residue was calculated by multiplying the two loading values of the first two principal components. The data trend is a typical power law, meaning that only a few residues have most of the loading information (i.e. a few residues with high loading values and many residues with low loading values).*

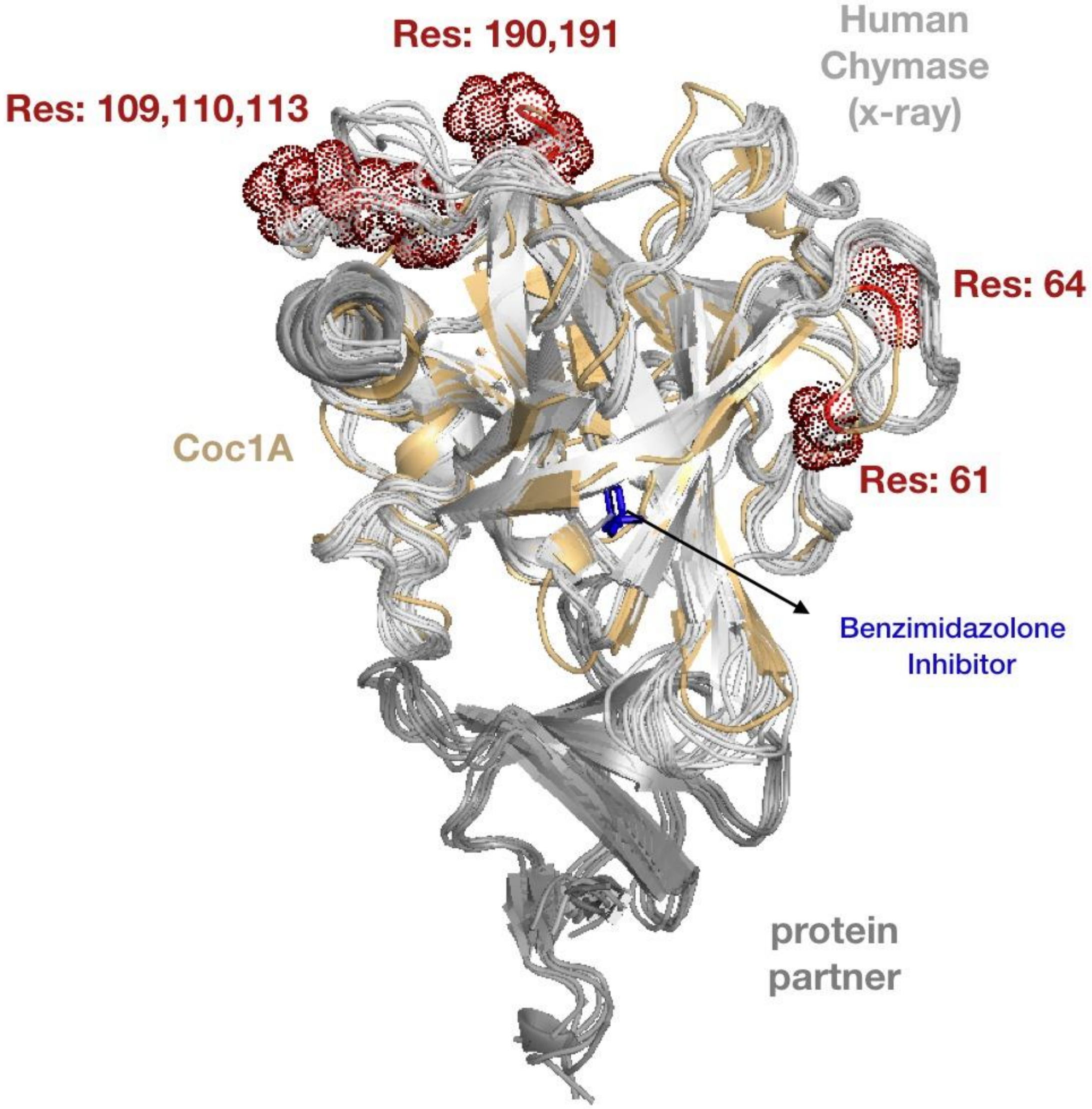

Supplementary Fig. 49 | Structural alignment of cocoonase 1A with X-ray structures of human chymases. *The structural alignment of 20 protein crystal structures of human chymases with the representative sequence of Coc1A from Dr. phaetusa). In light gray the 20 chymases, in dark gray the binded Fynomer CoSmplexes, while in gold the DphaCoc1A. The red mash spheres represent the identified regions of interest with their relative positions in the sequence.*

Supplementary References

1. Kozak, K. M. *et al.* Multilocus species trees show the recent adaptive radiation of the mimetic heliconius butterflies. *Syst. Biol.* **64**, 505–524 (2015).
2. Núñez, R. *et al.* Integrative taxonomy clarifies species limits in the hitherto monotypic passion-vine butterfly genera Agraulis and Dryas (Lepidoptera, Nymphalidae, Heliconiinae). *Syst. Entomol.* **47**, 152–178 (2022).
3. Lewis, J. J. *et al.* The *Dryas iulia* genome supports multiple gains of a W chromosome from a B chromosome in butterflies. *submitted* (2021).
4. Waterhouse, R. M. *et al.* BUSCO applications from quality assessments to gene prediction and phylogenomics. *Mol. Biol.*

Evol. **35**, 543–548 (2018).

5. Cicconardi, F. *et al.* Chromosome Fusion Affects Genetic Diversity and Evolutionary Turnover of Functional Loci but Consistently Depends on Chromosome Size. *Mol. Biol. Evol.* **38**, 4449–4462 (2021).
6. Ray, D. A. *et al.* Simultaneous TE Analysis of 19 Heliconiine Butterflies Yields Novel Insights into Rapid TE-Based Genome Diversification and Multiple SINE Births and Deaths. *Genome Biol. Evol.* **11**, 2162–2177 (2019).
7. Fiddes, I. T. *et al.* Comparative Annotation Toolkit (CAT) - simultaneous clade and personal genome annotation. *Genome Res.* 231118 (2018) doi:10.1101/231118.
8. Armstrong, J. *et al.* Progressive alignment with Cactus: A multiple-genome aligner for the thousand-genome era. *bioRxiv* (2019) doi:10.1101/730531.
9. Edelman, N. B. *et al.* Genomic architecture and introgression shape a butterfly radiation. **599**, 594–599 (2019).
10. Massardo, D. *et al.* The roles of hybridization and habitat fragmentation in the evolution of Brazil's enigmatic longwing butterflies, Heliconius nattereri and H. hermathena. *BMC Biol.* **18**, 84 (2020).
11. Sculfort, O. *et al.* Variation of chemical compounds in wild Heliconiini reveals ecological factors involved in the evolution of chemical defenses in mimetic butterflies. *Ecol. Evol.* **10**, 2677–2694 (2020).
12. Dasmahapatra, K. K., Silva-Vásquez, A., Chung, J. W. & Mallet, J. Genetic analysis of a wild-caught hybrid between non-sister Heliconius butterfly species. *Biol. Lett.* (2007) doi:10.1098/rsbl.2007.0401.
13. Mallet, J., Beltrán, M., Neukirchen, W. & Linares, M. Natural hybridization in heliconiine butterflies: The species boundary as a continuum. *BMC Evol. Biol.* (2007) doi:10.1186/1471-2148-7-28.
14. Kozak, K. M., Joron, M., McMillan, W. O. & Jiggins, C. D. Rampant Genome-Wide Admixture across the Heliconius Radiation. *Genome Biol. Evol.* **13**, 1–17 (2021).
15. Suvorov, A. *et al.* Widespread introgression across a phylogeny of 155 Drosophila genomes. *Curr. Biol.* 1–13 (2021) doi:10.1016/j.cub.2021.10.052.
16. Walters, J. R., Corbins, C., Hardcastle, T. J. & Jiggins, C. D. Evaluating female remating rates in light of spermatophore degradation in Heliconius butterflies: Pupal-mating monandry versus adult-mating polyandry. *Ecol. Entomol.* **37**, 257–268 (2012).
17. Thurman, T. J., Brodie, E., Evans, E. & McMillan, W. O. Facultative pupal mating in Heliconius erato: Implications for mate choice, female preference, and speciation. *Ecol. Evol.* **8**, 1882–1889 (2018).
18. Edelman, N. B. *et al.* Genomic architecture and introgression shape a butterfly radiation. *Science (80-.).* **366**, 594–599 (2019).
19. Thawornwattana, Y., Seixas, F. A., Yang, Z. & Mallet, J. Full-Likelihood Genomic Analysis Clarifies a Complex History of Species Divergence and Introgression: The Example of the erato-sara Group of Heliconius Butterflies. *Syst. Biol.* **71**, 1159–1177 (2022).
20. Kapusta, A., Suh, A. & Feschotte, C. Dynamics of genome size evolution in birds and mammals. *Proc. Natl. Acad. Sci. U. S. A.* (2017) doi:10.1073/pnas.1616702114.
21. Pasquesi, G. I. M. *et al.* Squamate reptiles challenge paradigms of genomic repeat element evolution set by birds and mammals. *Nat. Commun.* **9**, (2018).
22. Hjelmen, C. E. *et al.* Genome size evolution within and between the sexes. *J. Hered.* **110**, 219–228 (2019).
23. Byeon, G. W. *et al.* Functional and structural basis of extreme conservation in vertebrate 5′ untranslated regions. *Nat. Genet.* **53**, 729–741 (2021).
24. Murrell, B. *et al.* FUBAR: A fast, unconstrained bayesian AppRoximation for inferring selection. *Mol. Biol. Evol.* **30**, 1196–1205 (2013).

25. Kelley, J. L. *et al.* Compact genome of the Antarctic midge is likely an adaptation to an extreme environment. *Nat. Commun.* **5**, 4611 (2014).
26. Martín-Durán, J. M. *et al.* Conservative route to genome compaction in a miniature annelid. *Nat. Ecol. Evol.* **5**, 231–242 (2021).
27. Ruggieri, A. A. *et al.* A butterfly pan-genome reveals a large amount of structural variation underlies the evolution of chromatin accessibility. *bioRxiv* 2022.04.14.488334 (2022).
28. Sun, C. *et al.* Genus-Wide Characterization of Bumblebee Genomes Provides Insights into Their Evolution and Variation in Ecological and Behavioral Traits. *Mol. Biol. Evol.* **38**, 486–501 (2021).
29. Neafsey, D. E. *et al.* Highly evolvable malaria vectors: The genomes of 16 Anopheles mosquitoes. *Science (80-.).* **347**, (2015).
30. Lage, J. L. Da, Thomas, G. W. C., Bonneau, M. & Courtier-Orgogozo, V. Evolution of salivary glue genes in Drosophila species. *BMC Evol. Biol.* **9**, (2018).
31. Chauhan, R., Jones, R., Wilkinson, P., Pauchet, Y. & Ffrench-Constant, R. H. Cytochrome P450-encoding genes from the Heliconius genome as candidates for cyanogenesis. *Insect Mol. Biol.* **22**, 532–540 (2013).
32. Pinheiro de Castro, É. C. *et al.* The dynamics of cyanide defences in the life cycle of an aposematic butterfly: Biosynthesis versus sequestration. *Insect Biochem. Mol. Biol.* **116**, 103259 (2020).
33. Feyereisen, R. Evolution of insect P450. *Biochem. Soc. Trans.* **34**, 1252–1255 (2006).
34. Opitz, S. E. W. & Müller, C. Plant chemistry and insect sequestration. *Chemoecology* **19**, 117–154 (2009).
35. Du, M. *et al.* Identification of lipases involved in PBAN stimulated Pheromone production in Bombyx mori using the DGE and RNAi approaches. *PLoS One* **7**, (2012).
36. Sung, E. J. *et al.* Cytokine signaling through Drosophila Mthl10 ties lifespan to environmental stress. *Proc. Natl. Acad. Sci. U. S. A.* (2017) doi:10.1073/pnas.1712453115.
37. Delanoue, R. *et al.* Drosophila insulin release is triggered by adipose Stunted ligand to brain Methuselah receptor. *Science (80-.).* (2016) doi:10.1126/science.aaf8430.
38. Niwa, R. *et al.* Juvenile hormone acid O-methyltransferase in Drosophila melanogaster. *Insect Biochem. Mol. Biol.* (2008) doi:10.1016/j.ibmb.2008.04.003.
39. Thomas, J. H. Rapid birth-death evolution specific to xenobiotic cytochrome P450 genes in vertebrates. *PLoS Genet.* **3**, 720–728 (2007).
40. Curran, D. M., Gilleard, J. S. & Wasmuth, J. D. MIPhy: Identify and quantify rapidly evolving members of large gene fam. *PeerJ* **2018**, 1–17 (2018).
41. Burmester, T. Expression and evolution of hexamerins from the tobacco hornworm, Manduca sexta, and other Lepidoptera. *Insect Biochem. Mol. Biol.* **62**, 226–234 (2015).
42. Tang, B., Wang, S. & Zhang, F. Two storage hexamerins from the beet armyworm Spodoptera exigua: Cloning, characterization and the effect of gene silencing on survival. *BMC Mol. Biol.* **11**, (2010).
43. Zhou, X., Tarver, M. R. & Scharf, M. E. Hexamerin-based regulation of juvenile hormone-dependent gene expression underlies phenotypic plasticity in a social insect. *Development* **134**, 601–610 (2007).
44. Wertheim, J. O., Murrell, B., Smith, M. D., Kosakovsky Pond, S. L. & Scheffler, K. RELAX: Detecting relaxed selection in a phylogenetic framework. *Mol. Biol. Evol.* **32**, 1–13 (2014).
45. Wu, L. *et al.* CYP303A1 has a conserved function in adult eclosion in Locusta migratoria and Drosophila melanogaster. *Insect Biochem. Mol. Biol.* **113**, 103210 (2019).
46. Kosakovsky Pond, S. L. *et al.* A random effects branch-site model for detecting episodic diversifying selection. *Mol. Biol. Evol.*

28, 3033–3043 (2011).

47. Smith, M. D. *et al.* Less is more: an adaptive branch-site random effects model for efficient detection of episodic diversifying selection. *Mol. Biol. Evol.* **32**, 1342–1353 (2015).
48. Vlasblom, J. *et al.* Novel function discovery with GeneMANIA: A new integrated resource for gene function prediction in Escherichia coli. *Bioinformatics* **31**, 306–310 (2014).
49. Franz, M. *et al.* GeneMANIA update 2018. *Nucleic Acids Res.* **46**, W60–W64 (2018).
50. Portin, P. & Portin, P. General outlines of the molecular genetics of the Notch signalling pathway in Drosophila melanogaster: A review. *Hereditas* **136**, 89–96 (2002).
51. Li, X., Xie, Y. & Zhu, S. Notch maintains Drosophila type II neuroblasts by suppressing expression of the fez transcription factor earmuff. *Dev.* **143**, 2511–2521 (2016).
52. Li, X., Chen, R. & Zhu, S. bHLH-O proteins balance the self-renewal and differentiation of Drosophila neural stem cells by regulating Earmuff expression. *Dev. Biol.* **431**, 239–251 (2017).
53. Young, F. J. & Montgomery, S. H. Pollen feeding in Heliconius butterflies : the singular evolution of an adaptive suite. *Proc. R. Soc. B Biol. Sci.* **287**, (2020).
54. Farris, S. M. Evolution of complex higher brain centers and behaviors: Behavioral correlates of mushroom body elaboration in insects. *Brain. Behav. Evol.* **82**, 9–18 (2013).
55. Sivinski, J. Mushroom body development in nymphalid butterflies: A correlate of learning? *J. Insect Behav.* **2**, 277–283 (1989).
56. Montgomery, S. H., Merrill, R. M. & Ott, S. R. Brain composition in Heliconius butterflies, posteclosion growth and experience-dependent neuropil plasticity. *J. Comp. Neurol.* **524**, 1747–1769 (2016).
57. Couto, A., Young, F. & Stephen, M. Muschroom body expantion in Heliconiini. *prep.*
58. Sahu, M. R. & Mondal, A. C. Neuronal Hippo signaling: From development to diseases. *Dev. Neurobiol.* **81**, 92–109 (2021).
59. Poon, C. L. C., Mitchell, K. A., Kondo, S., Cheng, L. Y. & Harvey, K. F. The Hippo Pathway Regulates Neuroblasts and Brain Size in Drosophila melanogaster. *Curr. Biol.* **26**, 1034–1042 (2016).
60. Ding, R., Weynans, K., Bossing, T., Barros, C. S. & Berger, C. The Hippo signalling pathway maintains quiescence in Drosophila neural stem cells. *Nat. Commun.* **7**, (2016).
61. Feng, X. *et al.* A Platform of Synthetic Lethal Gene Interaction Networks Reveals that the GNAQ Uveal Melanoma Oncogene Controls the Hippo Pathway through FAK. *Cancer Cell* **35**, 457-472.e5 (2019).
62. Cao, F., Miao, Y., Xu, K. & Liu, P. Lethal (2) giant larvae: An indispensable regulator of cell polarity and cancer development. *Int. J. Biol. Sci.* **11**, 380–389 (2015).
63. Kaya-çopur, A. *et al.* The hippo pathway controls myofibril assembly and muscle fiber growth by regulating sarcomeric gene expression. *Elife* **10**, 1–34 (2021).
64. Manning, B. D. & Toker, A. AKT/PKB Signaling: Navigating the Network. *Cell* **169**, 381–405 (2017).
65. Abeysundara, N., Simmonds, A. J. & Hughes, S. C. Moesin is involved in polarity maintenance and cortical remodeling during asymmetric cell division. *Mol. Biol. Cell* **29**, 419–434 (2018).
66. Wang, X., Zhang, Y. & Blair, S. S. Fat-regulated adaptor protein Dlish binds the growth suppressor Expanded and controls its stability and ubiquitination. *Proc. Natl. Acad. Sci. U. S. A.* **116**, 1319–1324 (2019).
67. Bahrampour, S. & Thor, S. Ctr9, a key component of the paf1 complex, affects proliferation and terminal differentiation in the developing drosophila nervous system. *G3 Genes, Genomes, Genet.* **6**, 3229–3239 (2016).
68. Sousa-Nunes, R., Chia, W. & Somers, W. G. Protein Phosphatase 4 mediates localization of the Miranda complex during

Drosophila neuroblast asymmetric divisions. *Genes Dev.* **23**, 359–372 (2009).

69. Yang, M. *et al.* Glia-derived temporal signals orchestrate neurogenesis in the Drosophila mushroom body. *Proc. Natl. Acad. Sci. U. S. A.* **118**, 1–12 (2021).
70. Loyer, N. & Januschke, J. Where does asymmetry come from? Illustrating principles of polarity and asymmetry establishment in Drosophila neuroblasts. *Curr. Opin. Cell Biol.* **62**, 70–77 (2020).
71. Blair, S. & McNeill, H. Big roles for Fat cadherins. *Curr. Opin. Cell Biol.* **51**, 73–80 (2018).
72. Yildirim, K., Petri, J., Kottmeier, R. & Klämbt, C. Drosophila glia: Few cell types and many conserved functions. *Glia* **67**, 5–26 (2019).
73. Gai, T. *et al.* Cocoonase is indispensable for Lepidoptera insects breaking the sealed cocoon. *PLoS Genet.* **16**, 1–16 (2020).
74. Smith, G. *et al.* Evolutionary and structural analyses uncover a role for solvent interactions in the diversification of cocoonases in butterflies. *Proc. R. Soc. B Biol. Sci.* **285**, (2018).
75. Baek, M. *et al.* Accurate prediction of protein structures and interactions using a three-track neural network. *Science (80-.).* **373**, 871–876 (2021).
76. Ruiz-Serra, V. *et al.* Assessing the accuracy of contact and distance predictions in CASP14. *Proteins Struct. Funct. Bioinforma.* **89**, 1888–1900 (2021).
