# Supplementary material for: Evolutionary dynamics of genome size and content during the adaptive radiation of Heliconiini butterflies
Source: Nat Commun. 2023 Sep 12;14:5620. doi: 10.1038/s41467-023-41412-5 (PMC10497600; doi:10.1038/s41467-023-41412-5)
Supplement: Supplementary file 3 — Reporting Summary [file 41467_2023_41412_MOESM3_ESM.pdf]

nature portfolio

Corresponding author(s): Francesco Cicconardi

Last updated by author(s): 04/08/2023

Reporting Summary

Nature Portfolio wishes to improve the reproducibility of the work that we publish. This form provides structure for consistency and transparency in reporting. For further information on Nature Portfolio policies, see our Editorial Policies and the Editorial Policy Checklist.

Statistics

For all statistical analyses, confirm that the following items are present in the figure legend, table legend, main text, or Methods section.

n/a	Confirmed	
☐	☒	The exact sample size (*n*) for each experimental group/condition, given as a discrete number and unit of measurement
☒	☐	A statement on whether measurements were taken from distinct samples or whether the same sample was measured repeatedly
☐	☒	The statistical test(s) used AND whether they are one- or two-sided<br>*Only common tests should be described solely by name; describe more complex techniques in the Methods section.*
☒	☐	A description of all covariates tested
☐	☒	A description of any assumptions or corrections, such as tests of normality and adjustment for multiple comparisons
☐	☒	A full description of the statistical parameters including central tendency (e.g. means) or other basic estimates (e.g. regression coefficient) AND variation (e.g. standard deviation) or associated estimates of uncertainty (e.g. confidence intervals)
☐	☒	For null hypothesis testing, the test statistic (e.g. *F*, *t*, *r*) with confidence intervals, effect sizes, degrees of freedom and *P* value noted<br>*Give P values as exact values whenever suitable.*
☐	☒	For Bayesian analysis, information on the choice of priors and Markov chain Monte Carlo settings
☐	☒	For hierarchical and complex designs, identification of the appropriate level for tests and full reporting of outcomes
☐	☒	Estimates of effect sizes (e.g. Cohen's *d*, Pearson's *r*), indicating how they were calculated

Our web collection on statistics for biologists contains articles on many of the points above.

Software and code

Policy information about availability of computer code

Data collection	No software was used to collect data
Data analysis	All statistical analyses were performed using open source data in R, including the following packages: PHYTOOLS; CAPER; GOSTATS; DCT & BLT; Bio3D. In addition we used the open source softwares HIFIASM V0.12-r304; CANU V1.8+356 changes; ARROW V2.3.3; STAR V2.7.2c, PILON V1.23; PURGE HAPLOTIGS V20191008; POLAR STAR V1; P_RNA_SCAFFOLDER V1; LRSCAF V1.1.5; LR_GAPCLOSER v.1.1; BLAST v2.7.1+; ALLMAPS V1; SUPERNOVA V2.1.1; BUSCO V3.1.0; TIGMINT V1.1.2; LRSCAF V1; ARCS V1.1.0; MINIMAP2 V2.17-r974-dirty; BOWTIE2 V2.2.1; SAMTOOLS V1.14; SPADES V3.15; AMOSCMP V3.1.0; GATK V3.1; PICARD V2.26; BCFTOOLS V0.1.19; SOAPDENOVO2 VR240; BBMAP V38.79; HISAT2 V2.1.0; RAGOO V1; ABYSS-SEALER V2.2.2; BLOBTOOLS V1.1.1; EXONERATE V2.46.2; MACSE V2.03; FASTTREE V2.1.11 SSE3; CACTUS V1.3; TRIMMOMATIC V0.39; BRAKER V2.1.5; GENEMARK-ES SUITE V4.30; AUGUSTUS V3.4.0; REPEATMASKER V4.1.1; GUSHR V1.1.0; TRINITY V2.10.0; DELTABLAST V.2.7.1+; HMMSCAN V3.3.2; STRINGTIE V2.1.3B; CUFFLINKS V2.2.1; PORTCULLIS V1.1.2; MIKADO V2.3.3; TRANSDECODER V5.5.0; COMPARATIVE ANNOTATION TOOLKIT V2.2.1; MALIN (Mac OS X version); BROCCOLI V1.1; DAMA V2; CAFE V5.0; PREQUAL V1.02; MACSE V2.03; HMMCLEANER V1; GBLOCKS V0.91B; IQ-TREE2 V2.1.3 COVID-EDITION; HYPHY V2.5; GOATOOLS v1; GENEMANIA prediction server; ASTRAL-III v5.6.3; TRIPVOTE V1.2; MCMCTREE V4.8A; TRACER V1.7.1; HALTOOLS SUITE V2.2; TRIMAL V1.4.REV22; CLUSTALW v1.2.1; FASTTREE V2.1.11; MIPHY v1.1.2; PHAST V1.4; PhyloAcc-GT v1; BEDTOOLS V2.30.0; ; TOPCONS webserver v2.0; CDSEARCH webserver; SIGNALP v5.0b; HHPRED server; ROSETTAFOLD v1.1.0; PYMOL v1.20. The custom python scripts: BRAKER-GUSHRGFF3TOBED12V1.0.PY; IDENDITYFUSEDTRANSCRIPTS.PY; IDENDITYFUSEDTRANSCRIPTS.PY; BROCCOLICHIMERASPLITDATAGATHER.PY; REMOVEINPARALOGFROMTREE.PY available at https://github.com/francicco/-ComparativeGenomicsOfHeliconiini.

For manuscripts utilizing custom algorithms or software that are central to the research but not yet described in published literature, software must be made available to editors and reviewers. We strongly encourage code deposition in a community repository (e.g. GitHub). See the Nature Portfolio guidelines for submitting code & software for further information.

Data

Policy information about availability of data

All manuscripts must include a data availability statement. This statement should provide the following information, where applicable:

- Accession codes, unique identifiers, or web links for publicly available datasets
- A description of any restrictions on data availability
- For clinical datasets or third party data, please ensure that the statement adheres to our policy

The following SRA datasets were used: ERR5235460; ERR5235459; ERR5235454; ERR5235452; SRR8883890; ERR5235468; SRR8883900; SRR4032079; SRR4032054; SRR8883898; SRR4032055; ERR5235453; ERR5235458; ERR5235467; SRR8883893; SRR4032011; ERR5235456; ERR5235455; SRR3102171; SRR3102172; SRR3102337; ERR266262; ERR1143583; ERR1143585; ERR1143626; ERR5235461; SRR8883892; ERR1143625; SRR8883889; SRR8883908; ERR1143586; ERR260305; SRR8883891; SRR8883894; ERR5235451; SRR8883896; ERS977714; ERR3653294; SRR3102172

New data was uploaded on NCBI under these ids: PRJNA686707; PRJNA686711; PRJNA686710; PRJNA686708; PRJNA686713; PRJNA686712; PRJNA686712; PRJNA686714; PRJNA686715

Human research participants

Policy information about studies involving human research participants and Sex and Gender in Research.

Reporting on sex and gender	not applicable
Population characteristics	not applicable
Recruitment	not applicable
Ethics oversight	not applicable

Note that full information on the approval of the study protocol must also be provided in the manuscript.

Field-specific reporting

Please select the one below that is the best fit for your research. If you are not sure, read the appropriate sections before making your selection.

☒ Life sciences ☐ Behavioural & social sciences ☐ Ecological, evolutionary & environmental sciences

For a reference copy of the document with all sections, see nature.com/documents/nr-reporting-summary-flat.pdf

Life sciences study design

All studies must disclose on these points even when the disclosure is negative.

Sample size	For our phylogenetic/genomic sampling we collected one individual per taxa. Our analyses are focused on macroevolution events therefore one genome per species is enough to account for deeper phylogenetic differences.
Data exclusions	We did not exclude any species, but we actually tried to collect as many species as possible
Replication	All statistical analyses were repeated to check for model convergence, where appropriate. Bayesian and Maximum likelihood analyses were run multiple times to evaluate convergence of consistency in results. We also run some of the analyses using different parameters/options to evaluate the robustness of the results.
Randomization	Samples were allocated to groups based on their species identify and taxonomic order.
Blinding	Blinding was not possible as for the genomic data and cannot be applied to this kind of study.

Reporting for specific materials, systems and methods

We require information from authors about some types of materials, experimental systems and methods used in many studies. Here, indicate whether each material, system or method listed is relevant to your study. If you are not sure if a list item applies to your research, read the appropriate section before selecting a response.

Materials & experimental systems

n/a	Involved in the study
☒	☐ Antibodies
☒	☐ Eukaryotic cell lines
☒	☐ Palaeontology and archaeology
☐	☒ Animals and other organisms
☒	☐ Clinical data
☒	☐ Dual use research of concern

Methods

n/a	Involved in the study
☒	☐ ChIP-seq
☒	☐ Flow cytometry
☒	☐ MRI-based neuroimaging

Animals and other research organisms

Policy information about studies involving animals; ARRIVE guidelines recommended for reporting animal research, and Sex and Gender in Research

Laboratory animals	The following species were obtained from commercial pupae suppliers for additional neuroantomical study:<br>Speyeria mormonia ssp washingtonia<br>Dryas julias<br>Dryadula phaetusa<br>Dione juno<br>A. vanillae incarnata (Florida)<br>A. v. vanillae (Costa Rica)
Wild animals	We sample taxa from the clade of Heliconiinae butterflies, all information on collection and export permits is provided in the supplementary material. Wild caught butterflies were sampled for DNA extraction.They were killed by decapitation with the head being fixed for subsequent dissections. All dissections were performed in the field at the site of capture. Body tissue and wings were kept as voucher specimens. Wild caught species and their sample sizes are as below<br>Philaethria dido 1<br>Podothricha telesiphe 7<br>Heliconius doris 2<br>Heliconius aoede 5
Reporting on sex	We sample both male and females when possible.
Field-collected samples	Field collected samples are detailed above.
Ethics oversight	none required for insect studies, beyond relevant collection and exportation permits.

Note that full information on the approval of the study protocol must also be provided in the manuscript.
