# Supplementary material for: Evolutionary dynamics of genome size and content during the adaptive radiation of Heliconiini butterflies
Source: Nat Commun. 2023 Sep 12;14:5620. doi: 10.1038/s41467-023-41412-5 (PMC10497600; doi:10.1038/s41467-023-41412-5)
Supplement: Supplementary file 4 — Description of additional supplementary files [file 41467_2023_41412_MOESM4_ESM.pdf]

Description of Additional Supplementary Files

File Name: Supplementary Data 1
Description: Description of the assembly and were data were obtained or submitted.

File Name: Supplementary Data 2
Description: Accession number of SRA used to reannotate available and new genome assemblies.

File Name: Supplementary Data 3
Description: Node constraints used and results from MCMCtree analysis.

File Name: Supplementary Data 4
Description: Introgression event detected and their time estimations.

File Name: Supplementary Data 5
Description: Output data deom DCT/BTL introgression analyses.

File Name: Supplementary Data 6
Description: Tables showing the test for convergence in CAFE analyses.

File Name: Supplementary Data 7
Description:Orthologous group expantions/contractions and homology assignment to D. melanogaster.

File Name: Supplementary Data 8
Description: Gene family expantions/contraction across Heliconiini.

File Name: Supplementary Data 9
Description: Diversifying selection on scOGs using aBSREL.

File Name: Supplementary Data 10
Description: GO term enrichment analysis showing intersection between Gostats and GOATOOLS.

File Name: Supplementary Data 11
Description: GO term enrichment analysis using GeneMania.

File Name: Supplementary Data 12
Description: CNEEs gene enrichment analysis.

File Name: Supplementary Data 13
Description: Genomic spatial enrichment analysis.

File Name: Supplementary Data 14
Description: GO term enrichment analysis of aCNEEs.

File Name: Supplementary Data 15
Description: Cocoonases annotations and dataset description.

File Name: Supplementary Data 16
Description: Cocoonases synteny description.
